# Transcriptional profiling identifies the long noncoding RNA plasmacytoma variant translocation (*PVT1*) as a novel regulator of the asthmatic phenotype in human airway smooth muscle

**DOI:** 10.1016/j.jaci.2016.06.014

**Published:** 2017-03

**Authors:** Philip J. Austin, Eleni Tsitsiou, Charlotte Boardman, Simon W. Jones, Mark A. Lindsay, Ian M. Adcock, Kian Fan Chung, Mark M. Perry

**Affiliations:** a Airways Disease, National Heart and Lung Institute, Imperial College, London & Royal Brompton NIHR Biomedical Research Unit, London, United Kingdom; b Respiratory Research Group, University Hospital of South Manchester, University of Manchester, Manchester, United Kingdom; c Institute of Inflammation and Ageing, University of Birmingham, Birmingham, United Kingdom; d Department of Pharmacy and Pharmacology, University of Bath, Bath, United Kingdom; e Dubowitz Neuromuscular Centre, Molecular Neurosciences Section, Developmental Neurosciences Programme, UCL Institute of Child Health, London, United Kingdom

**Keywords:** Asthma, airway smooth muscle, proliferation, IL-6, transcriptome, long noncoding RNA, *PVT1*, ASM, Airway smooth muscle, ASMC, Airway smooth muscle cell, BrdU, Bromodeoxyuridine, cRNA, Coding RNA, lncRNA, Long noncoding RNA, miRNA, MicroRNA, ncRNA, Noncoding RNA, PVT1, Plasmacytoma variant translocation, siRNA, Small interfering RNA

## Abstract

**Background:**

The mechanism underlying nonsevere and severe asthma remains unclear, although it is commonly associated with increased airway smooth muscle (ASM) mass. Long noncoding RNAs (lncRNAs) are known to be important in regulating healthy primary airway smooth muscle cells (ASMCs), whereas changed expression has been observed in CD8 T cells from patients with severe asthma.

**Methods:**

Primary ASMCs were isolated from healthy subjects (n = 9) and patients classified as having nonsevere (n = 9) or severe (n = 9) asthma. ASMCs were exposed to dexamethasone and FCS. mRNA and lncRNA expression was measured by using a microarray and quantitative real-time PCR. Bioinformatic analysis was used to examine relevant biological pathways. Finally, the lncRNA plasmacytoma variant translocation 1 (PVT1) was inhibited by transfection of primary ASMCs with small interfering RNAs, and the effect on ASMC phenotype was examined.

**Results:**

The mRNA expression profile was significantly different between patient groups after exposure to dexamethasone and FCS, and these were associated with biological pathways that might be relevant to the pathogenesis of asthma, including cellular proliferation and pathways associated with glucocorticoid activity. We also observed a significant change in lncRNA expression, yet the expression of only one lncRNA (PVT1) is decreased in patients with corticosteroid-sensitive nonsevere asthma and increased in patients with corticosteroid-insensitive severe asthma. Subsequent targeting studies demonstrated the importance of this lncRNA in controlling both proliferation and IL-6 release in ASMCs from patients with severe asthma.

**Conclusions:**

lncRNAs are associated with the aberrant phenotype observed in ASMCs from asthmatic patients. Targeting PVT1 might be effective in reducing airway remodeling in asthmatic patients.

Asthma is characterized by airflow obstruction and chronic airway inflammation and remodeling.^1^ Airway smooth muscle (ASM) hyperplasia and hypertrophy lead to increased airway wall thickening and airway narrowing, contributing to airway obstruction and inflammation. Epigenetic mechanisms are key regulators of ASM function.^2^ We have reported that the aberrant phenotype of airway smooth muscle cells (ASMCs) from asthmatic patients is under the negative regulation of the cyclin inhibitors p21^WAF1^ and p27^kip1^, which are controlled by microRNA-221.^3^ Furthermore, using a transcriptomics-based approach, we identified potential microRNA (miRNA) targets and pathways in activated human ASMCs and observed changes in expression of long noncoding RNAs (lncRNAs), including natural antisense, pseudogenes, intronic lncRNAs, and intergenic lncRNAs.^4^

Having reported results with healthy (or nonasthmatic) ASMCs in 2014,^4^ we now report on the asthmatic ASMCs that were studied at the same time. Specifically, we report on the differential expression of mRNAs and lncRNAs in ASMCs isolated from patients with nonsevere and severe asthma and treated with dexamethasone before subsequent activation with the growth medium FCS. We have then focused on the only lncRNA found to be decreased in expression in patients with corticosteroid-sensitive nonsevere asthma and increased in expression in patients with corticosteroid-insensitive severe asthmatics, plasmacytoma variant translocation 1 *(PVT1)*, and performed targeting studies to examine its role in a corticosteroid insensitivity model in these cells induced by the combination of TGF-β and FCS.

## Methods

Full methodology is available in the Methods section in this article's Online Repository at www.jacionline.org.

### Subject selection and ASMC culture

**Fig E1:**
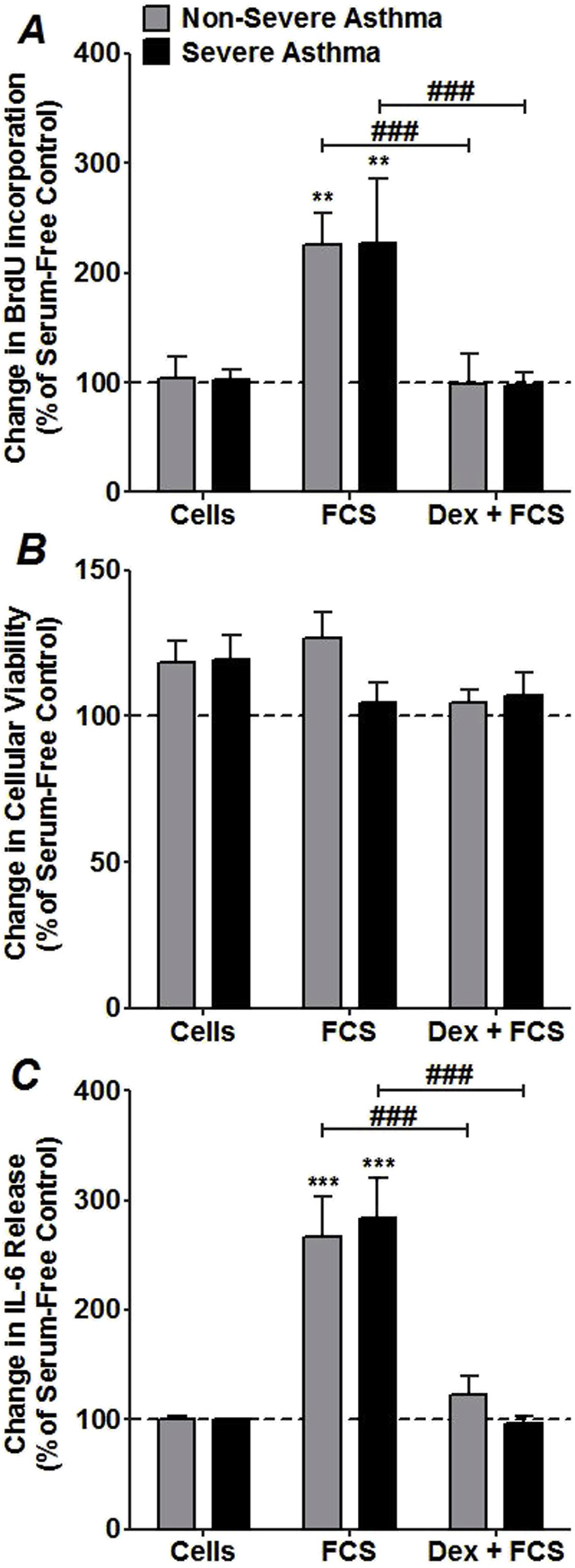


Primary ASMCs from healthy subjects, patients with nonsevere asthma, and patients with severe asthma were treated, as described previously,^4^, ^5^, ^6^, ^7^ and proliferation rates, cellular viability, and IL-6 release (see Fig E1 in this article's Online Repository at www.jacionline.org), were all comparable to our previous studies.^1^, ^2^, ^3^ Patients' characteristics are shown in Table I.

### RNA extraction

RNA was extracted with the *mir*Vana miRNA isolation kit, as previously described.^3^, ^4^, ^5^, ^6^, ^7^

### Microarray analysis

lncRNA and mRNA expression was determined by using Agilent SurePrint G3 Human GE microarrays (Agilent Technologies, Santa Clara, Calif), according to the manufacturer's instructions, and as previously described.^4^ Total RNA samples (50 ng) used in lncRNA and mRNA microarrays were initially labeled with Spike-In control A (for cyanine 3–cytidine triphosphate) or B (for cyanine 5–CTP). Labeled samples were then used for cDNA synthesis by using the cDNA Master Mix (Agilent Technologies) and incubated for 2 hours at 40°C, followed by 15 minutes at 70°C to inactivate the Affinity-Script enzyme. The synthesized cDNA was then used for coding RNA (cRNA) synthesis and amplification by using the Transcription Master Mix with either cyanine 3 or cyanine 5 and incubated at 40°C for 2 hours. Labeled and amplified RNA was then purified with the RNeasy Mini Kit (Qiagen, Hilden, Germany) and quantified with the NanoDrop Spectrophotometer's Microarray Measurement function (NanoDrop, Wilmington, Del). The cyanine 3 or cyanine 5 concentrations and the cRNA concentration were used to calculate the yield (in micrograms) and specific activity (in picomoles of cyanine 3 or cyanine 5 per microgram of cRNA) of each sample. For each microarray reaction, 300 ng of cyanine 3–labeled and 300 ng of cyanine 5–labeled linearly amplified cRNA samples were mixed and incubated together with fragmentation buffer for 30 minutes, followed by addition of hybridization buffer. Samples were loaded onto SurePrint G3 (8 × 60 K) microarray slides (Agilent Technologies) and hybridized at 65°C for 17 hours at 10 rpm by using Agilent's Hybridization oven and SureHyb chamber. The microarrays were then disassembled and washed in GE Wash Buffer 1 (Agilent Technologies) for 60 seconds at room temperature, followed by GE Wash Buffer 2 for 60 seconds at 37°C, followed by an acetonitrile wash (10 seconds at room temperature) and a final wash in stabilization and drying solution for 30 seconds at room temperature, to improve microarray results by preventing ozone-mediated fluorescent signal degradation. The microarrays were scanned with the Agilent Microarray Scanner G2565BA by using the profile for 2-color microarrays (AgilentG3_GX_2Color) at 5-μm resolution (dye channel, Red&Green; scan area, 61 × 21.6 mm).

After normalization against internal controls provided within the labelling kits, probes with background expression (signal value of mRNA < 4.5) were removed. The threshold of background expression was determined by using samples that were processed but contained no RNA. Because initial analysis of mRNA and lncRNA between baseline and FCS-treated cells or dexamethasone plus FCS–treated cells showed none that produced a false discovery rate of less than 0.1, differential expression (*P* value) was determined by using 3-way ANOVA with the Partek Genomics Suite. We report changes in expression at a *P* value of less than .05.

### Pathway analysis

Differentially expressed mRNAs from each data set were further analyzed by using the bioinformatics software Ingenuity Pathway Analysis application (www.ingenuity.com). A core functional analysis was performed to identify canonical pathways, predicted upstream regulators, and gene networks most associated significantly with the differentially expressed mRNAs.

The significance of the association of a given canonical pathway with the differentially expressed mRNAs was measured in 2 ways: (1) based on the ratio of the number of differentially expressed mRNAs in the data set that mapped to the canonical pathway divided by the total number of genes that map to the canonical pathway and (2) by using the Fisher exact test to calculate a *P* value for the association between the mRNA and network/canonical pathway.

### Quantitative PCR measurement of miRNA and mRNA expression

lncRNA and mRNA expression was measured, as previously described.^4^

### Transfection with small interfering RNAs that target *PVT1*

ASMCs were transfected, as previously described.^3^, ^5^, ^8^ Small interfering RNAs (siRNAs) designed to target *PVT1* and *IL6* were purchased from Ambion/Applied Biosystems, Waltham, Mass. ASMCs were transfected with *PVT1* inhibitor (30, 100, or 300 nmol/L), *IL6* inhibitor (100 nmol/L), or Silencer Negative Control #1 (100 nmol/L) and no siRNA (mock transfection).

### Data and statistical analysis

Data were analyzed with GraphPad Prism software, version 5.03 (GraphPad Software, La Jolla, Calif). Data were not normally distributed (as assessed by using the Kolmogorov-Smirnov test), and therefore groups were compared with the Dunn nonparametric test. All data are expressed as means ± SEMs.

## Results

### mRNAs are differentially expressed between ASMCs from patients with nonsevere and those with severe asthma and are responsible for different pathway activation

Comparison of mRNA expression in ASMCs from healthy subjects and patients with nonsevere or severe asthma showed differential expression (*P* < .05) of different gene sets depending on the status of the cells being at a “baseline” state after stimulation with FCS and those pretreated with dexamethasone before stimulation with FCS. Full gene lists are shown in Tables E1-E9 in this article's Online Repository at www.jacionline.org.

**Fig 1:**
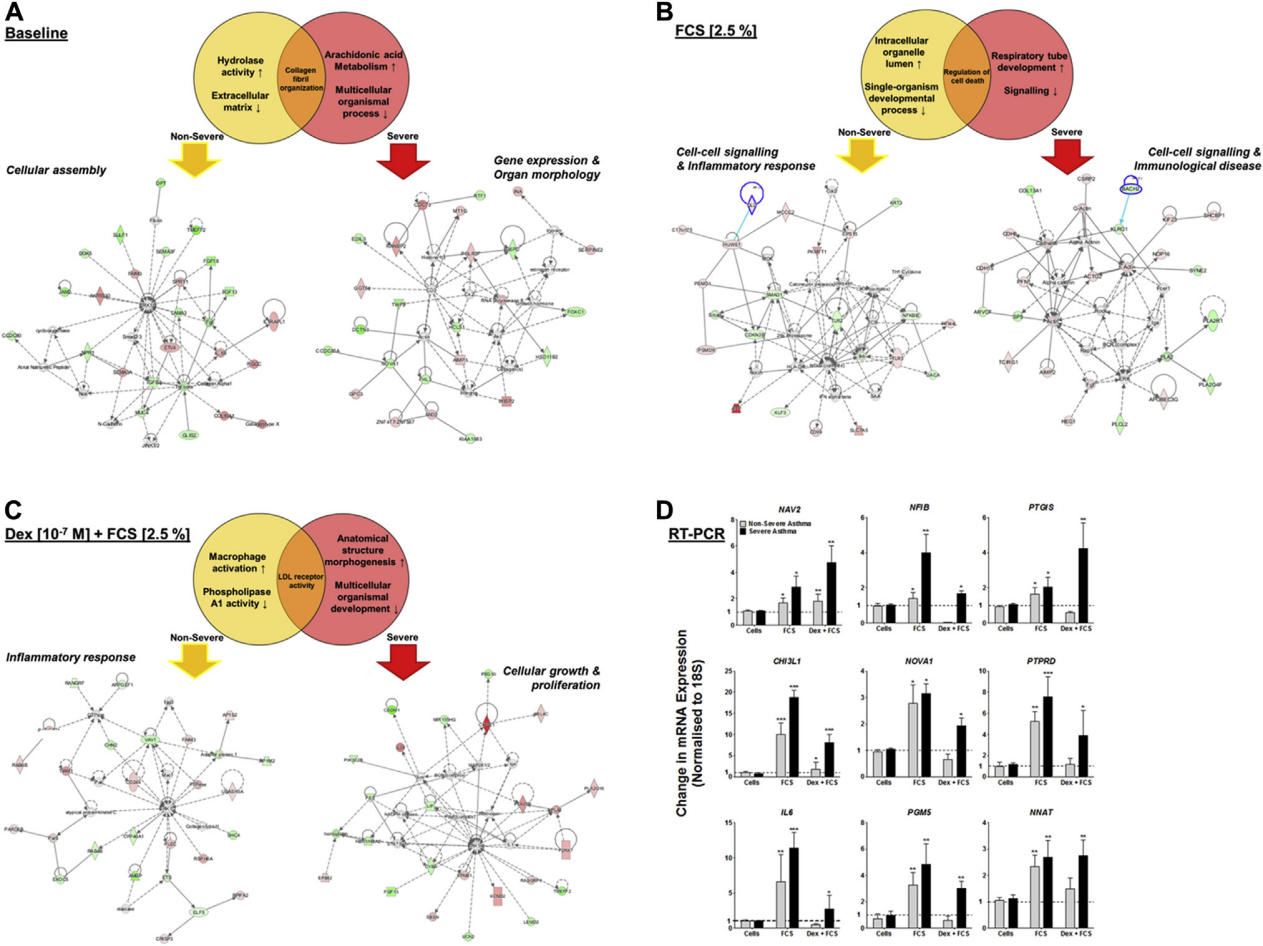
Venn diagrams showing pathway analysis of differentially expressed mRNAs in ASM from asthmatic patients. **A-C,** Pathway analysis showing intergroup comparisons at baseline (Fig 1, *A*), after stimulation with FCS (2.5%; Fig 1, *B*), and after stimulation with FCS (2.5%) plus TGF-β (1 ng/mL; Fig 1, *C*). Data represent 9 members of each patient type. **D,** Expression of 9 mRNAs was confirmed by using TaqMan RT-PCR to validate the array data. *Bars* represent means ± SEMs from 9 primary ASMC donors. *P < .05, **P < .01, and ***P < .001.

To identify the pathways in which these mRNAs are involved, we analyzed each data set by using the bioinformatics software application Ingenuity Pathway Analysis. At baseline, those mRNAs that were increased in expression compared with ASMCs from healthy subjects in the nonsevere asthma cohort are proposed to be important in hydrolase activity. Those decreased in expression are involved in the extracellular matrix, and the whole profile is important for cellular assembly (Fig 1, *A*). Interestingly, hydrolase activity is known to be important for calcium signaling in ASM,^9^ and the extracellular matrix is involved in asthmatic airway remodeling.^10^ In the ASMCs from the patients with severe asthma, these profiles changed to an increase in genes necessary for arachidonic acid metabolism and a decrease in those required for multicellular organismal process, and the whole profile is involved in gene expression and organ morphology. Once again, this follows our current knowledge of ASM function and the fact that arachidonic acid induces calcium influx in human airway smooth muscle, which has been proposed to contribute to increased influx in asthmatic patients,^11^ and previous methylation profiling of the bronchial mucosa of asthmatic patients has demonstrated a positive relationship to atopy for the multicellular organismal process.^12^ After stimulation with FCS, those mRNAs increased in expression in the patients with nonsevere ASM are associated with the intracellular organelle lumen, those decreased are involved in single-organism development, and together the profile is important in the inflammatory response that is well documented in human ASM (Fig 1, *B*).^13^ In ASMCs from patients with severe asthma, those with increased expression are involved in respiratory tube development, and those with decreased expression are involved in signaling. However, together, the mRNAs are proposed to be important in patients with immunologic disease, which helps explain how ASM is important for immunomodulation in acute exacerbations of airway disease, including asthma.^14^ When these same ASM cohorts were pretreated with dexamethasone, before stimulation with FCS (Fig 1, *C*), those pathways activated in ASMCs from patients with nonsevere asthma were associated with glucocorticoid activity, such as macrophage activation, the inflammatory response,^15^ and phospholipase activity.^16^ However, ASMCs from patients with severe asthma responded by activating genes associated with cellular growth and proliferation, a phenotype well documented as being aberrant.^3^, ^4^, ^5^, ^6^

Levels of *NAV2*, *NFIB*, *PTGIS*, *CHI3L1*, *NOVA1*, *PTPRD*, *IL6*, *PGM5*, and *NNAT* were selected to be verified by means of quantitative RT-PCR (Fig 1, *D*). For each of these, FCS caused a significant increase in expression in ASMCs from both patients with nonsevere and those with severe asthma (*P* < .05). Pretreatment of ASMCs from patients with nonsevere asthma with dexamethasone inhibited this increase in expression in all mRNAs, apart from *NAV2*. In ASMCs from patients with severe asthma, dexamethasone caused a further increase in *NAV2* and *PTGIS* expression (*P* < .01); in all other instances dexamethasone only slightly inhibited the FCS-induced mRNA expression to levels that were not comparable with ASMCs from patients with nonsevere asthma.

### lncRNAs are differentially expressed between ASMCs from patients with nonsevere and those with severe asthma

We have previously shown that more than 30 lncRNAs are increased in expression in healthy primary ASMCs after treatment with dexamethasone and stimulation with FCS.^4^ Hence to identify novel lncRNAs in ASMCs from asthmatic patients, we used ENSEMBLE (www.ensembl.org/index.html) to determine the genomic position of those probe sets from the microarray that did not match known protein-coding genes.

At baseline, 21 lncRNAs were differentially expressed in ASMCs from patients with nonsevere asthma (15 increased and 6 decreased) when compared with healthy subjects (see Table E10 in this article's Online Repository at www.jacionline.org). ASMCs from patients with severe asthma differentially expressed 19 lncRNAs (13 increased and 6 decreased) when compared with healthy subjects (see Table E11 in this article's Online Repository at www.jacionline.org).^4^ Interestingly, 4 lncRNAs were altered in expression in both disease phenotypes (*RP5-1158E12.3*, *FKBP1A-SDCBP2*, *LINC00472*, and *PVT1*), and *PVT1* was also differentially expressed in ASMCs from healthy subjects.^4^ After stimulation with FCS, ASMCs from patients with nonsevere asthma expressed a completely different set of lncRNAs (15 increased and 16 decreased), with the exception of *PVT1* and *RP11-141M1* (see Table E12 in this article's Online Repository at www.jacionline.org), and treatment of the same cells with dexamethasone, before said stimulation resulted in an increase in expression of 60 lncRNAs, and a decrease in 19 (see Table E13 in this article's Online Repository at www.jacionline.org). A similar pattern was observed in FCS-stimulated ASMCs from patients with severe asthma; of the 32 lncRNAs that changed in expression, only 2 (*LINC00940* and *RP11-120D5.1*) were the same as seen in the baseline ASMCs from patients with nonsevere asthma (see Table E14 in this article's Online Repository at www.jacionline.org). Furthermore, when ASMCs from patients with severe asthma were treated with dexamethasone before subsequent stimulation with FCS, the number of differentially expressed lncRNAs doubled (36 increased and 38 decreased in expression, see Table E15 in this article's Online Repository at www.jacionline.org).

Of those lncRNAs that were expressed in ASMCs from asthmatic patients, only *PVT1* was found to be decreased in expression in the patients with corticosteroid-sensitive nonsevere asthma and increased in expression in patients with corticosteroid-insensitive severe asthma (see Tables E10 and E11). Therefore we further examined the function of this lncRNA.

### Effect of FCS and TGF-β on *PVT1* lncRNA expression in ASMCs from asthmatic patients

**Fig 2:**
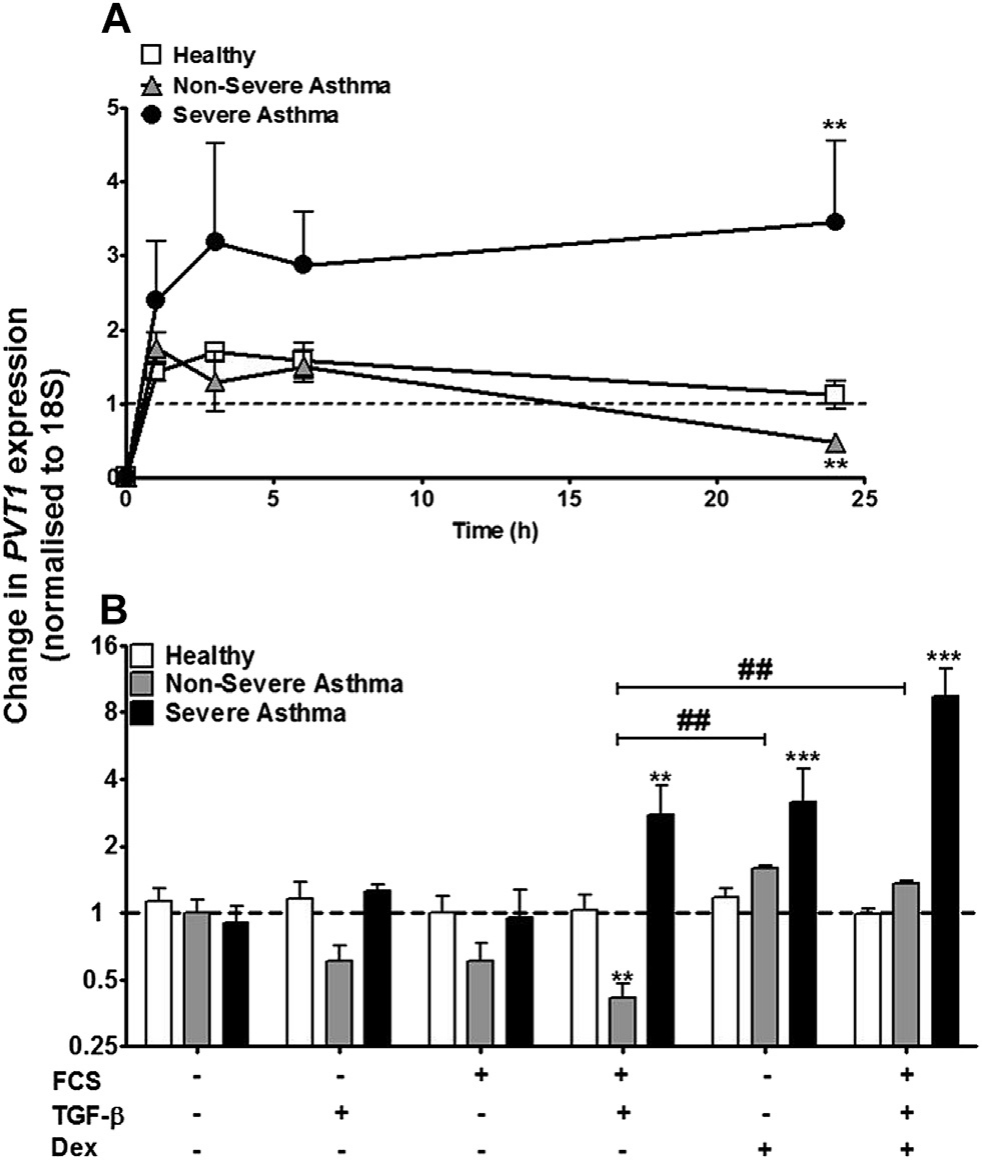
Effect of dexamethasone and FCS plus TGF-β on *PVT1* lncRNA expression in ASMCs from asthmatic patients. **A,** FCS plus TGF-β–induced *PVT1* lncRNA expression was measured by using quantitative RT-PCR over 1, 3, 6, and 24 hours. **B,** Dexamethasone *(Dex)* and FCS plus TGF-β–induced *PVT1* expression was measured by using quantitative RT-PCR at 24 hours. *Points/bars* represent means ± SEMs of 9 ASMC donors. **P < .01 and ***P < .001. ##*P* < .01.

We have previously demonstrated that combined stimulation with FCS (2.5%) and TGF-β (1 ng/mL) is required to induce a differential response in both cellular proliferation and cytokine release in ASMCs from asthmatic patients.^3^, ^5^ To determine the potential role of *PVT1* in this proliferative and inflammatory response, we examined the time course of its expression in the presence of FCS and TGF-β. FCS plus TGF-β did not change the expression of *PVT1* up to 24 hours in ASMCs from healthy subjects (Fig 2, *A*), whereas at 24 hours, there was a significant reduction in the expression of *PVT1* in ASMCs from patients with nonsevere asthma (*P* < .01 vs unstimulated control ASMCs). Furthermore, FCS plus TGF-β led to an approximately 3-fold increase (*P* < .01 vs unstimulated control ASMCs) in *PVT1* expression, which reached a plateau at 3 hours in ASMCs from patients with severe asthma and remained increased at 24 hours (Fig 2, *A*).

At 24 hours, dexamethasone had no effect on *PVT1* expression in ASMCs from healthy subjects (Fig 2, *B*). However, the FCS plus TGF-β–induced reduction of *PVT1* expression in ASMCs from patients with nonsevere asthma returned to basal levels in the presence of dexamethasone (*P* < .01). Furthermore, dexamethasone alone increased *PVT1* expression in ASMCs from patients with severe asthma (approximately 4-fold, *P* < .001), and when these cells were subsequently stimulated with FCS plus TGF-β, an even greater increase in expression was observed (approximately 8-fold, *P* < .001; Fig 2, *B*).

### Inhibition of *PVT1* with siRNAs on ASMC proliferation and IL-6 release

**Fig 3:**
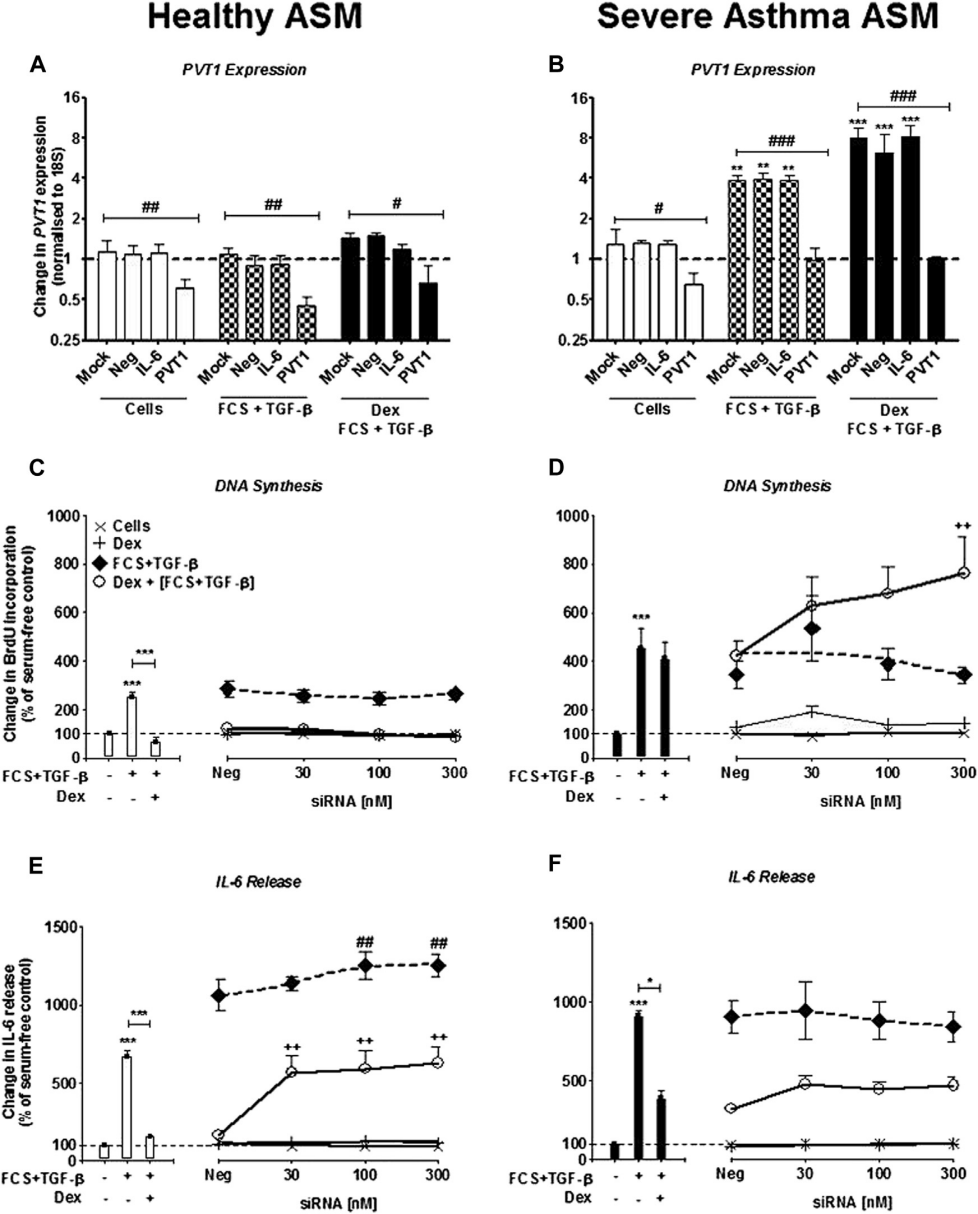
Effect of targeting *PVT1* with siRNAs on IL-6 release and BrdU incorporation in ASMCs from healthy subjects and patients with severe asthma. **A** and **B,***PVT1* lncRNA expression was measured by using RT-PCR after ASM from healthy subjects and patients with severe asthma was transfected with siRNA (300 nmol/L), which was designed to target *PVT1*. **C-F,** BrdU incorporation (Fig 3, *C* and *D*) and IL-6 release (Fig 3, *E* and *F*) were measured by using the BrdU ELISA and DuoSet ELISA, respectively, at 8 days. *Bars/points* represent means ± SEMs of 9 ASMC donors. */#*P* < .05, ##/++*P* < .01, and ***/###*P* < .001. *Dex*, Dexamethasone.

To elucidate the role of *PVT1*, we examined the action of siRNA-mediated inhibition of *PVT1* on dexamethasone-exposed, FCS plus TGF-β–induced bromodeoxyuridine (BrdU) incorporation and IL-6 release. As previously reported,^3^, ^5^ a significant increase in BrdU incorporation and IL-6 release was observed in both healthy subjects and patients with severe asthma after stimulation with FCS plus TGF-β (*P* < .001 vs unstimulated control ASMCs; Fig 3, *C-F*). Dexamethasone inhibited BrdU incorporation and IL-6 release in the healthy ASMCs but had no effect on BrdU incorporation in ASMCs from patients with severe asthma and a limited effect on IL-6 release (Fig 3, *C-F*). These results also demonstrate the relative corticosteroid insensitivity of the cells from patients with severe asthma compared with those from healthy subjects with respect to BrdU incorporation and IL-6 release.

Transfection with Amaxa electroporation with siRNAs designed to target *PVT1* (300 nmol/L) knocked down expression of *PVT1* in ASMCs from healthy subjects both at baseline and after stimulation with FCS plus TGF-β (*P* < .01; Fig 3, *A*). When the same cells were exposed to dexamethasone before stimulation with FCS plus TGF-β, a reduction was still observed, although not to the same level as without dexamethasone (*P* < .05). When *PVT1* was targeted with siRNAs in ASMCs from patients with severe asthma, the FCS plus TGF-β– and dexamethasone plus FCS plus TGF-β–induced *PVT1* expression was returned to basal levels (Fig 3, *B*).

Knocking down *PVT1* had no effect on BrdU incorporation induced by FCS plus TGF-β in either cohort of ASMCs (Fig 3, *C* and *D*). However, *PVT1* knockdown increased BrdU incorporation in ASMCs from patients with severe asthma when they were exposed to dexamethasone before stimulation with FCS plus TGF-β (*P* < .01 vs dexamethasone plus FCS plus TGF-β; Fig 3, *D*). Furthermore, siRNA-targeted inhibition of *PVT1* both resulted in an increase in FCS plus TGF-β–induced IL-6 release (*P* < .01 vs FCS plus TGF-β) and reversed the inhibitory action of dexamethasone in ASMCs from healthy subjects (*P* < .01 vs dexamethasone plus FCS plus TGF-β; Fig 3, *E*). This suggests that inhibiting expression of *PVT1* increases corticosteroid insensitivity in ASMCs; however, there was no effect observed on IL-6 release in ASMCs from patients with severe asthma when *PVT1* was targeted, although this might be because of IL-6 release already being at maximal levels (Fig 3, *F*).

**Fig E2:**
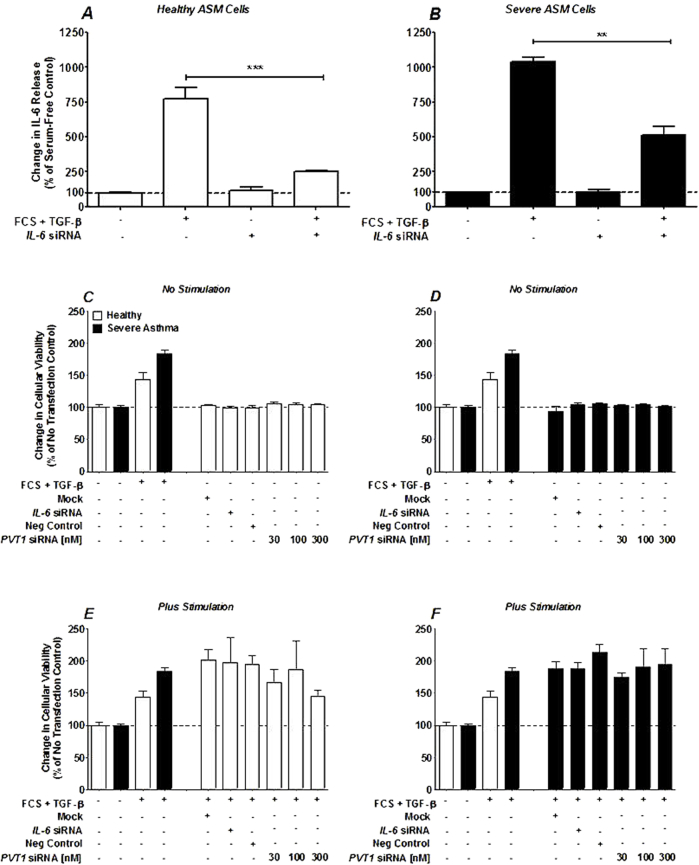


A nontargeting negative control (30-300 nmol/L) demonstrated no effect on either BrdU incorporation or IL-6 release in either cohort, as previously shown (data not shown).^3^, ^5^, ^8^ To demonstrate evidence of efficient transfection, concurrent studies were performed to examine the effect of siRNAs (100 nmol/L) targeted to *IL6* mRNA. A reduction in IL-6 release induced by FCS plus TGF-β stimulation in ASMCs from healthy subjects (*P* < .001) and those from patients with severe asthma (*P* < .01) was observed, as previously described (see Fig E2, *A* and *B*, in this article's Online Repository at www.jacionline.org).^3^, ^8^ Furthermore, to demonstrate that electroporation had no adverse effect on cellular viability, MTT (3-(4,5-dimethylthiazol-2-yl)-2,5-diphenyltetrazolium bromide) assays were performed both before (see Fig E2, *C* and *D*) and after (see Fig E2, *E* and *F*) stimulation with FCS plus TGF-β. In either situation, transfection by using Amaxa electroporation had no effect on cellular viability.

### Effect of *PVT1* inhibition on *IL6* mRNA expression in ASMCs from asthmatic patients

**Fig 4:**
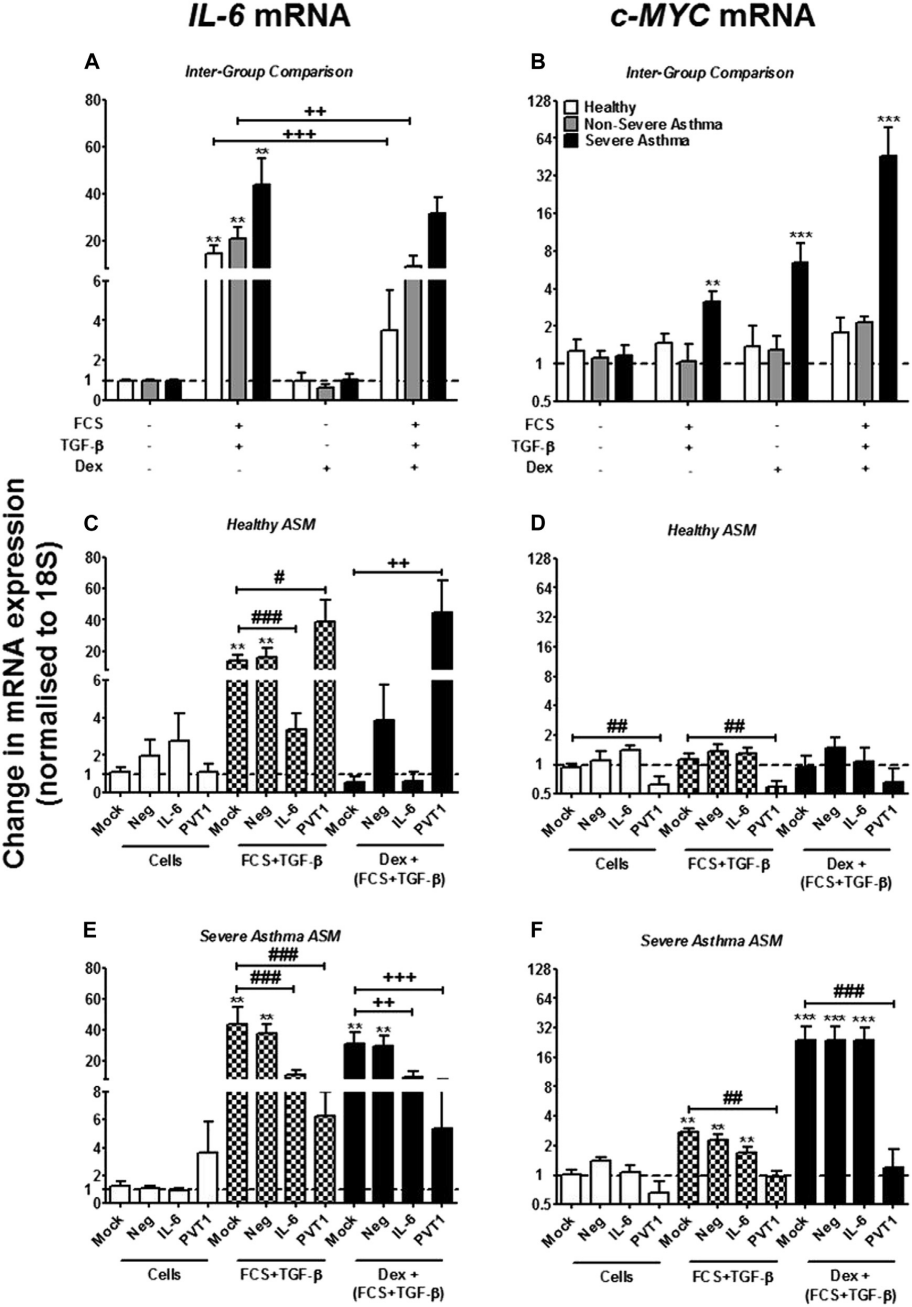
Effect of targeting *PVT1* with siRNAs on *IL6* and *c-MYC* mRNA in ASMCs from healthy subjects and patients with severe asthma. **A** and **B,***IL6* and *c-MYC* mRNA expression was measured by using RT-PCR after exposure to dexamethasone (*Dex*; 10^−7^ mol/L) and stimulation with FCS (2.5%) plus TGF-β (1 ng/mL) for 24 hours. **C-F,** ASMCs from healthy subjects and patients with severe asthma were transfected with siRNAs designed to target *PVT1*. Also, the expression of *IL6* (Fig 4, *C* and *E*) and *c-MYC* (Fig 4, *D* and *F*) mRNA was measured by using RT-PCR. *Bars* represent means ± SEMs of 9 ASMC donors. #*P* < .05, **/##/++*P* < .01, and ***/###/+++*P* < .001.

In a similar pattern to that seen in the increasing IL-6 protein release induced by FCS plus TGF-β in these ASMCs,^3^ *IL6* mRNA expression is increased in healthy cells (approximately 15-fold), greater still in cells from patients with nonsevere asthma (approximately 20-fold), and increased to the highest degree in cells from patients with severe ASMCs (approximately 45-fold) when these cells are stimulated with FCS plus TGF-β (*P* < .01 vs unstimulated control; Fig 4, *A*). Incubation with dexamethasone before stimulation with FCS plus TGF-β resulted in inhibition of *IL6* mRNA expression in ASMCs from healthy subjects and patients with nonsevere asthma (*P* < .001 and *P* < .01, respectively), with no significant effect in ASMCs from patients with severe asthma (Fig 4, *A*).

The effect of *PVT1* targeting by siRNAs on *IL6* mRNA was then examined. In ASMCs from healthy subjects, the FCS plus TGF-β–induced expression of *IL6* was similar to that observed in Fig 4, *A*, when the cells were either mock transfected or transfected with 100 nmol/L of negative control siRNA (Fig 4, *C*). When the healthy ASMCs were transfected with an siRNA designed to target *IL6* mRNA, the expression of this mRNA was almost completely attenuated (*P* < .001; Fig 4, *C*). However, when the basal expression of *PVT1* (as described in Fig 2, *B*), was inhibited in these ASMCs with 300 nmol/L siRNA, a significant increase in *IL6* mRNA expression was observed when the cells were stimulated with FCS plus TGF-β (*P* < .05; Fig 4, *C*). Addition of dexamethasone before FCS plus TGF-β resulted in a decrease in *IL6* mRNA levels in the mock and negative siRNA-transfected cells, and IL-6 siRNA transfection resulted in inhibition of *IL6* mRNA expression (Fig 4, *C*).

*IL6* mRNA expression was similarly affected by the different transfection variables in ASMCs from patients with severe asthma (Fig 4, *E*). Either mock transfection or transfection with a negative control siRNA had no effect on *IL6* mRNA expression. Transfecting with an siRNA designed to target *IL6* inhibited *IL6* mRNA expression (Fig 4, *E*). However, inhibiting the expression of *PVT1* with siRNAs reduced *IL6* mRNA expression after stimulation with FCS plus TGF-β, both with and without exposure to dexamethasone (*P* < .001; Fig 4, *E*).

### Effect of *PVT1* inhibition on *c-MYC* mRNA in ASMCs from asthmatic patients

*PVT1* is transcriptionally activated by the oncogene *c-MYC*,^4^, ^17^ and we have previously reported that *c-MYC* is important in controlling ASMC proliferation but not cytokine release.^5^ Therefore we examined the effect of targeting *PVT1* with siRNAs on *c-MYC* mRNA expression. Stimulation with FCS plus TGF-β induced an increase in *c-MYC* mRNA expression in ASMCs from patients with severe asthma (*P* < .01), with no effect seen in ASMCs from healthy subjects or patients with nonsevere asthma (Fig 4, *B*). Exposure of ASMCs from patients with severe asthma to dexamethasone, either on its own or before stimulation with FCS plus TGF-β, resulted in a larger increase in *c-MYC* mRNA expression (both *P* < .001; Fig 4, *B*).

Transfecting siRNAs designed to target *PVT1* (300 nmol/L) knocked down expression of *c-MYC* in healthy ASMCs both at baseline and after stimulation with FCS plus TGF-β (*P* < .01; Fig 4, *D*). When *PVT1* was targeted with siRNAs in ASMCs from patients with severe asthma, the FCS plus TGF-β– and dexamethasone plus FCS plus TGF-β–induced *c-MYC* expression was returned to basal levels (Fig 4, *F*).

In summary, these results show that in primary ASMCs from nonasthmatic subjects, inhibiting the endogenous expression of *PVT1* results in reversal of the inhibitory action of dexamethasone on FCS plus TGF-β–induced *IL6* mRNA expression and protein release. Furthermore, in ASMCs from patients with severe asthma, dexamethasone increases *PVT1* expression, and inhibition of this with siRNAs results in an increase in FCS plus TGF-β–induced cellular proliferation through targeting of the transcription factor *c-MYC*.

## Discussion

Using a transcriptomics-based approach, we examined the expression of RNAs (mRNA, miRNA, and lncRNA) in primary ASMCs isolated from nonasthmatic subjects, patients with nonsevere asthma, and patients with severe asthma. We published the results of the nonasthmatic subjects in 2014,^4^ and now, after some additional functional studies, report on the patients with nonsevere and severe asthma.

Of the mRNAs we examined, we found that the mRNA profile differentially regulated in ASMCs from patients with nonsevere asthma is important in ASMC calcium signaling,^9^ airway remodeling,^10^ the inflammatory response,^13^ and glucocorticoid activity.^15^, ^16^ Interestingly, the mRNA profile in ASMCs from patients with severe asthma appears to be important in increased calcium influx in the smooth muscle,^11^ atopy,^12^ and possible immunomodulation in patients with acute exacerbations of airway disease, including asthma.^14^ Furthermore, when confirming our array results using RT-PCR, we described mRNAs with expression that has previously been shown to be increased in response to dexamethasone *(NAV2)*^18^ to regulate cell viability, cell growth, cellular proliferation, and/or airway remodeling (*NFIB*, *NOVA1*, *PTPRD*, and *PGM5*)^19^, ^20^, ^21^, ^22^ and those acting as potential tumor suppressors in patients with lung carcinoma (*PTPRD* and *NNAT*).^23^, ^24^ Furthermore, we describe a large increase in *CHI3L1* expression, which has not only been reported in a number of patients with inflammatory diseases and cancer but also has been implicated in asthmatic patients, with the discovery of an allele that doubles the risk.^25^ However, *PTGIS* is the only one of these mRNA targets to have been associated with ASM and can induce both bronchodilation and reverse ASMC remodeling in a mouse model of asthma.^26^

**Fig 5:**
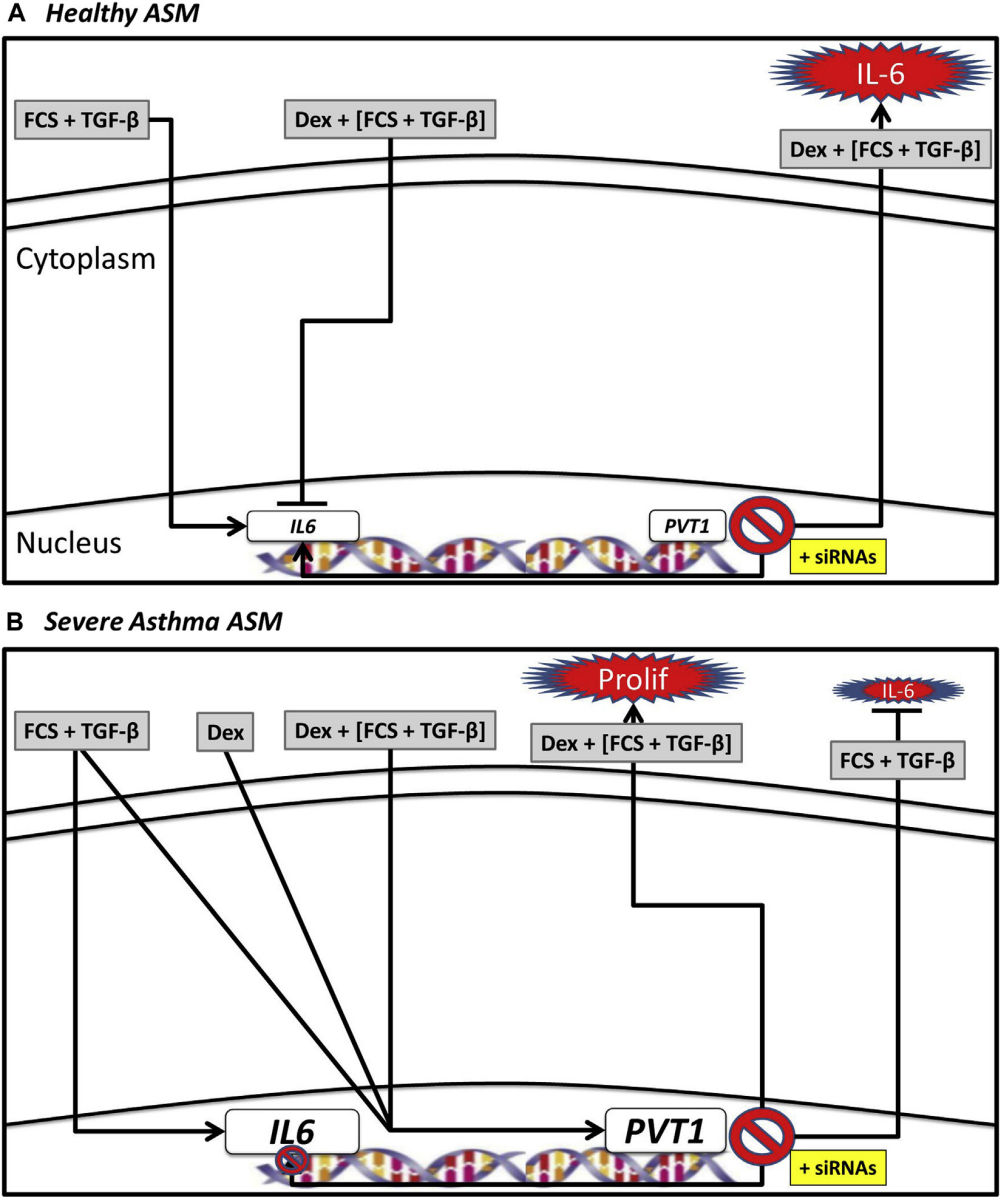
**A** and **B**, Potential mechanisms for *PVT1* contribution to ASMC proliferation and IL-6 release in asthmatic patients. *Dex*, Dexamethasone; *FCS*, fetal calf serum; *TGF-β*, transforming growth factor-beta.

We also measured the expression of lncRNAs in our patient cohorts. A large number of lncRNAs were differentially expressed under all situations (baseline, corticosteroid treatment, and mitogen stimulation). Interestingly, only *PVT1* was found to be decreased in ASMCs from patients with nonsevere asthma and increased in ASMCs from patients with severe asthma, and therefore we performed functional studies on this lncRNA in our model of asthmatic hyperproliferation and corticosteroid insensitivity.^3^, ^5^ The action of *PVT1* in our ASMCs from asthmatic patients was complex. As such, we have proposed a figure that shows potential mechanisms for *PVT1* in ASMC proliferation and IL-6 release (Fig 5). In ASMCs from nonasthmatic healthy subjects, TGF-β plus FCS induces *IL6* mRNA expression, which translates into IL-6 protein release. Interestingly, the addition of dexamethasone results in an increase in expression of *PVT1*, which, when inhibited by siRNAs (Fig 5, *A*), induces greater *IL6* mRNA expression and more IL-6 protein release. In ASMCs from patients with severe asthma, TGF-β plus FCS not only induces *IL6* mRNA expression and protein release but also *PVT1* expression. The effect of dexamethasone was the same, and when we inhibited *PVT1* in these ASMCs with siRNAs (Fig 5, *B*), a decrease in *IL6* mRNA and IL-6 protein release was observed, along with a concurrent increase in cellular proliferation. The mechanism of action of *PVT1* in human primary ASM is clearly complex. Very little is known about the role of *PVT1* in human pathologies. *PVT1* is frequently expressed in patients with numerous cancers (including lung)^27^, ^28^, ^29^, ^30^, ^31^ and diabetes.^32^, ^33^, ^34^ Furthermore, targeting of *PVT1* in these pathologies has been shown to have a range of effects, including decreasing proliferation and increasing apoptosis,^28^, ^35^ decreasing resistance to gemcitabine (a chemotherapy drug),^36^ and regulation of genes and proteins involved in ECM deposition.^32^ For the first time, we have demonstrated the novel way in which targeting *PVT1* in human primary ASMCs can affect phenotype.

The variable mechanism of action of *PVT1* could simply be a consequence of different cells, tissues, and pathologies examined; however, it might also be due to the action and configuration of *PVT1* itself. *PVT1* is a downstream target of *c-MYC* that targets and binds *PVT1*, driving its transcription.^17^ We show that in our severe asthmatic ASMC cohorts, dexamethasone increases *PVT1* expression, and inhibition of this results in an increase in FCS plus TGF-β–induced cellular proliferation, possibly by targeting of *c-MYC*.

Finally, *PVT1* is unlikely to be the only noncoding RNA (ncRNA) acting in our ASMC model. We have previously reported on the role of miRNAs in these cells after stimulation with TGF-β plus FCS,^3^ and now it is known that *PVT1* can express a cluster of 6 miRNAs, such as miR-1207-5p (which we discussed previously^4^). Although these ncRNAs are coexpressed, regulation of *miR-1207-5p* expression by TGF-β is independent of *PVT1*.^33^

In conclusion, we have demonstrated a large difference in ncRNA expression profiles (including miRNAs and lncRNAs) in primary human ASMCs from patients with nonsevere and severe asthma and the differential effects of adding corticosteroids and mitogen. Furthermore, we have found that *PVT1* regulates both IL-6 release and proliferation in ASMCs from patients with severe asthma.

**Key messages**

• Severe asthma is a worldwide health issue that is mostly unresponsive to existing therapy.
• lncRNAs are differentially expressed between ASMCs from patients with nonsevere and severe asthma.
• Targeting of the lncRNA *PVT1* can reduce increased cellular proliferation and IL-6 release from ASMCs in patients with severe asthma.

## Figures and Tables

**Table I:** Characteristics of subjects

	Nonasthmatic patients	Patients with nonsevere asthma	Patients with severe asthma
No.	9	9	9
Age (y)	36.4 ± 12.7	42.4 ± 16.2	48.9 ± 11
Sex (male/female)	7/2	6/4	3/6
Asthma duration (y)	NA	22.2 ± 16.8	25.6 ± 13.2
Inhaled corticosteroid dose (μg of BDP equivalent)	NA	580 ± 576.9	1688.9 ± 176.4
Atopy (no.)∗	0	8	8
Receiving oral corticosteroids (no.)	0	0	7
FEV_1_ (L)	4.02 ± 0.48	2.81 ± 0.71	2.7 ± 0.82
FEV_1_ (% predicted)	104.23 ± 7.28	84.68 ± 12.31	80.48 ± 12.34
FEV_1_/FVC ratio (%)	78.79 ± 5.98	69.87 ± 9.27	63.98 ± 9.68
β-Agonist reversibility (%)†	NA	12.3 ± 11.9	19.54 ± 14.56
PC_20_ (mg/mL)	>16	0.69 ± 0.64	0.2 ± 0.39

Data are shown as means ± SEMs.

*BDP*, Beclomethasone dipropionate; *FVC*, forced vital capacity; *NA*, not available.

∗ Defined as positive skin prick test responses to 1 or more common aeroallergens.

† Measured as percentage increase in FEV_1_ after 400 μg of salbutamol.

## Supplementary data

Online Repository text
