# Supplementary material for: Transcriptional profiling identifies the long noncoding RNA plasmacytoma variant translocation (PVT1) as a novel regulator of the asthmatic phenotype in human airway smooth muscle
Source: J Allergy Clin Immunol. 2017 Mar;139(3):780–9. doi: 10.1016/j.jaci.2016.06.014 (PMC5338875; doi:10.1016/j.jaci.2016.06.014)
Supplement: Online Repository text [file mmc1.docx]

# *Online Repository*

**Transcriptional profiling identifies the lncRNA *PVT1* as a novel regulator of the asthmatic phenotype in human airway smooth muscle**

Phillip J. Austin^1^, Eleni Tsitsiou^2^, Charlotte Boardman^1^, Mark A. Lindsay^1,2,3^, Ian M. Adcock^1^, Kian Fan Chung^1^ and Mark M. Perry^4^

***Methods***

**Subject selection**

Patients with non-severe and severe asthma as defined by European Respiratory Society/American Thoracic Society Workshop on Severe Asthma^(1)^ were recruited. Current smokers and former smokers with greater than 5 pack-year history were excluded. Non-asthmatic normal subjects with no previous history of asthma and a PC_20_ >16 mg/ml were also recruited. The subject characteristics are shown in **Table 1**. Each subject underwent a fiberoptic bronchoscopic study under sedation with midazolam and topical anaesthesia to the airways with lidocaine. Airway biopsies were taken from segmental and sub-segmental airways of the right lower lobe. This study was approved by the Ethics Committee and all subjects gave written informed consent.

**ASM cell culture and stimulation**

ASM cells were cultured as previously described^(2-6)^. Confluent cells were growth-arrested by FCS deprivation for 24 h in Dulbecco’s Modified Eagle’s Medium supplemented with sodium pyruvate (1 mM), L-glutamine (2 mM), non-essential amino acids (1:100), penicillin (100 U/ml)/streptomycin (100 mg/ml), amphotericin B (1.5 mg/ml), and BSA (0.1 %). Human airway smooth muscle cells at passages 3–4 from nine different donors were used. Cells were pre-treated with dexamethasone (10^-7^ M) for 1 h, before being stimulated with 2.5 % FCS for 24 h. The supernatants were removed and IL-6 levels were determined by DuoSet ELISA (R&D Systems, Abingdon, UK) as a measure of ASM cell activation (**Figure E1**). Cell proliferation was measured by the Cell Proliferation ELISA BrdU kit (Roche Applied Science), an assay comparable to cell counting as confirmed by flow cytometry^(2-5)^. Cellular viability was assessed by MTT assay^(7)^. Each patient sample was replicated over 3 wells.

**RNA extraction**

Total RNA was extracted using the mirVana™miRNA isolation kit (Ambion Europe). RNA was eluted in 50 μl RNase-free water (Promega UK, Southampton, UK) and stored at -70 °C. RNA content and purity was measured using a BioTek PowerWave XS (SSi Robotics, Tustin, CA, U.S.A.) spectrophotometer (yield (± SD): 1.1 ± 0.3 mg/ml; purity (A_260_/A_280_): 2.0 ± 0.1).

**Microarray Analysis**

LncRNA and mRNA expression was determined using the Agilent SurePrint G3 Human GE microarrays, as previously described^(3)^.

**Pathway Analysis**

Differentially expressed mRNAs from each dataset were further analysed using the bioinformatics software application "Ingenuity Pathway Analysis" application ([www.ingenuity.com](http://www.ingenuity.com)). A "Core Functional Analysis" was performed to identify canonical pathways, predicted upstream regulators and gene networks most significantly associated with the differentially expressed mRNAs.

The significance of the association of a given canonical pathway with the differentially expressed mRNAs was measured in two ways. Firstly, by the ratio of the number of differentially expressed mRNAs in the dataset that mapped to the canonical pathway divided by the total number of genes that map to the canonical pathway. Secondly, Fisher's exact test was used to calculate a P-value of the association between the mRNA and the network/canonical pathway.

**Quantitative PCR measurement of miRNA and mRNA expression**

miRNA expression was undertaken with the 2-step Applied Biosystems TaqMan RT-PCR protocol (Applied Biosystems) and normalized to 18S, as previously described^(2;3;8)^. mRNA expression was determined using TaqMan RT-PCR with Assays on Demand (Applied Biosystems). The separate-well 2^-(ΔΔCt)^ method was used to determine relative quantitative levels of individual miRNAs and mRNAs.

**Transfection with siRNAs that target *PVT1***

ASMCs were transfected as previously described^(2;9;10)^. siRNAs designed to target *PVT1* and *IL6* were purchased from Ambion/Applied Biosystems, Ltd. ASMCs were transfected with *PVT1* inhibitor (30, 100, 300 nM), *IL6* inhibitor (100 nM), or Silencer^®^ Negative Control #1 (100 nM) and no siRNA (mock transfection). Transfection conditions demonstrated no adverse effects upon the ASMCs (**Figure E2**), as previously demonstrated^(1-3)^.

**Data and statistical analysis**

Data were analysed using GraphPad Prism, version 5.03. Data were not normally distributed (as assessed by the Kolmogorov- Smirnov test), and therefore groups were compared using the Dunn nonparametric test. All data are expressed as means ± SEMs.

**Supplemental Table 1: Baseline mRNA expression in non-severe ASM compared to healthy ASM cells**

| **Gene** | **P value** | **FC** | **Gene** | **P value** | **FC** |
| --- | --- | --- | --- | --- | --- |
| ERCC8 | 0.00256679 | 8.12903 | GAL | 0.0394773 | 2.1095 |
| SLC16A6 | 0.0166426 | 4.65081 | AKR1B10 | 0.0449671 | 2.07792 |
| HMSD | 0.00663085 | 4.3893 | FITM2 | 0.0183232 | 2.07506 |
| HSD17B2 | 0.0443303 | 3.89864 | SYTL2 | 0.0198481 | 2.06856 |
| IGDCC4 | 0.0465114 | 3.6928 | FLYWCH2 | 0.0121742 | 2.06587 |
| MMP1 | 0.0259227 | 3.35243 | EVI2A | 0.0277221 | 2.06063 |
| MMP3 | 0.0304801 | 3.22102 | SLC7A14 | 0.0383132 | 2.03911 |
| TRPA1 | 0.01778 | 3.13945 | GNASAS | 0.0482069 | 2.03829 |
| SALL1 | 0.0052357 | 2.99666 | RAB30 | 0.0289699 | 2.03204 |
| RGS2 | 0.00555875 | 2.95017 | KIAA1217 | 0.0265248 | 2.01769 |
| FLI1 | 0.00364385 | 2.8631 | PTH1R | 0.0329649 | 1.97698 |
| HRK | 0.0429068 | 2.83682 | C4orf36 | 0.0290853 | 1.96317 |
| UCP2 | 0.00851212 | 2.79613 | EID2B | 0.00613632 | 1.96143 |
| RARRES2 | 0.020582 | 2.77563 | ARRDC4 | 0.0328818 | 1.95579 |
| CLEC2B | 0.00642905 | 2.77082 | PAX4 | 0.0409322 | 1.95439 |
| CARD17 | 0.00592314 | 2.70494 | KCNN2 | 0.0242211 | 1.95348 |
| TSHZ2 | 0.0134582 | 2.6941 | SNORA53 | 0.0220681 | 1.94978 |
| RNMT | 0.00515799 | 2.63754 | ARHGAP18 | 0.0106662 | 1.93914 |
| C20orf196 | 0.0479645 | 2.62389 | TBPL1 | 0.00781103 | 1.93791 |
| CARD16 | 0.00841454 | 2.61194 | TMEM97 | 0.00858623 | 1.93744 |
| C9orf110 | 0.043834 | 2.55355 | LRP8 | 0.0443823 | 1.93136 |
| C17orf60 | 0.0267141 | 2.5307 | SIPA1L3 | 0.00637892 | 1.91352 |
| ICAM4 | 0.0437101 | 2.40862 | NR_024569 | 0.0186056 | 1.90429 |
| HMGCS1 | 0.0424883 | 2.39058 | SAR1P3 | 0.00898055 | 1.89096 |
| CCDC68 | 0.0233077 | 2.37967 | C17orf86 | 0.00453316 | 1.88747 |
| COL10A1 | 0.0250919 | 2.35495 | CKS2 | 0.0142289 | 1.87502 |
| EPHX4 | 0.00462827 | 2.34974 | CDK17 | 0.020251 | 1.872 |
| CST2 | 0.0389638 | 2.34496 | C8orf41 | 0.00323954 | 1.856 |
| CDYL2 | 0.00955754 | 2.34042 | SEMA3A | 0.0369473 | 1.85269 |
| LCE2A | 0.0266531 | 2.32617 | COQ3 | 0.0370812 | 1.84123 |
| TNFSF4 | 0.0253868 | 2.31612 | GLRX | 0.0111158 | 1.82913 |
| IL7R | 0.0408885 | 2.2781 | GIT1 | 0.00646277 | 1.81225 |
| NES | 0.00362585 | 2.27808 | C13orf1 | 0.0333645 | 1.80632 |
| SC4MOL | 0.0481442 | 2.26589 | STMN3 | 0.0166473 | 1.80345 |
| CCDC102B | 0.0140359 | 2.21734 | SPRY1 | 0.032023 | 1.79181 |
| LOC100130938 | 0.00548399 | 2.20849 | FANCA | 0.00139521 | 1.78639 |
| ADAMTS9 | 0.0199445 | 2.20162 | PPP1R2P3 | 0.030498 | 1.78449 |
| TTC12 | 0.00392433 | 2.18397 | LOC728353 | 0.0144362 | 1.76044 |
| LYN | 0.0191528 | 2.17462 | FAIM3 | 0.0129136 | 1.75518 |
| LPPR3 | 0.0220692 | 2.17076 | COPG2 | 0.00572741 | 1.75405 |
| C13orf15 | 0.0234561 | 2.13801 | KIR3DL3 | 0.035418 | 1.74011 |
| SATB2 | 0.012104 | 2.13354 | ATG9B | 0.0388154 | 1.73542 |
| SNORA74B | 0.041715 | 2.12524 | LOC100128340 | 0.0329169 | 1.73146 |
| MRPS23 | 0.0117045 | 2.12179 | RIPK3 | 0.0234775 | 1.73035 |
| ATP2A2 | 0.0172857 | 2.11786 | TMC4 | 0.00187281 | 1.72712 |
| PTGES | 0.0464004 | 1.72193 | LPO | 0.0312946 | 1.51464 |
| SGIP1 | 0.0427581 | 1.71441 | C1orf97 | 0.0127892 | 1.51182 |
| C11orf85 | 0.022358 | 1.70651 | TCERG1 | 0.0210095 | 1.50193 |
| LY6G5B | 0.0323101 | 1.70556 | C11orf94 | 0.03963 | 1.50051 |
| PDCD1LG2 | 0.0460351 | 1.69217 | C12orf34 | 0.0134796 | -1.50109 |
| MOGAT2 | 0.0454009 | 1.6802 | OLFML2B | 0.00965737 | -1.50279 |
| ITIH1 | 0.0179354 | 1.67862 | GAA | 0.027051 | -1.5045 |
| DGCR11 | 0.0295952 | 1.67359 | SLC13A3 | 0.0227501 | -1.51245 |
| LOC374443 | 0.0248206 | 1.6646 | IQCE | 0.031671 | -1.51583 |
| DUSP18 | 0.0406348 | 1.65337 | SLIT3 | 0.0098095 | -1.51648 |
| ETV4 | 0.0257434 | 1.64981 | COL16A1 | 0.0296194 | -1.51924 |
| CD99 | 0.0252357 | 1.64791 | METRNL | 0.0290532 | -1.51963 |
| R3HDML | 0.0197006 | 1.64091 | CLDN15 | 0.0472718 | -1.53115 |
| APIP | 0.0293658 | 1.62597 | MUC4 | 0.0243439 | -1.5319 |
| ICAM5 | 0.0162558 | 1.62544 | LAMB2 | 0.027407 | -1.53783 |
| KLHL7 | 0.0397881 | 1.62432 | KCND3 | 0.017771 | -1.5382 |
| FBXO3 | 0.0496671 | 1.61443 | PBX3 | 0.00198743 | -1.53883 |
| TGDS | 0.00616609 | 1.61348 | RN18S1 | 0.0385365 | -1.54727 |
| C20orf135 | 0.0285209 | 1.61253 | SH3BP5 | 0.00623863 | -1.56086 |
| IL1RAPL1 | 0.00713879 | 1.61077 | CAND2 | 0.0156141 | -1.5663 |
| AX748082 | 0.0126975 | 1.60578 | SYNE2 | 0.0382512 | -1.59534 |
| CEPT1 | 0.040725 | 1.60157 | P2RY11 | 0.0449651 | -1.60603 |
| LRP11 | 0.0436053 | 1.59635 | CCDC19 | 0.0123329 | -1.6178 |
| WNT6 | 0.0235748 | 1.59304 | TGFB3 | 0.0278389 | -1.62348 |
| NAAA | 0.0289171 | 1.58906 | ZNF230 | 0.0114767 | -1.63745 |
| ASB8 | 0.027996 | 1.58609 | NPIPL3 | 0.0361165 | -1.63849 |
| ZNF222 | 0.0223744 | 1.58609 | KCNG1 | 0.031928 | -1.64202 |
| S100P | 0.0345147 | 1.58354 | FAT4 | 0.0394372 | -1.65585 |
| HTR7 | 0.0486079 | 1.58315 | GMDS | 0.010624 | -1.65937 |
| FAM176A | 0.0455432 | 1.58124 | PIP5KL1 | 0.0423751 | -1.6623 |
| ATL1 | 0.0229584 | 1.56837 | AQP3 | 0.0283477 | -1.6645 |
| ARSG | 0.0105803 | 1.5673 | FBXO32 | 0.0433856 | -1.6664 |
| ADAM9 | 0.0375495 | 1.55888 | DIRAS1 | 0.038796 | -1.66986 |
| LOC442572 | 0.0258556 | 1.5564 | GYPA | 0.0230486 | -1.68431 |
| ZNF789 | 0.0282785 | 1.5487 | GLIS2 | 0.0127594 | -1.68923 |
| LIPT1 | 0.0292182 | 1.54776 | PBX1 | 0.00848981 | -1.69002 |
| ASL | 0.0349748 | 1.5466 | ATP1B1 | 0.045649 | -1.69544 |
| GALNT4 | 0.00944573 | 1.54382 | DMD | 0.0497104 | -1.69705 |
| MGC16703 | 0.0079992 | 1.54334 | NEK10 | 0.0329041 | -1.6991 |
| VWA2 | 0.00431907 | 1.54086 | TLX2 | 0.00613515 | -1.70613 |
| TATDN1 | 0.0400969 | 1.53946 | CCDC80 | 0.0452328 | -1.71459 |
| CNR2 | 0.0366879 | 1.53872 | NPR1 | 0.00816906 | -1.71749 |
| CASS4 | 0.0270782 | 1.52985 | NNAT | 0.0314012 | -1.72845 |
| OSGIN1 | 0.0088119 | 1.52734 | GHR | 0.0306231 | -1.73155 |
| ZNF195 | 0.00969063 | 1.52537 | NAV2 | 0.0113647 | -1.73644 |
| OSTF1 | 0.0349153 | 1.52403 | CACNB2 | 0.0467284 | -1.74416 |
| ZNF417 | 0.0383173 | 1.52402 | ZNF503 | 0.00907856 | -1.74658 |
| RAD51 | 0.0388823 | 1.52261 | C9orf96 | 0.0105939 | -1.74742 |
| S100A3 | 0.0113228 | 1.51853 | DACT1 | 0.023388 | -1.75241 |
| ZNF107 | 0.0215264 | -1.76592 | ACCN1 | 0.0331635 | -2.10818 |
| GKAP1 | 0.0490861 | -1.76878 | C5orf39 | 0.0450539 | -2.11464 |
| SNAR-G1 | 0.0383436 | -1.77303 | PRKCH | 0.00336745 | -2.13392 |
| SHANK2 | 0.048114 | -1.783 | TMEM151B | 0.0380533 | -2.13457 |
| DOK5 | 0.0136432 | -1.78756 | CRL2 | 0.00269115 | -2.16473 |
| SEMA3F | 0.0276809 | -1.79524 | ANGPTL1 | 0.0236193 | -2.18693 |
| PGM5 | 0.00393577 | -1.80463 | CMYA5 | 0.000900832 | -2.19022 |
| PEG10 | 0.0233929 | -1.81285 | PPL | 0.0122776 | -2.19369 |
| OR2A9P | 0.0494225 | -1.81684 | KIAA1644 | 0.0292289 | -2.21229 |
| LOC388242 | 0.00329465 | -1.81988 | NOVA1 | 0.0146329 | -2.21791 |
| REP15 | 0.00864225 | -1.8237 | STAC | 0.0195427 | -2.23498 |
| PDE8B | 0.035244 | -1.82845 | KCNJ8 | 0.00643319 | -2.28672 |
| HTRA1 | 0.0112554 | -1.83021 | PDLIM4 | 0.0041358 | -2.29975 |
| CCDC151 | 0.00528945 | -1.832 | NR3C2 | 0.0201973 | -2.30728 |
| HLX | 0.0257786 | -1.8349 | KLHDC7B | 0.00447221 | -2.31489 |
| SPTLC3 | 0.0129416 | -1.83967 | C10orf11 | 0.0397767 | -2.32799 |
| BEST4 | 0.0010698 | -1.84151 | SLC39A5 | 0.01412 | -2.3978 |
| NFIA | 0.0218309 | -1.87978 | CYP2J2 | 0.0314292 | -2.4092 |
| SLC6A9 | 0.0234398 | -1.8842 | GALNT12 | 0.00283954 | -2.445 |
| FNBP1L | 0.0286074 | -1.88962 | GDF10 | 0.0227301 | -2.45552 |
| MFAP4 | 0.0489281 | -1.9139 | EPHA5 | 0.00717682 | -2.54064 |
| L2HGDH | 0.00913428 | -1.9146 | TNNT2 | 0.0353226 | -2.54526 |
| CLU | 0.00740704 | -1.92507 | CGNL1 | 0.023339 | -2.56621 |
| NLGN1 | 0.0099885 | -1.92683 | C10orf10 | 0.0327907 | -2.57199 |
| TMEM59L | 0.00108387 | -1.93338 | IGF1 | 0.0103895 | -2.58664 |
| DIRC3 | 0.0470113 | -1.93538 | NRCAM | 0.00254471 | -2.60154 |
| MATN2 | 0.0142377 | -1.93694 | DPT | 0.00643853 | -2.60339 |
| ABCC9 | 0.0358009 | -1.94478 | MFAP5 | 0.044958 | -2.61452 |
| ZFPM2 | 0.0194637 | -1.94683 | SULF1 | 0.00534576 | -2.66216 |
| TMOD4 | 0.00319358 | -1.95017 | COL14A1 | 0.000357204 | -2.66577 |
| ABLIM1 | 0.00177999 | -1.96815 | KIAA1324L | 0.0359039 | -2.67384 |
| FUCA1 | 0.0405767 | -1.97474 | DHRS3 | 0.00774106 | -2.75273 |
| SLIT3 | 0.0398184 | -1.98453 | FGF18 | 0.000896677 | -2.80081 |
| VWCE | 0.0312087 | -1.98542 | JAM2 | 0.00305768 | -2.80562 |
| FGF13 | 0.0138649 | -1.99866 | GPER | 0.000233449 | -2.84791 |
| LAMA3 | 0.0341782 | -2.00515 | COL6A6 | 0.0472692 | -2.92356 |
| OR10G3 | 0.00739 | -2.01027 | EPHB6 | 0.0216317 | -2.92423 |
| TACSTD2 | 0.0488225 | -2.01974 | CHRDL1 | 0.012315 | -2.92678 |
| SCN2A | 0.00858522 | -2.02318 | GPR68 | 0.0122055 | -3.02474 |
| C8orf46 | 0.00669657 | -2.03351 | FLJ41603 | 0.00358055 | -3.02479 |
| ITIH3 | 0.0435118 | -2.03558 | PTGIS | 0.0300997 | -3.05012 |
| ADAMTS3 | 0.00983634 | -2.03924 | GNAZ | 0.031785 | -3.07734 |
| HTRA3 | 0.0243531 | -2.04246 | C7orf69 | 0.0411363 | -3.09401 |
| SERPINA3 | 0.0225425 | -2.05235 | NEFM | 0.00778604 | -3.13218 |
| LIF | 0.00101274 | -2.06048 | FNDC1 | 0.0119883 | -3.2049 |
| SLC4A4 | 0.0246772 | -2.08013 | OGN | 0.00233206 | -3.27812 |
| FOXC1 | 0.015423 | -2.08185 | SCN4B | 0.0159437 | -3.38216 |
| COBLL1 | 0.0270806 | -2.10497 | C13orf33 | 0.0265376 | -3.4087 |
| AKAP12 | 0.0137054 | -2.10649 | TMEM30B | 0.00673075 | -3.61571 |
| CRLF1 | 0.00736869 | -3.6385 | PTPRD | 0.00132004 | -4.4638 |
| AGT | 0.020381 | -3.64996 | TMEFF2 | 8.25E-06 | -4.54125 |
| CCDC3 | 0.0117285 | -3.7839 | PI16 | 0.0304839 | -4.90266 |
| CXCR7 | 0.0178151 | -3.94469 | NFIB | 0.0111192 | -4.94463 |
| MGP | 0.000631693 | -3.97238 | SCN2B | 0.000479454 | -7.85751 |
| CCKAR | 0.00487403 | -4.32258 |  |  |  |

**Supplemental Table 2: Baseline mRNA expression in severe ASM compared to healthy ASM cells**

| **Gene** | **P value** | **FC** | **Gene** | **P value** | **FC** |
| --- | --- | --- | --- | --- | --- |
| CHI3L1 | 0.00322219 | 6.61982 | SYTL2 | 0.0328305 | 1.96693 |
| TRIM63 | 0.0231446 | 4.09239 | ENTPD7 | 0.0257107 | 1.96311 |
| APBA2 | 0.0445882 | 3.82477 | LPPR3 | 0.0489578 | 1.9601 |
| IL34 | 0.0191848 | 3.69834 | COQ3 | 0.026979 | 1.95358 |
| CDC73 | 0.0269521 | 3.68601 | CES1 | 0.0362807 | 1.89267 |
| HRK | 0.0211963 | 3.44723 | EFNB1 | 0.0338861 | 1.87755 |
| KIAA1199 | 0.0148091 | 3.38659 | INA | 0.019085 | 1.81494 |
| LRRC15 | 0.0295012 | 3.24591 | FLJ31715 | 0.0352514 | 1.81384 |
| WISP2 | 0.0258918 | 3.08252 | GGT5 | 0.0384159 | 1.79639 |
| HLA-DRB3 | 0.0241551 | 3.0408 | UPF0621 | 0.0193493 | 1.79492 |
| PLA2G5 | 0.0270857 | 3.02936 | LOC390251 | 0.0129469 | 1.76937 |
| FCRL1 | 0.0229921 | 2.81958 | B3GALTL | 0.0357022 | 1.76502 |
| BOC | 0.0496743 | 2.69953 | PLA2G16 | 0.0429882 | 1.76186 |
| RANBP2 | 0.0213297 | 2.67464 | ST3GAL5 | 0.0351087 | 1.7441 |
| KCND2 | 0.0474138 | 2.63907 | ZNF91 | 0.0336617 | 1.73603 |
| SERPINB9 | 0.0267262 | 2.36852 | CDK17 | 0.0436539 | 1.73378 |
| AQP11 | 0.00276018 | 2.32601 | PIGZ | 0.0496192 | 1.72694 |
| ASB2 | 0.0247058 | 2.25971 | SERPINE2 | 0.0453189 | 1.72255 |
| CCL28 | 0.0294344 | 2.18907 | MT1G | 0.0347974 | 1.71768 |
| P2RX7 | 0.0176809 | 2.18229 | POLR2F | 0.0103218 | 1.69222 |
| LOC649941 | 0.00266206 | 2.13469 | C4orf22 | 0.0364316 | 1.69198 |
| PLXDC2 | 0.0285438 | 2.07502 | TRIM78P | 0.0377634 | 1.68077 |
| SCUBE3 | 0.00809742 | 2.06145 | GNPTAB | 0.00505003 | 1.67564 |
| SLC30A3 | 0.0191482 | 2.05979 | ELOVL4 | 0.0384621 | 1.67198 |
| LOC645431 | 0.00327623 | 2.04048 | MID2 | 0.0272579 | 1.66531 |
| PLXNC1 | 0.03469 | 2.00889 | MYO1E | 0.00629935 | 1.64458 |
| TSKS | 0.0404227 | 2.00233 | DA992326 | 0.0232459 | 1.63417 |
| ARRDC4 | 0.0322396 | 1.99718 | FAM179A | 0.016525 | 1.62278 |
| SBSN | 0.0333072 | 1.98322 | ARL4C | 0.0299183 | 1.61076 |
| GPRC5C | 0.00819651 | 1.97791 | RGNEF | 0.0401281 | 1.60602 |
| FZD8 | 0.0445338 | 1.97657 | AIMP1 | 0.0260581 | 1.59682 |
| OR6N1 | 0.0459387 | 1.97118 | ZNF850P | 0.0490557 | 1.59094 |
| GPC3 | 0.0416173 | 1.5907 | TMOD4 | 0.0126156 | -1.75445 |
| CU678159 | 0.0435196 | 1.58094 | FHL3 | 0.00232316 | -1.76187 |
| MAF | 0.0488807 | 1.5683 | KCNMB4 | 0.044772 | -1.76424 |
| PCOTH | 0.0189505 | 1.56159 | NLGN1 | 0.0257432 | -1.76981 |
| FAM107B | 0.0134004 | 1.5443 | LOC100128340 | 0.0273873 | -1.79563 |
| RASGRP4 | 0.0091248 | 1.53014 | TMSB15A | 0.0472232 | -1.80402 |
| ZC3H12C | 0.0366664 | 1.52823 | G6PD | 0.0143391 | -1.80527 |
| ALX3 | 0.01248 | 1.52661 | PGA3 | 0.029713 | -1.82591 |
| EPB42 | 0.0404321 | 1.52063 | GSTM2 | 0.00624907 | -1.83722 |
| SOAT1 | 0.0465077 | 1.51478 | RTF1 | 0.0342033 | -1.83891 |
| ZNF417 | 0.0468942 | 1.51189 | MIR155HG | 0.0463894 | -1.84051 |
| SUSD2 | 0.0267075 | 1.50014 | EDIL3 | 0.00907355 | -1.846 |
| EID1 | 0.0372519 | 1.5 | FOXC1 | 0.0436204 | -1.84634 |
| RXRB | 0.0192761 | -1.51577 | BC013171 | 0.0176268 | -1.85591 |
| COL9A3 | 0.0275545 | -1.52281 | LEMD2 | 0.0283376 | -1.87251 |
| THC2551759 | 0.025643 | -1.5293 | TEKT4 | 0.0253215 | -1.87611 |
| LIF | 0.0360369 | -1.54658 | MATN2 | 0.0206684 | -1.88823 |
| DNAJC4 | 0.0231423 | -1.55047 | GLIS1 | 0.0160045 | -1.90408 |
| LOC100129721 | 0.0389218 | -1.55148 | UCN2 | 0.00691849 | -1.93071 |
| PARVB | 0.0485998 | -1.56027 | NOVA1 | 0.041179 | -1.95198 |
| KIAA1683 | 0.030854 | -1.57456 | HBA2 | 0.00836222 | -1.9605 |
| OLFML3 | 0.0229235 | -1.58161 | EDNRA | 0.00849789 | -1.98684 |
| LOC606724 | 0.0305021 | -1.58414 | HCLS1 | 0.0126303 | -2.01169 |
| CMYA5 | 0.0382822 | -1.58529 | CCDC36 | 0.0361258 | -2.03234 |
| AF495723 | 0.0197298 | -1.5889 | COL7A1 | 0.00806561 | -2.03399 |
| PIWIL4 | 0.00386534 | -1.59655 | C8orf46 | 0.00705425 | -2.06141 |
| FES | 0.013501 | -1.59932 | DPT | 0.0325449 | -2.10323 |
| AK124041 | 0.0098982 | -1.6135 | TMEM151B | 0.0410405 | -2.15069 |
| HSD11B2 | 0.0361224 | -1.61447 | PEG10 | 0.00432019 | -2.2238 |
| LOC100130557 | 0.0223642 | -1.63702 | EPHA5 | 0.0167505 | -2.30662 |
| BHLHE23 | 0.0374452 | -1.642 | CCDC85A | 0.0364354 | -2.32329 |
| DCHS1 | 0.0245409 | -1.6444 | ZNF462 | 0.0207553 | -2.35787 |
| SH3GL1P3 | 0.00380501 | -1.64995 | WBSCR27 | 0.0236313 | -2.39805 |
| COL14A1 | 0.0445964 | -1.65881 | KIAA0430 | 0.037571 | -2.52596 |
| CYBA | 0.0107948 | -1.65884 | FLRT3 | 0.0499926 | -2.55809 |
| BAT2 | 0.0467807 | -1.67443 | KIAA1324L | 0.0370802 | -2.72654 |
| PIK3C2B | 0.0268458 | -1.71271 | FGF13 | 0.000851033 | -2.79977 |
| LGI4 | 0.027596 | -1.71907 | POMC | 0.0269916 | -2.89054 |
| PRR16 | 0.0161184 | -1.73578 | PSKH1 | 0.0218528 | -2.97571 |
| LOC100130713 | 0.0158694 | -1.73869 | BCHE | 0.0043359 | -3.02392 |
| RAX2 | 0.0264997 | -1.74198 | TWF2 | 0.0336147 | -3.03413 |
| PTPRD | 0.0128931 | -3.08855 | ACBD6 | 0.0498267 | -3.4529 |
| TMEFF2 | 0.000287566 | -3.16613 | DCTN3 | 0.0201069 | -3.82185 |
| RNF216L | 0.0428454 | -3.20267 | CCKAR | 0.00317157 | -4.91171 |
| NUDT22 | 0.0286962 | -3.40754 | CADM1 | 0.00090556 | -5.16425 |

**Supplemental Table 3: Common baseline mRNA expression changes in ASM cells from patients with non-severe or severe asthma vs. healthy**

|  |  | Non-Severe Asthma | | Severe Asthma |
| --- | --- | --- | --- | --- |
| Gene Symbol | **Gene Name** | **Microarray (FC)** | **Microarray (FC)** | |
| ARRDC4 | Arrestin domain containing 4 | 2.0 (p < 0.01) | 2.0 (p < 0.01) | |
| C8orf46 | Chromosome 8 open reading frame 46 | -2.0 (p < 0.01) | -2.1 (p < 0.01) | |
| CCKAR | Cholecystokinin A receptor | -4.3 (p < 0.01) | -4.9 (p < 0.01) | |
| CDK17 | Cyclin-dependent kinase 17 | 1.9 (p < 0.01) | 1.7 (p < 0.01) | |
| CMYA5 | Cardiomyopathy associated 5 | -2.2 (p < 0.01) | -1.6 (p < 0.01) | |
| COL14A1 | Collagen, type XIV, alpha 1 | -2.7 (p < 0.01) | -1.7 (p < 0.01) | |
| COPG2 | Coatomer protein complex, subunit γ-2 | 1.8 (p < 0.01) | 1.5 (p < 0.01) | |
| COQ3 | Coenzyme Q3 homolog | 1.8 (p < 0.01) | 2.0 (p < 0.01) | |
| DPT | Dermatopontin | -2.6 (p < 0.01) | -2.1 (p < 0.01) | |
| EPHA5 | EPH receptor A5 | -2.5 (p < 0.01) | -2.3 (p < 0.01) | |
| FGF13 | Fibroblast growth factor 13 | -2.0 (p < 0.01) | -2.8 (p < 0.01) | |
| FOXC1 | Forkhead box C1 | -2.1 (p < 0.01) | -1.8 (p < 0.01) | |
| HRK | Harakiri, BCL2 interacting protein | 2.8 (p < 0.01) | 3.4 (p < 0.01) | |
| KIAA1324L | Estrogen-induced gene 121-like protein | -2.7 (p < 0.01) | -2.7 (p < 0.01) | |
| LIF | Leukemia inhibitory factor | -2.1 (p < 0.01) | -1.5 (p < 0.01) | |
| LPPR3 | Lipid phosphate protein type 3 | 2.2 (p < 0.01) | 2.0 (p < 0.01) | |
| MATN2 | Matrilin 2 | -1.9 (p < 0.01) | -1.9 (p < 0.01) | |
| NLGN1 | Neuroligin 1 | -1.9 (p < 0.01) | -1.8 (p < 0.01) | |
| NOVA1 | Neuro-oncological ventral antigen 1 | -2.2 (p < 0.01) | -2.0 (p < 0.01) | |
| PBX3 | Pre-B-cell leukemia homeobox 3 | -1.5 (p < 0.01) | -1.4 (p < 0.01) | |
| PEG10 | Paternally expressed 10 | -1.8 (p < 0.01) | -2.2 (p < 0.01) | |
| PTPRD | Protein tyrosine phosphatase, receptor type, D | -4.5 (p < 0.01) | -3.1 (p < 0.01) | |
| SYTL2 | Synaptotagmin-like 2 | 2.1 (p < 0.01) | 2.0 (p < 0.01) | |
| TMEFF2 | Transmembrane protein with EGF-like and two follistatin-like domains 2 | -4.5 (p < 0.01) | -3.2 (p < 0.01) | |
| TMEM151B | Transmembrane protein 151B | -2.1 (p < 0.01) | -2.2 (p < 0.01) | |
| TMOD4 | Tropomodulin 4 (muscle) | -2.0 (p < 0.01) | -1.8 (p < 0.01) | |
| ZNF417 | Zinc finger protein 417 | 1.5 (p < 0.01) | 1.5 (p < 0.01) | |

**Supplemental Table 4: mRNAs in non-severe ASM changed in expression following stimulation with FCS (2.5 %)**

| **Gene** | **P value** | **FC** | **Gene** | **P value** | **FC** |
| --- | --- | --- | --- | --- | --- |
| HIST1H2AL | 0.042169 | 15.2972 | LOC644662 | 0.0433908 | 3.00074 |
| AFP | 0.045175 | 13.6111 | RNFT2 | 0.0460387 | 2.99992 |
| KIF2C | 0.0179581 | 12.5638 | GAPDHP32 | 0.0409119 | 2.98881 |
| TACC3 | 0.00420819 | 10.3329 | LHFPL4 | 0.0125018 | 2.96321 |
| HIST1H2BE | 0.0237102 | 9.36812 | C14orf145 | 0.0256065 | 2.95993 |
| UBE2C | 0.0492036 | 8.21674 | CLN6 | 0.00278737 | 2.95722 |
| KRTAP1-5 | 0.0103114 | 8.12693 | ASF1B | 0.0246229 | 2.92533 |
| CENPL | 0.0417908 | 7.93986 | MTHFD2 | 0.0292476 | 2.92091 |
| ACTG2 | 0.0226104 | 7.84917 | CECR3 | 0.00506878 | 2.88227 |
| FAM64A | 0.0385425 | 6.96685 | PPIL5 | 0.0235481 | 2.83299 |
| CENPF | 0.0256589 | 6.85437 | GINS4 | 0.0189066 | 2.78582 |
| GINS1 | 0.0236215 | 6.52347 | PPP1R12B | 0.0369438 | 2.76528 |
| CENPM | 0.046922 | 5.98313 | MS4A18 | 0.0105 | 2.71174 |
| DGCR14 | 0.0340715 | 5.74079 | DONSON | 0.00280379 | 2.62237 |
| DTL | 0.0424873 | 5.71324 | PPIH | 0.0486878 | 2.54108 |
| PKMYT1 | 0.0467408 | 5.32092 | NEK10 | 0.0372871 | 2.53094 |
| PSG5 | 0.0411558 | 5.0389 | TRMT11 | 0.0143645 | 2.52293 |
| NRP1 | 0.0207293 | 4.99781 | DARS2 | 0.0384515 | 2.52127 |
| PSG3 | 0.0430847 | 4.96387 | PPPDE2 | 0.0226035 | 2.5107 |
| SLC7A5 | 0.0168647 | 4.91765 | CLEC4A | 0.0271478 | 2.50964 |
| RP11-717D12 | 0.0125387 | 4.74994 | UHRF1 | 0.0114807 | 2.5059 |
| SNORA62 | 0.0480651 | 4.53985 | FAM183A | 0.0115952 | 2.50458 |
| BLM | 0.0359356 | 4.47101 | OSGEPL1 | 0.0055552 | 2.50087 |
| DHFR | 0.0362396 | 4.38697 | MCM8 | 0.0046727 | 2.48865 |
| TPX2 | 0.0236064 | 4.36345 | ZNF761 | 0.0124803 | 2.48471 |
| RP11-631M21.2 | 0.0391902 | 4.32804 | CD99 | 0.0476624 | 2.48135 |
| PSAT1 | 0.00586812 | 4.21463 | PSMD9 | 0.0224133 | 2.44675 |
| C6orf173 | 0.0116616 | 4.19215 | SFRS7 | 0.0198257 | 2.44667 |
| C12orf48 | 0.0150602 | 4.152 | C2orf63 | 0.00306824 | 2.4222 |
| COMP | 0.0417089 | 4.04159 | C22orf23 | 0.00530128 | 2.41289 |
| FGD2 | 0.00851702 | 4.00545 | MCOLN2 | 0.0355785 | 2.39538 |
| HIST1H3G | 0.0346906 | 3.91419 | IFI44L | 0.00670638 | 2.39198 |
| E2F2 | 0.0170241 | 3.75432 | MAGOHB | 0.0143099 | 2.36689 |
| C7orf69 | 0.0423192 | 3.69353 | SLC25A19 | 0.0401612 | 2.32236 |
| DNMT3B | 0.0019108 | 3.60621 | CALML4 | 0.020143 | 2.31044 |
| HPVC1 | 0.00500671 | 3.57815 | C1orf135 | 0.000107269 | 2.30686 |
| POLQ | 0.00972538 | 3.53489 | AK310642 | 0.0129591 | 2.29593 |
| SLC4A4 | 0.0457484 | 3.46774 | SFRS1 | 0.00309224 | 2.2717 |
| MPP2 | 0.0203948 | 3.46566 | THC2698732 | 0.014244 | 2.26837 |
| SLC12A8 | 0.0148354 | 3.41566 | MCCC2 | 0.0239829 | 2.25976 |
| CR979835 | 0.0119406 | 3.35836 | C3orf26 | 0.000136262 | 2.24602 |
| PSRC1 | 0.0319164 | 3.26343 | M27336 | 0.00519858 | 2.23891 |
| SEMA3C | 0.0277254 | 3.23105 | Uncharacterized | 0.0413966 | 2.23347 |
| LOC100127904 | 0.0108389 | 3.19899 | KIAA0467 | 0.0471104 | 2.19476 |
| HIST1H3E | 0.00995016 | 3.14865 | HN1L | 0.0115622 | 2.19265 |
| ZNF286A | 0.0106128 | 3.09122 | C1orf88 | 0.00356268 | 2.19033 |
| EID3 | 0.0158955 | 3.07464 | FITM2 | 0.0276101 | 2.14598 |
| AX747988 | 0.0100492 | 3.02907 | TRIM61 | 0.0214664 | 2.1356 |
| TLR3 | 0.0109683 | 2.13293 | TRPC4 | 0.000482049 | 1.71844 |
| FBF1 | 0.00359897 | 2.13183 | FAM163B | 0.0238488 | 1.71416 |
| KPNA2 | 0.0287595 | 2.11384 | HIF1A | 0.0175851 | 1.71293 |
| TOMM34 | 0.047636 | 2.10906 | LOC401357 | 0.0271595 | 1.70815 |
| PTPLA | 0.0216557 | 2.10699 | RXFP3 | 0.0308157 | 1.701 |
| SLC7A1 | 0.00855046 | 2.10298 | FLJ43315 | 0.00189219 | 1.6953 |
| ZNF846 | 0.0485604 | 2.10036 | ANKRD23 | 0.0327944 | 1.68543 |
| RNF170 | 0.0324877 | 2.09984 | RG9MTD2 | 0.0420216 | 1.68441 |
| RAD51 | 0.010671 | 2.05954 | EME1 | 0.0208152 | 1.68283 |
| IPMK | 0.0462493 | 2.05872 | ZNF773 | 0.0178852 | 1.67989 |
| DAZAP1 | 0.00609129 | 2.04506 | PPT1 | 0.0113626 | 1.67657 |
| AC009065.1 | 0.0220591 | 2.0435 | MTDH | 0.0378097 | 1.67045 |
| DHODH | 0.0229538 | 2.02404 | TARS | 0.0489756 | 1.66668 |
| MRPL52 | 0.0174852 | 2.00324 | LZIC | 0.0228597 | 1.66613 |
| CLDN15 | 0.00391595 | 2.00082 | ILKAP | 0.0466723 | 1.65865 |
| CBS | 0.0320916 | 1.98625 | HUWE1 | 0.0393995 | 1.65522 |
| DAD1L | 0.0467537 | 1.98429 | DHX32 | 0.0290673 | 1.65436 |
| TNC | 0.043421 | 1.978 | PSMD1 | 0.0448396 | 1.65114 |
| C17orf75 | 0.0345164 | 1.96439 | C8orf12 | 0.046566 | 1.65041 |
| TSR1 | 0.016276 | 1.94851 | C14orf106 | 0.0374258 | 1.64948 |
| TIPIN | 0.0119014 | 1.93712 | LOC497257 | 0.0126151 | 1.64176 |
| IGHV4 | 0.0498597 | 1.9338 | CASD1 | 0.0389019 | 1.63331 |
| RRP15 | 0.010714 | 1.93131 | HIST2H4B | 0.0278259 | 1.62088 |
| EIF4E | 0.0256744 | 1.93005 | ADAMTS2 | 0.0346808 | 1.62082 |
| LRRC23 | 0.0336998 | 1.92391 | PGAM5 | 0.00983565 | 1.61832 |
| ANKFY1 | 0.00990802 | 1.9159 | AB305862 | 0.00236905 | 1.61206 |
| NSFP1 | 0.0200841 | 1.90634 | RACGAP1P | 0.00628056 | 1.60766 |
| ZNF232 | 0.0331495 | 1.89857 | DHX34 | 0.0489499 | 1.60645 |
| MARS | 0.0195536 | 1.89063 | KBTBD2 | 0.035033 | 1.60309 |
| LOC388796 | 0.0490232 | 1.8668 | PIGA | 0.0329098 | 1.59928 |
| GSTCD | 0.0129876 | 1.86522 | ZNF259P1 | 0.0285483 | 1.59487 |
| DDIT4L | 0.0224462 | 1.86291 | CCDC75 | 0.0261684 | 1.58904 |
| ATP10A | 0.00458931 | 1.85362 | XPO1 | 0.0481318 | 1.58287 |
| USP49 | 0.00316534 | 1.85184 | RAGE | 0.00904688 | 1.57975 |
| PMPCA | 0.0361554 | 1.84998 | REP15 | 0.036773 | 1.57611 |
| TUBB | 0.00098612 | 1.83791 | MGC14436 | 0.0452944 | 1.57506 |
| CACYBP | 0.0116392 | 1.83219 | FUS | 0.022933 | 1.56518 |
| EPS15 | 0.0139386 | 1.81679 | ALKBH8 | 0.0186885 | 1.56504 |
| DPP3 | 0.0152883 | 1.81394 | IFRD2 | 0.0338035 | 1.55328 |
| LOC100132774 | 0.0251185 | 1.80329 | PHTF1 | 0.0213646 | 1.55108 |
| MKNK2 | 0.00159673 | 1.78837 | FH | 0.0428265 | 1.55003 |
| DLD | 0.00708761 | 1.78761 | SMN2 | 0.00451399 | 1.54349 |
| KIAA1310 | 0.0309488 | 1.78021 | SIL1 | 0.0137999 | 1.54321 |
| AIFM2 | 0.0486621 | 1.77614 | SLC19A3 | 0.0429533 | 1.53829 |
| PLA2G16 | 0.0145217 | 1.77477 | SFRS3 | 0.0114201 | 1.53351 |
| ATP6V1C2 | 0.0241497 | 1.76747 | RNF8 | 0.044035 | 1.53324 |
| FASTKD2 | 0.0189688 | 1.76226 | CDC10L | 0.0161337 | 1.53244 |
| FLVCR1 | 0.0244468 | 1.7581 | SDS | 0.0497296 | 1.52589 |
| KLHL26 | 0.0095282 | 1.75207 | GLRX2 | 0.0337985 | 1.52024 |
| BLZF1 | 0.0139667 | 1.74802 | C2orf86 | 0.030794 | 1.50989 |
| OGFOD1 | 0.0492616 | 1.74646 | LRP11 | 0.0327101 | 1.50692 |
| TGM2 | 0.00809875 | 1.72013 | TTC39B | 0.0304907 | 1.50559 |
| PAN3 | 0.0261818 | 1.50394 | LOC283335 | 0.0407034 | -1.93147 |
| LOC100130093 | 0.00400245 | -1.50118 | TMEM158 | 0.0307114 | -1.94396 |
| SLC35B4 | 0.0420702 | -1.50184 | FLJ10357 | 0.0264898 | -1.95443 |
| SPINK7 | 0.00238846 | -1.50987 | TCF7L1 | 0.0408114 | -1.95539 |
| NFKBIE | 0.00217181 | -1.51769 | SLC37A2 | 0.0245677 | -1.97465 |
| SIX4 | 0.0455931 | -1.52335 | PDZD2 | 0.036989 | -1.98523 |
| BTNL8 | 0.0479463 | -1.52593 | BC038245 | 0.00630883 | -1.99291 |
| APBA2 | 0.00966014 | -1.53101 | CDKN2B | 0.0371591 | -2.00957 |
| TMTC4 | 0.0431429 | -1.53995 | IGKV2D | 0.00469022 | -2.01651 |
| PIP5K1C | 0.0483843 | -1.54103 | BAI1 | 0.0245881 | -2.05008 |
| COL8A1 | 0.0391784 | -1.5436 | TLR2 | 0.0304678 | -2.05671 |
| KLF3 | 0.0337641 | -1.54606 | MAF | 0.0182159 | -2.05987 |
| AK097103 | 0.0211365 | -1.55156 | IGDCC4 | 0.0250946 | -2.10885 |
| C1QL1 | 0.0124643 | -1.55905 | CCL8 | 0.0410618 | -2.11255 |
| USP7 | 8.85E-05 | -1.56233 | C3orf17 | 0.00177469 | -2.16793 |
| B3GNT8 | 0.0184764 | -1.57443 | SEZ6L2 | 0.00263898 | -2.20901 |
| GAL3ST4 | 0.0323236 | -1.58089 | TMEM100 | 0.023037 | -2.22181 |
| LOC100131831 | 0.018233 | -1.5968 | UACA | 0.0244656 | -2.24136 |
| EGR1 | 0.0459262 | -1.6054 | CCDC102B | 0.0363297 | -2.28346 |
| C21orf7 | 0.0280334 | -1.60858 | EMILIN2 | 0.0240823 | -2.32076 |
| EVC2 | 0.0240317 | -1.61969 | C14orf138 | 0.0412158 | -2.33047 |
| GLUL | 0.010041 | -1.62454 | ISYNA1 | 0.0384217 | -2.33646 |
| GPC2 | 0.0302108 | -1.62894 | ARHGAP26 | 0.0437545 | -2.34708 |
| CYBRD1 | 0.0271256 | -1.63724 | CCDC68 | 0.0399964 | -2.34718 |
| TCTE1 | 0.0489872 | -1.64832 | APCDD1L | 0.0160971 | -2.41235 |
| UCN | 0.0037761 | -1.64946 | DCHS1 | 0.00752015 | -2.43273 |
| TMEM86A | 0.00964896 | -1.64994 | KRT3 | 0.041891 | -2.44482 |
| SART3 | 0.0154546 | -1.65959 | ADH1C | 0.0260881 | -2.45803 |
| GOLGA2P2 | 0.0142093 | -1.67088 | DLK2 | 0.040191 | -2.47085 |
| TRNP1 | 0.0439195 | -1.67769 | GSN | 0.0477098 | -2.47101 |
| HEPH | 0.0381521 | -1.68225 | FLJ46875 | 0.0201755 | -2.47952 |
| MARVELD2 | 0.0381364 | -1.68234 | HMGCS1 | 0.0499847 | -2.575 |
| PLAC9 | 0.0377636 | -1.68649 | TRIB2 | 0.0380756 | -2.64709 |
| SH3KBP1 | 0.0451682 | -1.69882 | LAG3 | 0.0398748 | -2.68553 |
| HPCAL1 | 0.0189406 | -1.69957 | THBS4 | 0.0256252 | -2.7205 |
| SCT | 0.0418042 | -1.71813 | HSD17B2 | 0.0330886 | -2.75754 |
| PRKG1 | 0.0184531 | -1.72592 | ABTB1 | 0.0369234 | -2.85716 |
| RADIL | 0.00662583 | -1.75871 | FAM78A | 0.038982 | -3.11112 |
| C20orf135 | 0.0309732 | -1.76323 | FLJ30698 | 0.0426586 | -3.13743 |
| TMEM37 | 0.0334918 | -1.79774 | RABL2B | 0.00800631 | -3.16736 |
| AGER | 0.0496659 | -1.8104 | ETV4 | 0.0365959 | -3.26767 |
| SHROOM3 | 0.0489757 | -1.82287 | LOC339192 | 0.0342404 | -3.38369 |
| SMO | 0.0380795 | -1.823 | C13orf16 | 0.0248009 | -3.45668 |
| PLCL2 | 0.0359885 | -1.826 | LOC642335 | 0.0265109 | -3.65619 |
| LRRC16B | 0.0159671 | -1.84618 | STMN3 | 0.0318109 | -3.69304 |
| COL14A1 | 0.0286924 | -1.84778 | TMEM176B | 0.0221529 | -3.79105 |
| OGN | 0.0368907 | -1.85259 | C13orf33 | 0.037319 | -4.30016 |
| BFSP1 | 0.0214947 | -1.85693 | TRPA1 | 0.0494467 | -4.73558 |
| METTL12 | 0.003944 | -1.86401 | HBA2 | 0.00661282 | -5.8041 |
| SMAD1 | 0.0403424 | -1.89878 | SIPA1L2 | 0.0337634 | -6.76115 |
| LPIN3 | 0.0474008 | -1.91035 | ICOSLG | 0.0154353 | -6.86364 |
| ARRB1 | 0.014972 | -1.91722 | MMP10 | 0.0472607 | -9.51461 |
| TLE2 | 0.0321331 | -9.55024 | CYP7B1 | 0.0270015 | -10.1216 |

**Supplemental Table 5: mRNAs in severe ASM changed in expression following stimulation with FCS (2.5 %)**

| **Gene** | **P value** | **FC** | **Gene** | **P value** | **FC** |
| --- | --- | --- | --- | --- | --- |
| SAE1 | 0.0315319 | 28.776 | DDN | 0.0128671 | 2.29176 |
| NUDT22 | 0.0383927 | 23.1666 | EDN1 | 0.0104384 | 2.2315 |
| TRIP12 | 0.0374441 | 20.7629 | TAGLN | 0.0487656 | 2.22898 |
| C6orf108 | 0.0247918 | 19.7578 | TMEM88B | 0.0323484 | 2.22675 |
| ACBD6 | 0.0429061 | 18.8501 | LOC389842 | 0.0454044 | 2.21946 |
| RNF216L | 0.0361313 | 17.2554 | E2F2 | 0.0165037 | 2.19929 |
| FAM50A | 0.0396457 | 14.9825 | LSR | 0.0235457 | 2.19467 |
| PSKH1 | 0.0486055 | 14.7079 | ZIC4 | 0.0429361 | 2.18695 |
| SDSL | 0.0225439 | 14.5717 | DA567289 | 0.00549359 | 2.15372 |
| ATAD3A | 0.030444 | 13.8475 | NDUFS8 | 0.00381808 | 2.14945 |
| SNRNP27 | 0.0327279 | 11.8091 | ID4 | 0.0137383 | 2.1279 |
| ASNSD1 | 0.0354113 | 11.2429 | PLCB2 | 0.0184724 | 2.12514 |
| TULP3 | 0.0370215 | 9.8634 | FAM54A | 0.00265674 | 2.11959 |
| OXSM | 0.0301995 | 9.78723 | AFP | 0.0470928 | 2.11115 |
| RAB21 | 0.0491913 | 9.77369 | HIST1H1B | 0.0116681 | 2.10204 |
| ARHGEF17 | 0.0388136 | 9.28091 | ARHGDIB | 0.0162116 | 2.09234 |
| C7orf50 | 0.0253458 | 9.19125 | SGOL2 | 0.00482879 | 2.0429 |
| RPL10AP6 | 0.0442215 | 6.49763 | MMP3 | 0.00927657 | 2.03684 |
| CAPNS2 | 0.040892 | 6.1604 | ENST00000404580 | 0.0151055 | 2.03313 |
| PIGX | 0.0335979 | 5.642 | ACTBL2 | 0.0165729 | 2.02833 |
| LOC283761 | 0.0362225 | 4.92652 | APOBEC3G | 0.00703699 | 2.01329 |
| RELL2 | 0.0303205 | 4.52845 | LIG3 | 0.00210713 | 2.01078 |
| HIST2H3A | 0.0116836 | 4.43854 | DKK1 | 0.00945856 | 2.00856 |
| NAA50 | 0.0304966 | 4.18773 | C18orf50 | 0.0152191 | 2.00559 |
| ACTG2 | 0.0118337 | 4.01062 | FMO2 | 0.0204379 | 1.98753 |
| COMP | 0.0274281 | 3.84419 | LIPN | 0.00158453 | 1.97156 |
| C7orf69 | 0.000417546 | 3.39355 | WDR85 | 0.0288708 | 1.96528 |
| ID1 | 0.00487957 | 3.37276 | CDCA4 | 0.0140678 | 1.95348 |
| ID3 | 0.000162498 | 3.15246 | BLM | 0.00836898 | 1.95115 |
| SERP2 | 0.0458915 | 2.86087 | C19orf36 | 0.0414973 | 1.93632 |
| ASTN2 | 0.000738895 | 2.83591 | EIF4EBP1 | 0.0105519 | 1.93568 |
| DHRS3 | 0.000668734 | 2.7709 | CNN1 | 0.0193895 | 1.90859 |
| SYT3 | 0.0318851 | 2.71504 | ACTL9 | 0.0408739 | 1.90733 |
| GIYD1 | 0.0363437 | 2.70562 | TMEM110 | 0.00729636 | 1.90372 |
| PHF7 | 0.024339 | 2.67045 | ULBP1 | 0.00337033 | 1.9001 |
| LOC100129324 | 0.00911405 | 2.6471 | ALDH1B1 | 0.00183288 | 1.89709 |
| NPW | 0.00445001 | 2.59169 | AC120194.1 | 0.0421492 | 1.89433 |
| SNORA74B | 0.00355454 | 2.59106 | DONSON | 0.00667162 | 1.89237 |
| CDC45L | 0.0219393 | 2.4589 | ELMO3 | 0.0194432 | 1.88445 |
| FAM83D | 0.0106239 | 2.45033 | PXDN | 0.00678331 | 1.87335 |
| FAM183A | 0.034768 | 2.42204 | DGKG | 0.0111504 | 1.87098 |
| PI16 | 0.0174762 | 2.39663 | CDCA8 | 0.0434952 | 1.87042 |
| MMP24 | 0.00989577 | 2.37446 | KLF7 | 0.00991614 | 1.86591 |
| HJURP | 0.0491266 | 2.35793 | PSG8 | 0.0466474 | 1.85618 |
| SLC7A5 | 0.0181817 | 2.332 | C14orf138 | 0.0225217 | 1.85215 |
| SLC17A9 | 0.0280329 | 2.30034 | SNAR-A3 | 0.0274161 | 1.85096 |
| FAM132A | 0.00176382 | 1.84438 | ALPK2 | 0.021769 | 1.63973 |
| LOC100128429 | 0.0289081 | 1.84399 | WARS | 0.012276 | 1.63779 |
| PDLIM7 | 0.0110294 | 1.84335 | HR | 0.010702 | 1.63613 |
| ACTA2 | 0.00446266 | 1.84298 | AIFM2 | 0.0412398 | 1.63467 |
| NPAS1 | 0.000454168 | 1.82921 | TCIRG1 | 0.0178322 | 1.62906 |
| HMMR | 0.0202382 | 1.82606 | TNC | 0.00521396 | 1.62092 |
| CSRP2 | 0.0249357 | 1.8154 | APOL5 | 0.042588 | 1.61928 |
| OIP5 | 0.0242851 | 1.81163 | PRPS1L1 | 0.0330893 | 1.61876 |
| ENPP4 | 0.000291782 | 1.80634 | HEG1 | 0.0292205 | 1.61529 |
| ALDH1A1 | 0.0155456 | 1.79998 | POTEF | 0.0125938 | 1.6145 |
| NDNL2 | 0.0407615 | 1.79555 | ODC1 | 0.0404126 | 1.61089 |
| ABCC3 | 0.00528059 | 1.79542 | TGFBI | 0.0232463 | 1.60382 |
| TACC3 | 0.0146334 | 1.78537 | LOC90246 | 0.0417054 | 1.60312 |
| CDK12 | 0.0403507 | 1.77716 | CDH6 | 0.0273261 | 1.60102 |
| CXorf36 | 0.0399867 | 1.77386 | DKFZp779M0652 | 0.0369208 | 1.5986 |
| PPAPDC1A | 0.00459146 | 1.77208 | LOC391334 | 0.0187152 | 1.59564 |
| KRTAP19-8 | 0.0111527 | 1.768 | SHCBP1 | 0.0125923 | 1.5899 |
| CCNE2 | 0.040152 | 1.76776 | SRPK3 | 0.0395365 | 1.58695 |
| CDH15 | 0.00514977 | 1.76617 | TUBB2A | 0.036036 | 1.58243 |
| CDRT8 | 0.0476002 | 1.76148 | KRT18 | 0.0289952 | 1.57967 |
| EARS2 | 0.026841 | 1.75945 | IL6 | 0.0286464 | 1.57711 |
| TPM1 | 0.00128356 | 1.75561 | ZWINT | 0.0272534 | 1.57286 |
| NUAK2 | 0.0408128 | 1.75403 | ATF6 | 0.0116662 | 1.56936 |
| IGKV2D-26 | 0.0381266 | 1.73547 | TUBGCP4 | 0.0306173 | 1.56756 |
| EXO1 | 0.00344069 | 1.73461 | CHMP5 | 0.0456459 | 1.56092 |
| SH2D4A | 0.0188817 | 1.73319 | PFN1 | 0.00875389 | 1.55958 |
| GPR173 | 0.0293878 | 1.73188 | ANO7 | 0.0434326 | 1.55938 |
| CRYAB | 0.000394639 | 1.72738 | ARRDC4 | 0.042354 | 1.55865 |
| SQLE | 0.0291135 | 1.72525 | PDSS1 | 0.0327617 | 1.54896 |
| MCM6 | 0.00443025 | 1.72247 | PGAM5 | 0.0237636 | 1.54858 |
| UCHL3 | 0.00591859 | 1.72062 | BE897625 | 0.0450089 | 1.54428 |
| MTP18 | 0.0387551 | 1.71943 | IL17REL | 0.0244906 | 1.54247 |
| PKMYT1 | 0.0442163 | 1.70402 | NOP16 | 0.0355891 | 1.53584 |
| FGF1 | 0.0341872 | 1.70077 | EYA2 | 0.0304183 | 1.53301 |
| SDHB | 0.0175247 | 1.6982 | URB2 | 0.00172548 | 1.53158 |
| MGC31957 | 0.0441615 | 1.6915 | OR8B8 | 0.0210497 | 1.52967 |
| CENPM | 0.0455267 | 1.68005 | LOC644686 | 0.0121129 | 1.52698 |
| GATA6 | 0.000328035 | 1.67938 | IL23A | 0.0306401 | 1.5269 |
| C17orf55 | 0.0317488 | 1.67516 | SNAPC2 | 0.0493027 | 1.52311 |
| SLC7A1 | 0.0277587 | 1.67209 | DLEU1 | 0.0397625 | 1.52274 |
| EPHX4 | 0.0457424 | 1.66998 | CCDC134 | 0.012816 | 1.52022 |
| NP511207 | 0.0470343 | 1.66315 | DHODH | 0.0341323 | 1.5189 |
| BATF3 | 0.0474352 | 1.66035 | KIF23 | 0.0385091 | 1.51864 |
| UXT | 0.0200589 | 1.65571 | TUBA1B | 0.0485987 | 1.51811 |
| LOC100133478 | 0.0162422 | 1.65485 | FABP5 | 0.0111229 | 1.51057 |
| MPI | 0.013293 | 1.65459 | ANO9 | 0.0480375 | 1.50866 |
| PRKCI | 0.0103238 | 1.65242 | TNFRSF12A | 0.0253343 | 1.50694 |
| FBXW8 | 0.00214241 | 1.65007 | ASB2 | 0.00227959 | 1.5052 |
| HHEX | 0.00717586 | 1.64445 | AIMP2 | 0.0183562 | 1.50163 |
| NCL | 0.0212408 | 1.64283 | IGJ | 0.0277505 | 1.50158 |
| ZBTB8OSP1 | 0.00288391 | 1.64143 | CD83 | 0.0282823 | -1.50228 |
| TMEM138 | 0.00161397 | 1.64023 | SPTBN4 | 0.0282382 | -1.50281 |
| KCNH2 | 0.0469862 | -1.50303 | DPT | 0.0164097 | -1.6272 |
| SPRY1 | 0.025703 | -1.50499 | TBC1D22B | 0.0116999 | -1.6273 |
| NFKBIA | 0.0164733 | -1.50535 | OPN1SW | 0.0380872 | -1.63001 |
| HEXIM2 | 0.0265515 | -1.50677 | ARVCF | 0.0097111 | -1.63635 |
| KIAA0495 | 0.0424653 | -1.50793 | RASA4 | 0.0404237 | -1.63808 |
| PABPC3 | 0.00939298 | -1.50982 | ETV1 | 0.0172944 | -1.64143 |
| GFRA1 | 0.0271806 | -1.51225 | LOC100132197 | 0.0017269 | -1.64502 |
| KIAA0895L | 0.0408107 | -1.51644 | PLCL2 | 0.0227026 | -1.64503 |
| METTL7A | 0.0308474 | -1.51819 | ZNF29P | 0.00222593 | -1.64536 |
| BTG1 | 0.0412704 | -1.51955 | ADAM8 | 0.0220746 | -1.64573 |
| TRERF1 | 0.0192006 | -1.52049 | SCN2A | 0.0160161 | -1.64932 |
| ZBTB46 | 0.0406111 | -1.52212 | OXER1 | 0.0155099 | -1.65046 |
| RAP2B | 0.0235145 | -1.53124 | MXRA5 | 0.00549818 | -1.65166 |
| PDZRN3 | 0.00504218 | -1.53388 | ADRBK2 | 0.036771 | -1.65168 |
| EPHB3 | 0.0281024 | -1.53478 | PPL | 0.0354628 | -1.65483 |
| NDRG3 | 0.0411172 | -1.53567 | FAM59A | 0.0131217 | -1.65541 |
| KLRG1 | 0.00484315 | -1.53642 | PTPRU | 0.000647812 | -1.65573 |
| ZMIZ2 | 0.00599439 | -1.5395 | PANX3 | 0.0435711 | -1.65639 |
| TMEM120B | 0.00832699 | -1.54006 | CCL27 | 0.023936 | -1.66092 |
| MYOF | 0.0140487 | -1.54498 | CLN8 | 0.0067489 | -1.66444 |
| ANKRD36 | 0.0342401 | -1.54625 | NNAT | 0.0193647 | -1.67641 |
| GAS1 | 0.0342949 | -1.5472 | AGER | 1.80E-05 | -1.6812 |
| UBTF | 0.0463208 | -1.54893 | ARRB1 | 0.0053879 | -1.6816 |
| SNHG7 | 0.0276343 | -1.55065 | ARHGAP6 | 0.0293974 | -1.68299 |
| BACH2 | 0.0422622 | -1.55106 | PHEX | 0.0352096 | -1.68449 |
| EHD3 | 0.017498 | -1.55421 | IRAK2 | 0.0133486 | -1.68593 |
| MRPS6 | 0.0102987 | -1.55456 | SCXA | 0.0441773 | -1.70553 |
| ZNF596 | 0.0323015 | -1.55726 | SGIP1 | 0.0077103 | -1.70833 |
| SNCAIP | 0.00703485 | -1.55821 | NTN1 | 0.00603033 | -1.71195 |
| GRIN3B | 0.00278114 | -1.56203 | CRMP1 | 0.0402138 | -1.71328 |
| DCHS1 | 0.0493557 | -1.56359 | C19orf67 | 0.0442978 | -1.71828 |
| LOC91450 | 0.0149978 | -1.56424 | SYNE2 | 0.0164992 | -1.73058 |
| C14orf139 | 0.0111174 | -1.56482 | CCL15 | 0.0481097 | -1.73132 |
| MAPK10 | 0.0468834 | -1.56652 | BC030764 | 0.0106245 | -1.73984 |
| NPR1 | 0.00656877 | -1.5676 | USP21 | 0.000202573 | -1.74008 |
| GGTLC1 | 0.022507 | -1.57194 | KBTBD3 | 0.0351395 | -1.75224 |
| C3orf65 | 0.00511018 | -1.57454 | PHLDA1 | 0.0157209 | -1.75268 |
| PLGLB1 | 0.0230776 | -1.57603 | LOC92973 | 0.0467445 | -1.75423 |
| OTUD4 | 0.00801676 | -1.58681 | SEMA3D | 0.0390407 | -1.75514 |
| FAM179A | 0.0284816 | -1.58839 | FRMD4A | 0.024925 | -1.75934 |
| MEG8 | 0.00128523 | -1.59095 | CD19 | 0.0104817 | -1.76635 |
| PIK3CA | 0.00813475 | -1.59644 | SH2B2 | 0.00634026 | -1.76713 |
| RNF122 | 0.0131832 | -1.6004 | MTVR2 | 0.0278556 | -1.76758 |
| IFIT1 | 0.0334282 | -1.60155 | C6orf225 | 0.0160706 | -1.77255 |
| JAK2 | 0.0363478 | -1.60258 | KRTAP10-5 | 0.0170766 | -1.77611 |
| C3orf25 | 0.00786974 | -1.60261 | AGBL2 | 0.0258052 | -1.80999 |
| DLGAP3 | 0.0034738 | -1.60553 | SPTLC3 | 0.00397278 | -1.81215 |
| NKX3-2 | 0.00942769 | -1.60586 | AX747836 | 0.00124121 | -1.8345 |
| ZNF263 | 0.0440153 | -1.60785 | PDE4D | 0.0227853 | -1.8369 |
| HBM | 0.0225791 | -1.6087 | NFIA | 0.0319257 | -1.83882 |
| DYTN | 0.021317 | -1.61031 | PLA2R1 | 0.0385392 | -1.83937 |
| TTLL8 | 0.00711562 | -1.6134 | COL4A6 | 0.0234852 | -1.84027 |
| OR4A15 | 0.0102407 | -1.84033 | C3orf58 | 0.0234418 | -2.15172 |
| C12orf70 | 0.0427254 | -1.84058 | SCN7A | 0.00669843 | -2.2424 |
| PPP1R1B | 0.0456885 | -1.84318 | FLJ41484 | 0.0423813 | -2.24948 |
| OR52K2 | 0.0251642 | -1.86224 | SIPA1L2 | 0.0388468 | -2.25607 |
| LEF1 | 0.0189959 | -1.87785 | SYT7 | 0.00680712 | -2.27284 |
| RELB | 0.00251178 | -1.87954 | ZSWIM1 | 0.0193076 | -2.28022 |
| ABCA1 | 0.0478522 | -1.88101 | ZNF165 | 0.0322561 | -2.29244 |
| PAX8 | 0.00654786 | -1.88673 | CHRDL1 | 0.00714013 | -2.33939 |
| PLA2G4F | 0.00644873 | -1.89378 | OLFML2A | 0.0368902 | -2.36171 |
| C7orf53 | 0.0175538 | -1.8955 | AL832161 | 0.0147706 | -2.3768 |
| CRTC1 | 0.00214418 | -1.89906 | LRRC66 | 0.023585 | -2.3824 |
| PCDHB9 | 0.0122211 | -1.90041 | OR2Y1 | 0.0424392 | -2.38439 |
| KILLIN | 0.0391846 | -1.91338 | AX747124 | 0.0432925 | -2.41574 |
| SLC5A3 | 0.015224 | -1.91469 | COL13A1 | 0.00735126 | -2.4369 |
| LOC646936 | 0.0176281 | -1.91957 | POLR2F | 0.0286454 | -2.45184 |
| SP5 | 0.0380915 | -1.92488 | HOXA10 | 0.0173438 | -2.46894 |
| GPR115 | 0.0381257 | -1.92707 | ZNF606 | 0.0102262 | -2.47866 |
| FLJ38717 | 0.0172665 | -1.92854 | KISS1 | 0.0368085 | -2.4838 |
| ENST00000380859 | 0.0172847 | -1.93667 | FLJ13197 | 0.0284087 | -2.50724 |
| STON1-GTF2A1L | 0.0194851 | -1.94012 | USP9X | 0.0205324 | -2.54888 |
| CNIH3 | 0.00835817 | -1.94043 | PLAT | 0.0247061 | -2.56001 |
| ASPRV1 | 0.00875346 | -1.94749 | CENPM | 0.0453517 | -2.605 |
| NXPH3 | 0.0435522 | -1.949 | SDCBP2 | 0.00585325 | -2.66208 |
| GRAMD1A | 0.00926919 | -1.97029 | KIAA2026 | 0.0432449 | -2.74054 |
| TUB | 0.0369388 | -1.98155 | OR1K1 | 0.00985021 | -2.84986 |
| C12orf53 | 0.0234272 | -1.9904 | CILP | 0.00252968 | -2.94239 |
| LOC100129888 | 0.0435407 | -1.99092 | OR10G9 | 0.0212547 | -3.1438 |
| COL4A5 | 0.0448822 | -1.99106 | KRTAP13-2 | 0.0168551 | -3.30306 |
| HBA2 | 0.0324804 | -2.00028 | CCL11 | 0.00678786 | -4.09672 |
| SCGBL | 0.0444717 | -2.00533 | OR6N1 | 0.0249156 | -5.33171 |
| MATN2 | 0.0215137 | -2.02372 | CHST8 | 0.0475643 | -5.62812 |
| PCDHB5 | 0.0288846 | -2.10017 | CD274 | 0.020515 | -5.80621 |
| PRRT2 | 0.0313314 | -2.11684 | HLA-DRB3 | 0.0452149 | -11.3561 |
| PPARGC1A | 0.0307013 | -2.13856 |  |  |  |

**Supplemental Table 6: Common mRNA expression changes in ASM cells from patients with non-severe or severe asthma after exposure with FCS (2.5 %)**

|  |  | Non-Severe Asthma | | Severe Asthma |
| --- | --- | --- | --- | --- |
| Gene Symbol | **Gene Name** | **Microarray (FC)** | **Microarray (FC)** | |
| AFP | Alpha-fetoprotein | 13.6 (p < 0.01) | 2.2 (p < 0.01) | |
| TACC3 | Transforming, acidic coiled-coil containing protein 3 | 10.3 (p < 0.01) | 1.8 (p < 0.01) | |
| ACTG2 | Actin, gamma 2, smooth muscle, enteric | 7.9 (p < 0.01) | 4 (p < 0.01) | |
| PSG5 | Pregnancy specific beta-1-glycoprotein 5 | 5.0 (p < 0.01) | 2 (p < 0.01) | |
| SLC7A5 | Solute carrier family 7 (amino acid transporter light chain, L system), member 5 | 4.9 (p < 0.01) | 2.3 (p < 0.01) | |
| BLM | Bloom syndrome, RecQ helicase-like | 4.5 (p < 0.01) | 2 (p < 0.01) | |
| COMP | Cartilage oligomeric matrix protein | 4.0 (p < 0.01) | 3.8 (p < 0.01) | |
| E2F2 | E2F transcription factor 2 | 3.8 (p < 0.01) | 2.2 (p < 0.01) | |
| C7orf69 | Chromosome 7 open reading frame 69 | 3.7 (p < 0.01) | 3.4 (p < 0.01) | |
| DONSON | Downstream neighbour of SON | 2.7 (p < 0.01) | 1.9 (p < 0.01) | |
| FAM183A | Family with sequence similarity 183, member A | 2.5 (p < 0.01) | 2.4 (p < 0.01) | |
| SLC7A1 | Solute carrier family 7 (cationic amino acid transporter, y+ system), member 1 | 2.1 (p < 0.01) | 1.7 (p < 0.01) | |
| DHODH | Dihydroorotate dehydrogenase (quinone) | 2.0 (p < 0.01) | 1.5 (p < 0.01) | |
| AIFM2 | Apoptosis-inducing factor, mitochondrion-associated, 2 | 1.8 (p < 0.01) | 1.6 (p < 0.01) | |
| AGER | Advanced glycosylation end product-specific receptor | -1.8 (p < 0.01) | -1.7 (p < 0.01) | |
| ARRB1 | Arrestin, beta 1 | -1.9 (p < 0.01) | -1.7 (p < 0.01) | |
| IGKV2D-26 | Immunoglobulin kappa variable 2D-26 | -2.0 (p < 0.01) | 1.7 (p < 0.01) | |
| C14orf138 | Methyltransferase like 21D | -2.3 (p < 0.01) | 1.9 (p < 0.01) | |
| DCHS1 | Dachsous 1 | -2.4 (p < 0.01) | -1.6 (p < 0.01) | |
| HBA2 | Hemoglobin, alpha 2 | -5.8 (p < 0.01) | -2.0 (p < 0.01) | |

**Supplemental Table 7: mRNAs in non-severe ASM changed in expression following treatment with dexamethasone (10^-7^ M), before stimulation with FCS (2.5 %)**

| **Gene** | **P value** | **FC** | **Gene** | **P value** | **FC** |
| --- | --- | --- | --- | --- | --- |
| EGFL6 | 0.0224845 | 9.85829 | CR591103 | 0.0186415 | 3.12462 |
| SCN2B | 0.0284043 | 9.27174 | BC047615 | 0.00346642 | 3.08526 |
| ICAM2 | 0.0145181 | 8.29318 | CPVL | 0.0368554 | 3.05313 |
| CPXM2 | 0.0152785 | 8.15319 | GLYATL1 | 0.00971849 | 3.04558 |
| C3 | 0.0319192 | 7.59113 | RSPH6A | 0.0132347 | 3.0372 |
| FOS | 0.0326376 | 6.21901 | SLC7A2 | 0.023903 | 2.97487 |
| CYP7B1 | 0.00219021 | 5.83924 | PDGFRL | 0.0306045 | 2.96067 |
| MTTP | 4.54E-05 | 5.79869 | UNQ6228 | 0.0132763 | 2.93464 |
| CYTL1 | 0.00570101 | 5.57193 | PAPLN | 0.00211136 | 2.92105 |
| AQP1 | 0.00918987 | 5.30482 | LOC100128343 | 0.00424291 | 2.8933 |
| SHC2 | 0.0229531 | 5.13233 | LOC145783 | 0.0100316 | 2.88 |
| C21orf90 | 0.0235437 | 4.95966 | BEGAIN | 0.017262 | 2.87315 |
| ITM2A | 0.0174152 | 4.79411 | MFSD4 | 0.00521417 | 2.8624 |
| NEFH | 0.0027194 | 4.44272 | OR8S1 | 0.0127905 | 2.8326 |
| AF072164 | 0.0188097 | 4.34572 | LOC100130218 | 0.0434096 | 2.83212 |
| FOSB | 0.0225566 | 4.26843 | LOC389831 | 0.0332219 | 2.80806 |
| EFHB | 0.0107258 | 4.22377 | PACSIN1 | 0.00266411 | 2.80071 |
| RNF212 | 0.0147874 | 3.82536 | LOC100130276 | 0.0300703 | 2.79567 |
| SLC17A9 | 0.0150972 | 3.80832 | MGC20647 | 0.0488981 | 2.76145 |
| TIAM1 | 0.0238606 | 3.67755 | DPRX | 0.0382461 | 2.75117 |
| TMEM211 | 0.00572655 | 3.66668 | IRAK3 | 0.0407267 | 2.74829 |
| UNQ3028 | 0.0173281 | 3.59575 | GRIA3 | 0.022389 | 2.74313 |
| DA759359 | 0.0115304 | 3.34984 | PCDHGB3 | 0.0286299 | 2.73755 |
| MYO15A | 0.0312493 | 3.34296 | LOC651900 | 0.0238231 | 2.72276 |
| LOC440173 | 3.07E-05 | 3.28153 | E00167 | 0.00848514 | 2.69841 |
| FAM184B | 1.01E-05 | 3.23284 | COL4A4 | 0.049413 | 2.69739 |
| LOC392335 | 0.00466136 | 3.22476 | LOC253039 | 0.0100862 | 2.69359 |
| LOC100293193 | 0.0125121 | 3.17947 | DC378344 | 0.0345073 | 2.68776 |
| C7orf29 | 0.0252444 | 3.17744 | CACNG7 | 0.00858398 | 2.67851 |
| LOC100132368 | 0.00272669 | 3.15585 | MORN5 | 0.0141567 | 2.67189 |
| GLB1L | 0.0341164 | 3.1296 | C9orf96 | 0.00116525 | 2.66608 |
| ATP13A1 | 0.0127753 | 2.6656 | CLDN20 | 0.0246253 | 2.31225 |
| CD511677 | 0.0261348 | 2.65122 | RABGAP1 | 0.0229787 | 2.30133 |
| RTP1 | 0.0420071 | 2.63694 | STK31 | 0.0399854 | 2.29911 |
| ARID3C | 0.041896 | 2.62656 | LOC100130454 | 0.0115812 | 2.29336 |
| C1orf187 | 0.0354729 | 2.61687 | SOX8 | 0.0171318 | 2.27971 |
| LOC100133227 | 0.0143937 | 2.60259 | SLC35D3 | 0.00676386 | 2.27606 |
| FAM110B | 0.0152177 | 2.59557 | BX104605 | 0.0389744 | 2.27346 |
| LOC730020 | 0.0204273 | 2.57775 | TRAV7 | 0.0240724 | 2.26266 |
| C5ORF56 | 0.0370495 | 2.5736 | LOC100131744 | 0.0321835 | 2.22234 |
| LOC728347 | 0.0206348 | 2.56986 | SLCO2A1 | 0.0294004 | 2.21862 |
| LOC100128019 | 0.0100883 | 2.5681 | FAM27E1 | 0.0201259 | 2.21555 |
| FAM177B | 0.00951209 | 2.56634 | PUS7L | 0.0101342 | 2.214 |
| DLX6AS | 0.0412825 | 2.55491 | LOC652554 | 0.0366983 | 2.21147 |
| FLJ39061 | 0.00800903 | 2.54887 | BEST3 | 0.00473454 | 2.20322 |
| FAM43B | 0.0118631 | 2.53446 | RAB17 | 0.00510537 | 2.19712 |
| KIAA0485 | 0.00176249 | 2.53122 | TCN1 | 0.0107946 | 2.18241 |
| LOC100132428 | 0.00528968 | 2.52949 | C4orf47 | 0.0237212 | 2.18075 |
| LOC100129292 | 0.00622093 | 2.52809 | BM930849 | 0.00261303 | 2.17787 |
| GJA8 | 0.0331591 | 2.52799 | ASPHD1 | 0.0165594 | 2.17764 |
| ZSWIM5 | 0.00888449 | 2.52752 | FLJ42220 | 0.0271359 | 2.17659 |
| KLKB1 | 0.0147225 | 2.50704 | LOC100129280 | 0.0191425 | 2.17518 |
| KIF26A | 0.0390661 | 2.50692 | BCORL2 | 0.0385956 | 2.1724 |
| UNQ6494 | 0.0209033 | 2.49631 | PRKCE | 0.00770568 | 2.16966 |
| C6orf15 | 0.0465234 | 2.48525 | LOC100129048 | 0.0468273 | 2.16744 |
| FLJ42351 | 0.0305779 | 2.48101 | DLEC1 | 0.00271548 | 2.16663 |
| NFE2 | 0.0161531 | 2.48053 | LMO3 | 0.000173794 | 2.16137 |
| RFPL1S | 0.0338504 | 2.47875 | DUSP9 | 0.0414663 | 2.16082 |
| C12orf50 | 0.0395112 | 2.47662 | SNAR-E | 0.0250995 | 2.15839 |
| BAIAP2L2 | 0.00879244 | 2.46611 | FOXE3 | 0.0292607 | 2.15296 |
| RGPD1 | 0.018853 | 2.46436 | BLID | 0.0348142 | 2.15038 |
| ZFR2 | 0.0407983 | 2.43805 | CCDC110 | 0.0106732 | 2.14595 |
| ASTN1 | 0.000239932 | 2.43532 | D21S2088E | 0.0204949 | 2.14226 |
| PHF7 | 0.0494961 | 2.42975 | TRIM50 | 0.0066816 | 2.13884 |
| ADRA1D | 0.0061145 | 2.4213 | RPS15AP10 | 0.0120046 | 2.13503 |
| LOC100289418 | 0.0364615 | 2.42035 | GGT8P | 0.0301629 | 2.13128 |
| SMTNL2 | 0.00837463 | 2.41454 | HOPX | 0.0196611 | 2.12091 |
| LOC100129380 | 0.0234232 | 2.40886 | LOC100128562 | 0.0349627 | 2.11421 |
| TSPY16P | 0.0442626 | 2.38998 | X51791 | 0.0440148 | 2.11385 |
| LOC729041 | 0.0452279 | 2.38739 | CPAMD8 | 0.024003 | 2.11303 |
| LOC727844 | 0.00878575 | 2.38436 | RHPN1 | 0.0111075 | 2.10808 |
| STAG3 | 0.0342446 | 2.38192 | SLC17A1 | 0.0223298 | 2.10807 |
| TDRD6 | 0.0285918 | 2.3769 | CU691765 | 0.00847318 | 2.10361 |
| LOC100131608 | 0.00697544 | 2.37241 | ZNF334 | 0.017776 | 2.0923 |
| HEPACAM | 0.0243969 | 2.36755 | KCNA6 | 0.00750647 | 2.08436 |
| PAQR5 | 0.0217854 | 2.35641 | TTTY7 | 0.0363452 | 2.07877 |
| LOC100131657 | 0.0294199 | 2.35553 | CHDH | 0.0363727 | 2.07317 |
| IGHV1-58 | 0.028446 | 2.35106 | PLEC | 0.0471604 | 2.06796 |
| C6orf103 | 0.0106071 | 2.3392 | TCL1B | 0.000474969 | 2.06752 |
| TSSK2 | 0.0305164 | 2.32101 | PCDHB6 | 0.0410447 | 2.06634 |
| LOC401561 | 0.0419644 | 2.05753 | PIP5K1A | 0.00247996 | 1.79475 |
| UTP23 | 0.0470728 | 2.04735 | EFR3B | 0.0449516 | 1.78646 |
| LOC439950 | 0.0105374 | 2.04028 | LOC645146 | 0.0343767 | 1.78442 |
| PAIP2B | 0.00970691 | 2.03869 | RAB6B | 0.00298074 | 1.77689 |
| C2orf83 | 0.0229905 | 2.03791 | LOC283911 | 0.0487751 | 1.77496 |
| QSOX2 | 0.0364102 | 2.03504 | TREM2 | 0.0157232 | 1.77476 |
| MYL7 | 0.0495107 | 2.03266 | PROL1 | 0.0126878 | 1.76938 |
| IL1B | 0.00337457 | 2.01875 | LOC100506302 | 0.0348837 | 1.76898 |
| CEACAM7 | 0.00425023 | 2.01502 | C21orf58 | 0.0105458 | 1.75753 |
| CTSL | 0.0164967 | 2.01376 | LOC100288412 | 0.0218689 | 1.74799 |
| C9orf152 | 0.0159139 | 2.00518 | SERPINB12 | 0.0229203 | 1.74729 |
| GPR123 | 0.0133016 | 1.98501 | KRTAP10-12 | 0.0272806 | 1.74726 |
| FHL1 | 0.0258499 | 1.97289 | KRT17 | 0.0331374 | 1.74684 |
| CACNA1I | 0.0177944 | 1.971 | tAKR | 0.0171625 | 1.73752 |
| NET1 | 0.0022538 | 1.9696 | GBX1 | 0.0416199 | 1.73375 |
| CEACAM18 | 0.0115634 | 1.96952 | KU-MEL-3 | 0.0443896 | 1.72162 |
| LOC644075 | 0.044254 | 1.96929 | NECAB1 | 0.0262466 | 1.72067 |
| OLFML1 | 0.00653956 | 1.96783 | PATE2 | 0.0012714 | 1.71786 |
| VMO1 | 0.0138649 | 1.96773 | LOC100131825 | 0.00739712 | 1.71753 |
| MAGEA13P | 0.0177464 | 1.96604 | LOC283174 | 0.0368537 | 1.71613 |
| C8orf67 | 0.017583 | 1.96038 | SBNO2 | 0.0185852 | 1.71416 |
| INHBC | 0.0293898 | 1.95938 | FBXO47 | 0.0303511 | 1.71365 |
| ARHGAP26 | 0.0199751 | 1.9388 | ARHGAP27 | 0.0453791 | 1.70604 |
| LOC154860 | 0.0168305 | 1.93675 | RICH2 | 0.0455539 | 1.70033 |
| CU691877 | 0.00895458 | 1.93611 | CLEC4G | 0.00484712 | 1.69154 |
| IQCH | 0.0168583 | 1.9353 | SLCO2B1 | 0.0350121 | 1.68995 |
| CD244 | 0.0146865 | 1.92737 | LOC100130354 | 0.0499476 | 1.68954 |
| OLFML2B | 0.0121324 | 1.92565 | YIPF3 | 0.0303159 | 1.68865 |
| LOC731312 | 0.0306703 | 1.92023 | TSPAN8 | 0.0252275 | 1.68416 |
| TMEM174 | 0.00551208 | 1.91576 | FAM9A | 0.0325952 | 1.67987 |
| ADAM11 | 0.0208085 | 1.91515 | MAML1 | 0.0347817 | 1.67804 |
| KRTAP23-1 | 0.031784 | 1.9062 | ZNF445 | 0.0271602 | 1.67569 |
| CALML4 | 0.0287723 | 1.90351 | GPR32 | 0.0350271 | 1.67274 |
| SLIT2 | 0.0498067 | 1.89512 | DA666023 | 0.0430406 | 1.67235 |
| LOC339568 | 0.0215577 | 1.89024 | C6orf195 | 0.0242215 | 1.66734 |
| OR6K3 | 0.036102 | 1.88625 | IFNA2 | 0.00802891 | 1.6637 |
| LOC100133089 | 0.0258844 | 1.87293 | LOC100268168 | 0.025986 | 1.65787 |
| PM20D1 | 0.023256 | 1.86964 | NDRG2 | 0.0442569 | 1.65369 |
| ND6 | 0.0371702 | 1.86015 | LOC389033 | 0.0217474 | 1.65276 |
| TPH1 | 0.0307384 | 1.85883 | LOC100130710 | 0.0299083 | 1.65148 |
| RIBC2 | 0.0029066 | 1.85262 | RAB33B | 0.00183748 | 1.6505 |
| LOC651845 | 0.0149557 | 1.85036 | KLC2 | 0.021582 | 1.64417 |
| TBX10 | 0.0337038 | 1.83411 | PRODH | 0.034482 | 1.63989 |
| DKFZP586B0319 | 0.000230914 | 1.82648 | DPYSL5 | 0.00493524 | 1.63763 |
| LOC100128747 | 0.0396306 | 1.82634 | PLD3 | 0.0463304 | 1.6364 |
| KCNH6 | 0.00580284 | 1.81941 | AJAP1 | 0.0302189 | 1.63436 |
| LOC729479 | 7.09E-09 | 1.81097 | FAM81A | 0.0439556 | 1.63142 |
| C10orf67 | 0.0481221 | 1.79864 | POR | 0.0430517 | 1.62831 |
| C1QL4 | 0.0146332 | 1.79761 | DKFZP434I0714 | 5.22E-05 | 1.62454 |
| LOC340017 | 0.000930056 | 1.61948 | UBE4B | 0.000178117 | 1.49723 |
| HEPH | 0.0291872 | 1.61907 | FLJ39051 | 0.0207371 | 1.48876 |
| LOC286058 | 0.0182636 | 1.61866 | ZNF787 | 0.0249678 | 1.47737 |
| CHRNA9 | 0.0052177 | 1.61802 | STK38L | 0.0337557 | 1.47362 |
| LOC729494 | 0.0430389 | 1.61712 | ELOVL7 | 0.0355321 | 1.47254 |
| C22orf32 | 0.000799759 | 1.6171 | C20orf70 | 0.0380086 | 1.47186 |
| KIR3DL2 | 0.0476025 | 1.61614 | BCL9L | 0.0437571 | 1.46794 |
| FAM159A | 0.0271374 | 1.61373 | ANKRD34B | 0.0392712 | 1.46612 |
| SKCG-1 | 0.0470318 | 1.61284 | IL11RA | 0.0240191 | 1.46109 |
| ZNF423 | 0.0359766 | 1.60837 | TRIM29 | 0.0422691 | 1.4454 |
| MAF | 0.0387875 | 1.60742 | TAF10 | 0.00373934 | 1.4429 |
| C9ORF147 | 0.0275508 | 1.60594 | LOC400756 | 0.0254187 | 1.43656 |
| KCNV1 | 7.01E-06 | 1.60464 | ZNF804B | 0.0434655 | 1.43543 |
| LOC100130456 | 0.0302841 | 1.60259 | LOC284373 | 0.0469246 | 1.42999 |
| FREM2 | 0.0030197 | 1.59876 | AB305825 | 0.0266727 | 1.42943 |
| CRISPLD1 | 0.011944 | 1.5938 | SLC4A11 | 0.042295 | 1.42717 |
| KHK | 0.0121562 | 1.58493 | GABRA1 | 0.0420077 | 1.42689 |
| NYNRIN | 0.0421409 | 1.58189 | COL23A1 | 0.0474148 | 1.40249 |
| GOLGA6L2 | 0.0354207 | 1.5813 | COL11A2 | 0.0323107 | 1.39073 |
| PNMA5 | 0.0164874 | 1.57732 | INSL5 | 0.00294359 | 1.38953 |
| C21orf94 | 0.0351323 | 1.57598 | GALR3 | 0.0198297 | 1.37979 |
| SYNGR4 | 0.030568 | 1.56816 | ETFDH | 0.0221111 | 1.37676 |
| DB335107 | 0.0215759 | 1.56787 | LOC728875 | 0.0405595 | 1.37306 |
| FAIM3 | 0.0296525 | 1.56452 | PR47 | 0.0320595 | 1.36965 |
| TPH2 | 0.0209772 | 1.56404 | UBASH3A | 0.0313047 | 1.36855 |
| CNFN | 0.00969497 | 1.56004 | TOR2A | 0.0472085 | 1.36779 |
| RUSC2 | 0.0337211 | 1.55193 | ATP8B3 | 0.00121018 | 1.36592 |
| SHPK | 0.0323088 | 1.55079 | BPIL3 | 0.000446043 | 1.36341 |
| LOC100130288 | 0.000151666 | 1.54996 | LOC100128893 | 0.0465814 | 1.35989 |
| LOC440419 | 0.0122559 | 1.54553 | LST-3TM12 | 0.0271738 | 1.35959 |
| PAQR6 | 0.0409996 | 1.54483 | IL1R2 | 0.0493842 | 1.35455 |
| UNQ3104 | 0.0308211 | 1.54306 | BC038732 | 0.031831 | 1.3537 |
| KCNG3 | 0.0411575 | 1.54151 | TYROBP | 0.0460439 | 1.35297 |
| STAP1 | 0.0350517 | 1.53383 | ATP1A1 | 0.0346405 | 1.35051 |
| LOC388564 | 0.00360479 | 1.5331 | RAD9B | 0.00672859 | 1.34225 |
| CCNYL3 | 0.000321594 | 1.53128 | ESYT3 | 0.0193972 | 1.33735 |
| LOC283516 | 0.0138627 | 1.53019 | TLE1 | 0.0289195 | 1.33728 |
| HSD17B13 | 0.0106886 | 1.53015 | MYH16 | 0.00728031 | 1.33569 |
| SULT4A1 | 0.0308749 | 1.52772 | MFSD2A | 0.0369064 | 1.33288 |
| LOC407835 | 0.0293713 | 1.52761 | RTP3 | 0.0224333 | 1.33233 |
| FOXS1 | 0.0486199 | 1.52565 | LOC100133959 | 0.00243993 | 1.32764 |
| RFX2 | 0.0349621 | 1.52095 | C8orf23 | 0.000126475 | 1.3212 |
| LRCH4 | 0.0418985 | 1.52027 | SARS2 | 0.0177337 | 1.32111 |
| ACTR3C | 0.0155141 | 1.51954 | LOC100129036 | 0.0258009 | 1.31652 |
| ZNF768 | 0.0263864 | 1.51948 | GPR64 | 0.0227623 | 1.30967 |
| HLA-DRB1 | 0.0496063 | 1.51891 | AP1S2 | 0.0304579 | 1.30014 |
| ASB5 | 0.0321277 | 1.51685 | LOC100129449 | 0.00127755 | 1.29849 |
| MAST3 | 0.0400648 | 1.51681 | CV572371 | 0.0161569 | 1.29073 |
| KRT81 | 0.0261695 | 1.51343 | CRISP3 | 0.0271476 | 1.27679 |
| RPE65 | 0.025819 | 1.27407 | TMPRSS11F | 0.0456055 | -1.09864 |
| PGLYRP4 | 0.036499 | 1.26499 | LOC100128889 | 0.0461903 | -1.10646 |
| TMEM44 | 0.0371009 | 1.26306 | ADH6 | 0.0281598 | -1.12724 |
| MUC21 | 0.0109483 | 1.26161 | ZSCAN23 | 0.0368113 | -1.12983 |
| TAOK2 | 0.0325985 | 1.26161 | TIFA | 0.033797 | -1.13958 |
| MED12L | 0.00227837 | 1.25117 | RPP21 | 0.0496079 | -1.18122 |
| POLD4 | 0.045117 | 1.25011 | TTTY12 | 0.0350541 | -1.19127 |
| MRPL55 | 0.0181834 | 1.24403 | PLA2G4D | 0.0346843 | -1.20836 |
| HRNR | 0.0397009 | 1.24037 | LOC339751 | 0.0406197 | -1.21402 |
| LOC100132972 | 0.00405942 | 1.23661 | LOC100128477 | 0.0146921 | -1.22535 |
| UNC5CL | 0.02062 | 1.23546 | IGLV1-44 | 0.0283777 | -1.22657 |
| PARD6B | 0.004883 | 1.23262 | ARFGEF1 | 0.0349307 | -1.23045 |
| LOC339260 | 0.0326179 | 1.22263 | ILF2 | 0.048405 | -1.23527 |
| LRRC70 | 0.00738901 | 1.19213 | ZC3HC1 | 0.0166388 | -1.23692 |
| WDR55 | 0.0472904 | 1.18886 | FLJ45721 | 0.0153009 | -1.25978 |
| OR5M1 | 0.00471608 | 1.18431 | TEX264 | 0.0234891 | -1.27281 |
| FABP9 | 0.0169342 | 1.18204 | RANGRF | 0.0377242 | -1.27903 |
| PLD5 | 0.0443132 | 1.1777 | DTX1 | 0.0257816 | -1.28439 |
| LOC728147 | 0.0428683 | 1.17184 | ARAP1 | 0.0164053 | -1.29282 |
| MYH4 | 0.0093955 | 1.16987 | PCDH8 | 0.0449058 | -1.29606 |
| MAPK4 | 0.000413154 | 1.16784 | RPL14 | 0.0416995 | -1.29855 |
| HCG27 | 0.0431368 | 1.15153 | EIF3K | 0.0327072 | -1.30717 |
| ANKRD19 | 0.0121885 | 1.14466 | COPS6 | 0.0321624 | -1.31843 |
| EXOC6B | 0.0481348 | 1.14053 | BF733045 | 0.00167963 | -1.32042 |
| CTCFL | 0.0174172 | 1.12855 | NDUFA9 | 0.0469431 | -1.32602 |
| FIGLA | 0.0305764 | 1.12619 | PKHD1L1 | 0.0494912 | -1.33834 |
| LOC729409 | 0.0274765 | 1.10431 | MOBP | 0.0170939 | -1.34063 |
| CYP27C1 | 0.0249948 | 1.09287 | ANKRD5 | 0.0147312 | -1.36002 |
| RP11-218C14.6 | 0.0135067 | 1.07401 | CT45A5 | 0.0367247 | -1.36032 |
| ECHDC1 | 0.0154592 | -1.04898 | NEUROD6 | 0.0261876 | -1.36043 |
| LOC401445 | 0.0146153 | -1.05782 | C6orf70 | 0.038348 | -1.36457 |
| ELF5 | 0.00800403 | -1.06654 | TST | 0.0247942 | -1.365 |
| TAAR3 | 0.0233372 | -1.06767 | BIRC8 | 0.0113783 | -1.3714 |
| SPDYE1 | 0.0464789 | -1.06918 | CORO1B | 0.0295777 | -1.37383 |
| LOC100129717 | 0.00881801 | -1.07103 | BAI3 | 0.0260008 | -1.37729 |
| PTCHD1 | 0.0340978 | -1.07122 | NSFL1C | 0.0241619 | -1.37979 |
| IRX1 | 0.00867645 | -1.07144 | C11orf48 | 0.0327563 | -1.38163 |
| WDR72 | 0.0261069 | -1.07253 | PLG | 0.0325968 | -1.38824 |
| TMC5 | 0.011084 | -1.07276 | MRPL16 | 0.00522512 | -1.39013 |
| TTR | 0.0334625 | -1.08564 | CLNS1A | 0.0215771 | -1.39112 |
| LOC285965 | 0.0447548 | -1.08603 | FLJ45872 | 0.0380872 | -1.39117 |
| PRR23A | 0.0488675 | -1.08614 | LOC100134139 | 0.0292488 | -1.39632 |
| C1orf105 | 0.0067356 | -1.09049 | POU3F2 | 0.0238103 | -1.40624 |
| CLEC4GP1 | 0.0400279 | -1.09058 | OBFC1 | 0.0350366 | -1.4104 |
| SMEK3P | 0.0365378 | -1.09113 | SLC15A1 | 0.0245224 | -1.43041 |
| OR51B4 | 0.0416735 | -1.09296 | C6orf164 | 0.0463458 | -1.43086 |
| MS4A8B | 0.0287862 | -1.09366 | MRPL3 | 0.028822 | -1.43289 |
| PDILT | 0.0352373 | -1.09781 | MRPL12 | 0.0189764 | -1.43367 |
| TNFAIP8L2 | 0.0415126 | -1.09822 | CTAGE4 | 0.0318006 | -1.43402 |
| C2orf27B | 0.0127679 | -1.45544 | GPX6 | 0.0300355 | -1.6865 |
| COX5A | 0.0203388 | -1.45682 | SDC2 | 0.0167533 | -1.68738 |
| FUBP1 | 0.0431114 | -1.4631 | PCIF1 | 0.0215369 | -1.6876 |
| MYRIP | 0.0112902 | -1.47356 | UBA6 | 0.0366945 | -1.69066 |
| ESD | 0.0275872 | -1.47665 | CDA | 0.0424501 | -1.69099 |
| TP53TG5 | 0.0219031 | -1.47881 | SFTPA1 | 0.0258629 | -1.69425 |
| RSBN1L | 0.0265224 | -1.48784 | ZBTB16 | 0.0187443 | -1.69934 |
| NAA38 | 0.0380492 | -1.48932 | FASTKD2 | 0.0284946 | -1.69956 |
| TFAM | 0.0067683 | -1.49219 | BEND6 | 0.0327597 | -1.70459 |
| DUOXA2 | 0.0347074 | -1.49359 | IGKV1D-16 | 0.00625904 | -1.71148 |
| MGC15705 | 0.0364406 | -1.49375 | PPHLN1 | 0.0244872 | -1.71436 |
| TCFL5 | 0.0375455 | -1.51012 | ANKRD34A | 0.039789 | -1.71619 |
| OTOS | 0.016899 | -1.52633 | MRPL48 | 0.0392436 | -1.72211 |
| DCHS2 | 0.0253431 | -1.53272 | SFTA2 | 0.00982478 | -1.7225 |
| SDSL | 0.0347529 | -1.54678 | ABCB7 | 0.0249674 | -1.72458 |
| FAM171A2 | 0.01809 | -1.54799 | FLJ37798 | 0.0473696 | -1.72555 |
| USP4 | 0.00211787 | -1.54909 | AP1M2 | 0.0366944 | -1.7276 |
| SRRD | 0.0168979 | -1.55799 | TYW3 | 0.0408083 | -1.74336 |
| LOC221710 | 0.000955977 | -1.56203 | CAPNS2 | 0.030342 | -1.74481 |
| ABCC6P1 | 0.0251204 | -1.56227 | UTP6 | 0.0309216 | -1.75103 |
| SLC12A5 | 0.0273618 | -1.56248 | LPO | 0.0241068 | -1.75458 |
| APOM | 0.000720936 | -1.56283 | STIM2 | 0.0251011 | -1.75954 |
| ARMCX6 | 0.0399629 | -1.56553 | ZNF420 | 0.00769476 | -1.76578 |
| LOC100132874 | 0.0348226 | -1.57066 | KIF18A | 0.0449543 | -1.77577 |
| TMEM223 | 0.0349219 | -1.57095 | AFMID | 0.0381957 | -1.77832 |
| NMNAT1 | 0.01655 | -1.57297 | GLS2 | 0.0147042 | -1.78134 |
| RAB4B | 0.00832978 | -1.57885 | LOC286299 | 0.0295837 | -1.78713 |
| STYXL1 | 0.0157409 | -1.58396 | CYP46A1 | 0.00876744 | -1.79645 |
| RP11-403I13.9 | 0.0445731 | -1.58819 | C9orf140 | 0.008924 | -1.79783 |
| CCNH | 0.0419571 | -1.59265 | STAT4 | 0.0388631 | -1.79812 |
| ZNF187 | 0.0347383 | -1.5933 | NDUFS3 | 0.0193552 | -1.80603 |
| SIX3 | 0.0414476 | -1.60428 | PFKFB1 | 0.0298504 | -1.81156 |
| EAPP | 0.0376325 | -1.60634 | SDHB | 0.0100156 | -1.81744 |
| TPRKB | 0.0366331 | -1.6117 | SFRS3 | 0.0402468 | -1.81989 |
| FLJ34208 | 0.0476374 | -1.62624 | C6orf108 | 0.0133844 | -1.83204 |
| NCRNA00160 | 0.0401118 | -1.63183 | C16orf74 | 0.00862393 | -1.83255 |
| B3GALT1 | 0.0303402 | -1.63266 | NKD2 | 0.0348643 | -1.83698 |
| C1QTNF3 | 0.0230505 | -1.63381 | GUCY1A3 | 0.0460035 | -1.847 |
| PCDH12 | 0.0248739 | -1.63423 | LECT2 | 0.0185627 | -1.852 |
| ZNF778 | 0.0497829 | -1.63921 | MALT1 | 0.0234089 | -1.854 |
| OSBP2 | 0.0242293 | -1.64528 | NLRP10 | 0.0270145 | -1.86595 |
| TRIP12 | 0.0240859 | -1.66324 | VAV1 | 0.0308983 | -1.87005 |
| LOC391767 | 0.04405 | -1.66609 | VLDLR | 0.0487244 | -1.87175 |
| FLJ16171 | 0.0478968 | -1.66854 | GPR20 | 0.0442183 | -1.87301 |
| ZNF607 | 0.032604 | -1.672 | HEBP2 | 0.0494134 | -1.87345 |
| DNAH2 | 0.044721 | -1.67467 | LOC100131542 | 0.00693669 | -1.87349 |
| RNASEH2B | 0.0370652 | -1.67546 | LOC150568 | 0.0468719 | -1.87393 |
| TIMM44 | 0.00107801 | -1.67683 | TMEM60 | 0.044222 | -1.87516 |
| LOC100133224 | 0.0132813 | -1.6781 | NOS1AP | 0.0143965 | -1.87874 |
| PCDHAC1 | 0.0263291 | -1.89643 | RASAL2 | 0.00892127 | -2.18315 |
| CDCA3 | 0.048928 | -1.89865 | VN1R1 | 0.0115255 | -2.2053 |
| CR607463 | 0.0242893 | -1.90138 | FLJ45950 | 0.011803 | -2.20917 |
| CAB39L | 0.0375589 | -1.90893 | ZNF77 | 0.0490546 | -2.21015 |
| PPM1L | 0.0465441 | -1.90908 | DONSON | 0.0208259 | -2.22181 |
| SAE1 | 0.00682671 | -1.91643 | TADA1 | 0.0134694 | -2.22358 |
| RTF1 | 0.0359386 | -1.91712 | LOC100335030 | 0.0401339 | -2.23138 |
| EXOC5 | 0.0436936 | -1.91726 | PRINS | 0.0294081 | -2.25029 |
| OR6A2 | 0.0373859 | -1.92839 | DCAF15 | 0.0335145 | -2.25425 |
| C6orf150 | 0.0255693 | -1.93105 | DEFB128 | 0.0332666 | -2.28786 |
| STOX1 | 0.04999 | -1.93657 | CD36 | 0.0499506 | -2.29363 |
| FCRLB | 0.0442974 | -1.95086 | CHN2 | 0.0129252 | -2.29944 |
| MAP6D1 | 0.0193414 | -1.96376 | ECEL1 | 0.00335713 | -2.3002 |
| INPP5B | 0.00978147 | -1.96507 | KCNRG | 0.00372492 | -2.31241 |
| LOC201477 | 0.0199036 | -1.96661 | VILL | 0.00186831 | -2.3235 |
| KCNU1 | 0.0186844 | -1.97358 | LPCAT2 | 0.00257542 | -2.32538 |
| ZNF583 | 0.0306699 | -1.97481 | INTS8 | 0.0396496 | -2.33448 |
| DCTN3 | 0.0218782 | -1.97551 | LOH12CR1 | 0.0101759 | -2.34799 |
| SUV420H1 | 0.00836247 | -1.98208 | AVL9 | 0.00787749 | -2.35523 |
| SCAPER | 0.0124862 | -1.99993 | ILKAP | 0.0490355 | -2.35916 |
| FASTKD3 | 0.0240289 | -2.00515 | ACPP | 0.0487522 | -2.37048 |
| CENPT | 0.0388359 | -2.00572 | NKX6-1 | 0.0241127 | -2.37093 |
| LCE3B | 0.0310833 | -2.00984 | SHC4 | 0.035591 | -2.39069 |
| ZNF266 | 0.0156899 | -2.01565 | CR612090 | 0.0170573 | -2.39411 |
| ANGPT4 | 0.0425998 | -2.02442 | KCTD14 | 0.0232979 | -2.40181 |
| SLC27A4 | 0.0225529 | -2.02761 | LOC374443 | 0.0418283 | -2.41002 |
| STAMBPL1 | 0.0425329 | -2.02813 | DSCC1 | 0.0445259 | -2.41409 |
| OPTC | 0.031607 | -2.03657 | DHDDS | 0.00260125 | -2.44501 |
| LOC144742 | 0.0334827 | -2.0412 | OR4K14 | 0.0404942 | -2.49172 |
| LOC440518 | 0.0242885 | -2.04559 | FMN2 | 0.0380029 | -2.52012 |
| TACR3 | 0.00414029 | -2.04981 | SHMT1 | 0.0250764 | -2.53136 |
| LOC285740 | 0.0281476 | -2.06003 | FLJ33360 | 0.0307568 | -2.55112 |
| LOC727900 | 0.005461 | -2.07472 | FAM26E | 0.0158991 | -2.56312 |
| TDO2 | 0.0217135 | -2.07555 | INPP4A | 0.0300925 | -2.57306 |
| ADCY2 | 0.014242 | -2.0777 | GAB1 | 0.0326721 | -2.60154 |
| LOC402377 | 0.027925 | -2.08423 | ZNF142 | 0.0167231 | -2.61827 |
| ASNSD1 | 0.0363381 | -2.09126 | BTBD19 | 0.0167944 | -2.63699 |
| SNORA59B | 0.0178836 | -2.1052 | CEP97 | 0.00114581 | -2.64601 |
| ASCL1 | 0.0481069 | -2.10654 | DBF4 | 0.023114 | -2.67845 |
| C7orf65 | 0.0471925 | -2.11276 | CCDC89 | 0.0461908 | -2.69102 |
| GPR183 | 0.0426155 | -2.12437 | ZNF208 | 0.0176612 | -2.69905 |
| SLC17A3 | 0.0139654 | -2.12506 | FAM83D | 0.0444039 | -2.74292 |
| ZNF430 | 0.0478224 | -2.12801 | POLE2 | 0.0477631 | -2.85062 |
| C9orf125 | 0.012377 | -2.13302 | PITPNM2 | 0.00883297 | -2.85263 |
| GYG2 | 0.040705 | -2.13839 | LOC100290819 | 0.0115081 | -2.857 |
| PRX | 0.0117804 | -2.13843 | TRMT13 | 0.0397206 | -2.86425 |
| ZNF823 | 0.0235157 | -2.14283 | LOC552889 | 0.041421 | -2.88387 |
| CASD1 | 0.0458806 | -2.15138 | LOC100132658 | 0.0040329 | -2.91454 |
| PECAM1 | 0.0389329 | -2.18241 | COPG2 | 0.0175791 | -2.93662 |
| SLC32A1 | 0.0118395 | -2.93921 | ZNF700 | 0.0127506 | -3.53078 |
| CB962925 | 0.0406413 | -2.94258 | ZNF660 | 0.0185591 | -3.55258 |
| MIR17HG | 0.00503779 | -2.96279 | KCTD16 | 0.026767 | -3.62981 |
| FGD4 | 0.00460589 | -2.97469 | KCNK18 | 0.0288435 | -3.67756 |
| KIAA1383 | 0.0349422 | -2.99342 | PLEK2 | 0.031753 | -3.67869 |
| SLA | 0.0124339 | -2.99922 | DEPDC1 | 0.040848 | -3.71229 |
| ACSL6 | 0.00167299 | -3.03124 | RGAG1 | 0.00415394 | -3.84478 |
| LOC100130176 | 0.0285593 | -3.0509 | CXORF67 | 0.0146076 | -3.87188 |
| CXORF28 | 0.0288017 | -3.05342 | C18orf54 | 0.00363234 | -3.87393 |
| SPP1 | 0.0396132 | -3.06286 | AMBP | 0.0107961 | -3.92483 |
| OR11H6 | 0.00187437 | -3.09524 | ZNF536 | 0.0430415 | -3.94608 |
| MMP16 | 0.000340787 | -3.10346 | USF1 | 0.0150606 | -4.00724 |
| ACTR5 | 0.00655942 | -3.10794 | HSF2BP | 0.0443773 | -4.02576 |
| ABHD3 | 0.013552 | -3.11161 | OR10AD1 | 0.0159812 | -4.1007 |
| LOC729983 | 0.0484273 | -3.1273 | IGLV3-22 | 0.00322099 | -4.15799 |
| ZNF280A | 0.00132765 | -3.1666 | SOX6 | 0.0124537 | -4.36315 |
| LOC646993 | 0.0471719 | -3.20639 | SALL1 | 0.0120465 | -5.30655 |
| EPHX4 | 0.0273351 | -3.23078 | SYT1 | 0.00941826 | -5.56205 |
| FLVCR1 | 0.00126143 | -3.33992 | MLC1 | 0.0306403 | -6.24952 |
| NCOA2 | 0.027666 | -3.3441 | LOC100240735 | 0.00225577 | -6.34631 |
| DKK2 | 0.0139107 | -3.43965 | UCP2 | 0.00701355 | -6.60541 |
| GPR37 | 0.011303 | -3.46342 | LOC100144602 | 0.0192818 | -7.04829 |
| C14orf50 | 0.00235508 | -3.47589 | MND1 | 0.0128997 | -7.28275 |
| SNRNP27 | 0.00145885 | -3.50165 |  |  |  |

**Supplemental Table 8: mRNAs in severe ASM changed in expression following treatment with dexamethasone (10^-7^ M), before stimulation with FCS (2.5 %)**

| **Gene** | **P value** | **FC** | **Gene** | **P value** | **FC** |
| --- | --- | --- | --- | --- | --- |
| SAE1 | 0.0293833 | 30.8509 | COL4A4 | 0.00861455 | 5.08209 |
| FKBP5 | 1.75E-06 | 25.891 | GPM6B | 0.0108153 | 4.98446 |
| NUDT22 | 0.0357378 | 24.8109 | TIMP4 | 0.00176917 | 4.81366 |
| TRIP12 | 0.0336078 | 22.9363 | WFDC1 | 0.00892648 | 4.76096 |
| GGT5 | 0.000192039 | 18.7544 | ENST00000379816 | 0.000181883 | 4.72378 |
| RNF216L | 0.0330866 | 18.6092 | SORT1 | 6.23E-05 | 4.67066 |
| DCTN3 | 0.0457529 | 18.0032 | MAP2 | 0.00126428 | 4.65171 |
| C6orf108 | 0.027999 | 17.8552 | KCNK6 | 0.0139623 | 4.64616 |
| FAM50A | 0.0351623 | 16.5548 | FAM83D | 0.00107002 | 4.60643 |
| ZBTB16 | 0.00307208 | 16.2454 | RNF144B | 0.0151476 | 4.57625 |
| IGFBP2 | 0.00274071 | 15.7287 | CDIPT | 0.0426481 | 4.56102 |
| PSKH1 | 0.0467332 | 15.2121 | PTX3 | 0.000904222 | 4.49363 |
| FAM107A | 0.00653331 | 14.7382 | INHBB | 0.0266491 | 4.43562 |
| ATAD3A | 0.0297641 | 14.0888 | SAMHD1 | 0.000396778 | 4.24656 |
| ARHGEF17 | 0.0272866 | 11.8389 | COL8A1 | 0.000775198 | 4.22817 |
| RASL11B | 0.00210506 | 11.7764 | ERRFI1 | 8.50E-06 | 4.21409 |
| ITGA10 | 0.0019372 | 11.7542 | MT1X | 0.000333054 | 4.20059 |
| CORIN | 0.00111639 | 11.3277 | COMP | 0.0219541 | 4.19289 |
| SNRNP27 | 0.0348066 | 11.294 | MOBKL2B | 0.0457821 | 4.15896 |
| ASNSD1 | 0.0364774 | 11.0048 | MT1M | 0.000145813 | 4.13462 |
| TULP3 | 0.036178 | 10.0216 | RELL2 | 0.0377615 | 4.11776 |
| OXSM | 0.0312126 | 9.57611 | FOXO1 | 0.00100229 | 4.10549 |
| SPARCL1 | 0.000351684 | 8.71945 | IFI44L | 0.00160796 | 4.10517 |
| SDSL | 0.0469366 | 8.65442 | FBN2 | 0.00045236 | 4.09649 |
| MAOA | 4.71E-05 | 8.65072 | ID3 | 6.30E-05 | 4.04241 |
| ID1 | 0.000380069 | 8.50726 | GABBR2 | 0.00244403 | 4.04052 |
| MESDC1 | 0.0343102 | 8.06953 | MT1H | 0.000234725 | 4.02909 |
| LOC100128054 | 0.000177149 | 8.04811 | GALNTL2 | 0.00651786 | 4.02419 |
| CACNB2 | 5.95E-05 | 7.71055 | CTGF | 0.00172937 | 4.01877 |
| C7orf50 | 0.0370643 | 7.28522 | LEMD2 | 0.0426577 | 3.97875 |
| C13orf15 | 0.00154918 | 6.93071 | TSC22D3 | 1.14E-05 | 3.93961 |
| ADRA1B | 0.00508932 | 6.89065 | DUSP1 | 0.000455907 | 3.93387 |
| ACTG2 | 0.00301672 | 6.88999 | NP274062 | 0.00209432 | 3.92619 |
| PIGX | 0.0233662 | 6.82221 | FAM196A | 0.0393619 | 3.89455 |
| CAPNS2 | 0.0353172 | 6.69009 | C10orf10 | 0.0167328 | 3.89057 |
| ENST00000399893 | 0.0441167 | 6.50664 | GADD45B | 0.000273981 | 3.84901 |
| RP3-402G11.5 | 0.0460247 | 6.50258 | SCRG1 | 0.0429723 | 3.83316 |
| GPX3 | 0.000199301 | 6.36918 | NAA50 | 0.0379298 | 3.82847 |
| B3GALT2 | 0.0181628 | 6.27837 | RASAL2 | 0.0290488 | 3.81753 |
| SPINT2 | 0.0154338 | 6.07396 | PER1 | 0.000613973 | 3.75164 |
| GLUL | 8.88E-06 | 6.04048 | SUSD2 | 0.000676035 | 3.70154 |
| OMD | 0.00035564 | 5.82124 | MT1A | 0.000541503 | 3.66803 |
| FLJ37798 | 0.0408714 | 5.56809 | MT1L | 0.000574419 | 3.62364 |
| RASL11A | 0.0003236 | 5.34133 | HSPA2 | 0.0140091 | 3.58406 |
| FMO2 | 0.000465789 | 5.27339 | MT1B | 0.000402572 | 3.55976 |
| HSD11B1 | 0.000577978 | 5.14342 | TAGLN | 0.00998786 | 3.48216 |
| CCK | 0.0357132 | 3.47118 | SNORA74B | 0.00309697 | 2.67189 |
| NEXN | 0.000673327 | 3.45126 | MYADM | 0.00209306 | 2.64763 |
| MT1E | 0.0001665 | 3.44808 | LOC100129104 | 0.0379053 | 2.64592 |
| CDH4 | 0.0278858 | 3.44373 | YIF1B | 0.00912271 | 2.64548 |
| ADAMTS1 | 0.00316131 | 3.38848 | STMN2 | 0.0429079 | 2.63713 |
| USP53 | 0.00533808 | 3.37808 | GCNT1 | 0.000525352 | 2.6321 |
| ALCAM | 0.000415557 | 3.37174 | LOC643650 | 0.0230739 | 2.61701 |
| TRNP1 | 0.000101656 | 3.36238 | CTSC | 0.0125391 | 2.59394 |
| C2orf81 | 0.0471609 | 3.36115 | SRGN | 0.0113194 | 2.5707 |
| ARHGDIB | 0.00210903 | 3.34564 | CRISPLD2 | 0.0144604 | 2.56427 |
| FMO3 | 0.0200423 | 3.33518 | OR1N1 | 0.0213216 | 2.55823 |
| PRUNE2 | 0.00292861 | 3.33047 | C13orf1 | 0.0216735 | 2.54744 |
| IMPA2 | 0.00106513 | 3.32463 | CYR61 | 0.0149191 | 2.52572 |
| NEDD9 | 0.00515643 | 3.31 | EDN1 | 0.00588036 | 2.5197 |
| FIBIN | 0.000939865 | 3.28638 | TMPPE | 0.0427563 | 2.51672 |
| ASTN2 | 0.000400936 | 3.28043 | RPS6KA2 | 0.00023674 | 2.51198 |
| MMD | 0.00132298 | 3.24091 | LOC100130433 | 0.0277066 | 2.50269 |
| GIYD1 | 0.020399 | 3.23461 | ACTBL2 | 0.00608095 | 2.485 |
| ID4 | 0.00231071 | 3.18559 | ENDOD1 | 0.0212248 | 2.48295 |
| ITIH3 | 0.0022738 | 3.15811 | TCEAL4 | 0.000119079 | 2.47177 |
| ITGA1 | 3.24E-05 | 3.07376 | C5orf62 | 0.00726222 | 2.46289 |
| OXTR | 0.0354296 | 3.0722 | NPW | 0.00563788 | 2.46064 |
| GPC4 | 0.00987549 | 3.05569 | DNAJB4 | 0.000156835 | 2.45446 |
| FBXL16 | 0.0343697 | 3.05342 | CALCOCO2 | 8.49E-05 | 2.43369 |
| DHRS3 | 0.000445054 | 3.04017 | DUSP5 | 0.0116667 | 2.42986 |
| AOX1 | 0.00432776 | 3.03646 | PTPLB | 0.00205711 | 2.42043 |
| FSTL3 | 0.00037622 | 2.93921 | C19orf36 | 0.0149041 | 2.41685 |
| FAM105A | 0.000683306 | 2.93841 | DNAJB13 | 0.0278071 | 2.41509 |
| CRYAB | 1.55E-05 | 2.90751 | CCDC68 | 0.00352976 | 2.39002 |
| ACTN4 | 0.0071471 | 2.89902 | ANPEP | 0.0140188 | 2.37735 |
| ADARB1 | 0.0169215 | 2.89265 | ENST00000402541 | 0.0293413 | 2.37316 |
| PRODH | 0.00432797 | 2.88292 | AB072904 | 0.0134451 | 2.37294 |
| SYNPO2 | 0.00245813 | 2.86211 | C6orf145 | 0.00378103 | 2.36902 |
| DNAJC6 | 0.0174459 | 2.84397 | NNMT | 0.00245006 | 2.36272 |
| ANGPTL1 | 0.000998193 | 2.84395 | ALDH1A1 | 0.00328273 | 2.36271 |
| LOC285300 | 0.00217058 | 2.83903 | LOC100133047 | 0.00604439 | 2.34181 |
| LOC729314 | 0.0169639 | 2.83454 | ABCC3 | 0.00102183 | 2.33761 |
| ACTA2 | 0.000410772 | 2.82078 | KCNS3 | 0.00568082 | 2.332 |
| PLA2G2D | 0.0402534 | 2.81014 | APOD | 0.00605547 | 2.33058 |
| MCOLN2 | 0.0285976 | 2.77763 | COL7A1 | 0.0112029 | 2.32901 |
| AX747335 | 0.0368348 | 2.77485 | CDC45L | 0.0276114 | 2.32437 |
| KLF9 | 2.05E-05 | 2.74138 | ALDH1B1 | 0.000519545 | 2.32301 |
| DDAH1 | 0.0117291 | 2.74024 | MORF4L2 | 0.000383087 | 2.31079 |
| DMD | 0.0234342 | 2.73938 | DKFZp686L14188 | 0.0417111 | 2.30624 |
| THC2512536 | 0.0194684 | 2.73473 | MYL9 | 0.0106104 | 2.29158 |
| F3 | 0.0171058 | 2.72834 | CST5 | 0.015985 | 2.28543 |
| CLIC3 | 8.50E-05 | 2.70758 | TEX2 | 0.00158427 | 2.27641 |
| VGF | 0.00536216 | 2.70377 | SYTL4 | 0.00441957 | 2.27422 |
| CDC42EP3 | 0.0062412 | 2.70142 | ADM | 0.00242228 | 2.27403 |
| TXNRD1 | 0.0387953 | 2.26557 | RABL2B | 0.0304508 | 2.09569 |
| SLC26A6 | 0.0030031 | 2.2592 | ENST00000391684 | 0.0256501 | 2.09526 |
| PDLIM5 | 0.00641439 | 2.2577 | COL4A1 | 0.000461987 | 2.09426 |
| AKAP2 | 0.0179959 | 2.25682 | TMEM204 | 0.0051942 | 2.09185 |
| CPPED1 | 4.89E-05 | 2.2549 | HIGD1A | 0.00200655 | 2.09133 |
| SPON1 | 0.00945096 | 2.24465 | PHF17 | 0.000722498 | 2.08993 |
| ENST00000390431 | 0.0157644 | 2.23779 | NDUFS8 | 0.00451451 | 2.0872 |
| ACTN3 | 0.0342007 | 2.23762 | PDLIM7 | 0.00526287 | 2.08165 |
| TMEM88B | 0.0321441 | 2.23007 | CORO6 | 0.00796894 | 2.07296 |
| MT1G | 0.000196228 | 2.2281 | BCAT2 | 2.85E-05 | 2.0727 |
| EPSTI1 | 0.0136577 | 2.22378 | TMEM47 | 0.00940008 | 2.07108 |
| ATP10A | 0.00434919 | 2.2225 | CITED2 | 0.00632978 | 2.07009 |
| ENST00000432803 | 0.0312689 | 2.22134 | MTP18 | 0.0135319 | 2.06869 |
| GDNF | 0.00269501 | 2.21216 | HPD | 0.00988168 | 2.06862 |
| ELMO3 | 0.0081153 | 2.21138 | ZDHHC23 | 0.00079984 | 2.06565 |
| SERPINE1 | 0.00445858 | 2.20768 | ST8SIA3 | 0.029852 | 2.0624 |
| ARMC8 | 3.76E-05 | 2.20455 | TCEAL1 | 0.000157088 | 2.05244 |
| NUAK2 | 0.0120228 | 2.20189 | ZCCHC6 | 0.00543392 | 2.05179 |
| ITGA5 | 0.00848922 | 2.19775 | CFL1 | 0.00803712 | 2.05126 |
| DIO3OS | 0.042181 | 2.19679 | FGD4 | 0.000368076 | 2.05079 |
| SLC17A9 | 0.034645 | 2.18829 | PFN1 | 0.00109584 | 2.05005 |
| DAAM2 | 0.000143499 | 2.18418 | LDHA | 0.00034808 | 2.04853 |
| MYL2 | 0.0120666 | 2.18221 | PCDH7 | 0.0217007 | 2.04643 |
| METTL7A | 0.00263576 | 2.17311 | TXNRD2 | 0.0432052 | 2.04268 |
| KIAA0408 | 0.0299493 | 2.1718 | TBX10 | 0.00410111 | 2.04007 |
| MYC | 0.00142509 | 2.16842 | KLF7 | 0.00575005 | 2.03958 |
| BATF3 | 0.0105979 | 2.16133 | THBS1 | 0.021792 | 2.03869 |
| TLN1 | 0.00532275 | 2.15891 | BAIAP2L2 | 0.0460722 | 2.03592 |
| ASPN | 0.0186281 | 2.15457 | MCAM | 0.0218424 | 2.03524 |
| THC2499666 | 0.0167206 | 2.14283 | PHC2 | 0.000105738 | 2.03368 |
| PAWR | 0.00517323 | 2.14082 | MGP | 0.00528811 | 2.02627 |
| MT2A | 0.0311945 | 2.13848 | PRKAG2 | 0.00351825 | 2.02516 |
| PTPRJ | 0.000263112 | 2.13055 | CD513837 | 0.00266014 | 2.02135 |
| PIK3R1 | 0.00136677 | 2.12926 | THC2631347 | 0.046344 | 2.01857 |
| FAM54A | 0.00260018 | 2.12741 | SQLE | 0.0114418 | 2.01714 |
| LMOD1 | 0.000458295 | 2.12602 | PSME3 | 0.00232806 | 2.017 |
| CELF5 | 0.0318365 | 2.12593 | KLF5 | 0.0425953 | 2.01452 |
| FZD6 | 0.000822425 | 2.12475 | GPD1L | 0.000976915 | 2.01167 |
| C7orf69 | 0.00373963 | 2.12408 | LOC391334 | 0.00369431 | 2.01149 |
| GFPT2 | 4.69E-05 | 2.12213 | ELANE | 0.0498767 | 2.00882 |
| HIF1A | 0.00416025 | 2.11409 | LIPN | 0.00140853 | 2.00722 |
| KIR3DP1 | 0.0497948 | 2.10917 | STK17B | 0.00347825 | 2.00367 |
| LMCD1 | 0.00163141 | 2.10535 | PPME1 | 0.0295785 | 2.00351 |
| MMP24 | 0.0178651 | 2.10029 | ING2 | 0.0094718 | 2.00301 |
| B3GNT5 | 0.00443162 | 2.09994 | LOC646048 | 0.00869057 | 2.00188 |
| LBH | 0.00905975 | 2.09928 | C21orf122 | 0.00156753 | 1.99988 |
| UQCRC1 | 0.000891989 | 2.09869 | ODZ2 | 0.0350808 | 1.99767 |
| C5orf58 | 0.00164073 | 2.09774 | CD302 | 0.00165848 | 1.99728 |
| RXFP3 | 0.0482927 | 2.09724 | HPS5 | 0.00254274 | 1.98919 |
| ZNF828 | 0.00708639 | 1.98828 | TUBB6 | 0.00762767 | 1.88766 |
| LOC100129536 | 0.00144269 | 1.98684 | SYDE1 | 0.0052336 | 1.88589 |
| TMEM64 | 0.00237239 | 1.9828 | C1orf133 | 0.0334995 | 1.88411 |
| C14orf56 | 0.045008 | 1.97702 | NOP16 | 0.00842041 | 1.88205 |
| MMP15 | 0.00054594 | 1.97466 | SEPN1 | 0.0248979 | 1.88112 |
| DKK1 | 0.010454 | 1.97412 | LRRC16A | 0.0249664 | 1.88084 |
| PXDN | 0.00486628 | 1.97317 | LOC100129324 | 0.0443199 | 1.87754 |
| NFXL1 | 0.0153216 | 1.97273 | COTL1 | 0.0173498 | 1.87628 |
| S100A11 | 0.00305132 | 1.96806 | HSPB1 | 0.000119283 | 1.87343 |
| CTPS | 0.00736087 | 1.96543 | BRMS1L | 0.0346498 | 1.87094 |
| ACSS1 | 0.0184484 | 1.96488 | NFYB | 0.00202677 | 1.8689 |
| HNMT | 0.00761871 | 1.96127 | AP1M1 | 0.00307059 | 1.86845 |
| TG | 0.0107898 | 1.96093 | WEE1 | 0.00145312 | 1.8668 |
| AX747582 | 0.00845319 | 1.95636 | MTMR10 | 0.00716182 | 1.86373 |
| GRAMD1C | 0.00226138 | 1.95518 | C3orf43 | 0.0321532 | 1.86292 |
| OR2AG2 | 0.0162415 | 1.95096 | SSH2 | 0.00311066 | 1.86272 |
| SKP2 | 0.000752515 | 1.95061 | TMEM110 | 0.00841112 | 1.86215 |
| CNN1 | 0.0172085 | 1.94926 | ACTB | 0.0198031 | 1.85667 |
| CDH15 | 0.00260405 | 1.94704 | TST | 0.00656643 | 1.85402 |
| NOSTRIN | 0.0277534 | 1.9418 | TTC32 | 0.00535253 | 1.85333 |
| LIMS2 | 0.0211709 | 1.93969 | TRIM7 | 0.00482179 | 1.85086 |
| HMMR | 0.0141799 | 1.93876 | ITGBL1 | 0.00273311 | 1.84438 |
| RHOBTB3 | 0.00270591 | 1.93864 | CCNE2 | 0.032104 | 1.83894 |
| CDCA4 | 0.0148564 | 1.93506 | C9orf3 | 0.000526926 | 1.83891 |
| TMTC1 | 0.00778027 | 1.93196 | GPRC5B | 0.0202692 | 1.83612 |
| THC2539563 | 0.0165637 | 1.92263 | TRAM1 | 0.0074447 | 1.83573 |
| TIMM22 | 0.00145301 | 1.92005 | RGNEF | 0.0416197 | 1.83562 |
| MRM1 | 0.00286021 | 1.91949 | MMP19 | 0.0111424 | 1.83523 |
| YRDC | 0.0375096 | 1.91836 | LOC400743 | 0.0135742 | 1.83486 |
| POTEF | 0.00355557 | 1.91792 | UBL7 | 0.0245274 | 1.83452 |
| WDR1 | 0.00570827 | 1.91536 | PIK3C2A | 0.0187197 | 1.83449 |
| BANF1 | 0.00283209 | 1.91218 | CORO1B | 0.0452685 | 1.83428 |
| TMCO6 | 0.000575061 | 1.91053 | TCF19 | 0.0310745 | 1.83413 |
| LOC283663 | 0.0140178 | 1.90931 | TP53I11 | 0.00659414 | 1.83377 |
| NCAPH | 0.0212179 | 1.90562 | LRRC42 | 0.00157328 | 1.83296 |
| WDR37 | 0.0198291 | 1.90296 | HEG1 | 0.0123619 | 1.83219 |
| LOC100129269 | 0.0413582 | 1.90202 | STOM | 0.000282 | 1.83158 |
| RRAS2 | 0.00256184 | 1.90171 | EPHB6 | 0.00255392 | 1.83116 |
| SDHB | 0.00824247 | 1.90123 | SERPINA3 | 0.013681 | 1.82793 |
| NLN | 0.0209493 | 1.89853 | TGFBR2 | 0.000128245 | 1.82327 |
| BRIX1 | 0.0136274 | 1.89733 | OPN3 | 0.00906124 | 1.82318 |
| DCXR | 0.00131425 | 1.89688 | RRP9 | 0.00220878 | 1.82082 |
| TRAPPC1 | 0.00530354 | 1.89552 | FJX1 | 0.0189592 | 1.82023 |
| ASF1B | 0.0469889 | 1.89282 | PRICKLE3 | 0.0409922 | 1.81724 |
| SNAR-A3 | 0.0243274 | 1.89057 | GHR | 0.0279752 | 1.81717 |
| ATOH8 | 0.00349795 | 1.88994 | RASGRP2 | 0.0109952 | 1.81531 |
| LAMA2 | 0.0118164 | 1.88945 | PLA2G4E | 0.030434 | 1.81343 |
| MT1F | 0.00309801 | 1.88839 | SYPL2 | 0.0302298 | 1.81236 |
| TACC3 | 0.0102217 | 1.88794 | SEH1L | 0.010897 | 1.81144 |
| LOC100287322 | 0.00902287 | 1.8044 | MAP1D | 0.000839781 | 1.74591 |
| ZNF236 | 0.0353299 | 1.8036 | BC031250 | 0.0412106 | 1.744 |
| ATP1B1 | 0.017102 | 1.80204 | VCL | 0.0317248 | 1.74152 |
| SWAP70 | 0.00601783 | 1.80193 | UBASH3B | 0.012351 | 1.74036 |
| B3GNT2 | 0.0334527 | 1.79871 | ADAP1 | 0.0225462 | 1.73795 |
| ST6GALNAC2 | 0.023229 | 1.79789 | FBLN5 | 0.00390276 | 1.73776 |
| TUBB2A | 0.015036 | 1.79654 | TCEAL6 | 0.0034694 | 1.73589 |
| ANXA2 | 0.00206354 | 1.7958 | ENST00000390268 | 0.038455 | 1.73299 |
| UXT | 0.0114532 | 1.79375 | INMT | 0.0243677 | 1.73051 |
| ANGPT1 | 0.0403703 | 1.79187 | PRDX6 | 0.0449437 | 1.73049 |
| FERMT2 | 0.00244785 | 1.79174 | ZFP36 | 0.00078675 | 1.72922 |
| MYO1E | 0.0262112 | 1.79133 | C20orf134 | 0.00915441 | 1.72737 |
| FAM83H | 0.0116877 | 1.7882 | P704P | 0.0207662 | 1.72579 |
| KLF6 | 0.00638809 | 1.78657 | LOC100128934 | 0.0208209 | 1.72542 |
| DLK2 | 0.00350101 | 1.7864 | SMARCD2 | 0.00092234 | 1.72355 |
| NCL | 0.0117862 | 1.78619 | LOC644538 | 0.00754975 | 1.72277 |
| MTRR | 0.0117564 | 1.78587 | DONSON | 0.0126751 | 1.7221 |
| AFAP1L1 | 0.0177821 | 1.78496 | ENST00000434415 | 0.00520962 | 1.72199 |
| LOC92249 | 0.000703134 | 1.78488 | SNRPB | 0.0285952 | 1.72109 |
| KIAA1467 | 0.0121673 | 1.7841 | GSTT2B | 0.00788193 | 1.71968 |
| MRVI1 | 0.0123391 | 1.78339 | COL5A3 | 0.0130896 | 1.71903 |
| ZYX | 0.0468308 | 1.78334 | SCAF1 | 0.0196109 | 1.71662 |
| ITGA4 | 0.00513756 | 1.78021 | EARS2 | 0.0313925 | 1.71609 |
| LOC100290344 | 0.00155041 | 1.77948 | MFSD6 | 0.0161656 | 1.71476 |
| THC2715632 | 0.0124454 | 1.77828 | ROR1 | 0.00506866 | 1.71476 |
| MGLL | 0.0364153 | 1.77693 | ARSK | 2.29E-05 | 1.71455 |
| PUS7 | 0.0040995 | 1.77537 | APOOL | 0.000623268 | 1.71439 |
| STBD1 | 0.021483 | 1.77418 | C10orf114 | 0.0234086 | 1.71071 |
| SEC14L2 | 0.0127481 | 1.77342 | CDC42SE2 | 0.00277149 | 1.71053 |
| BF515046 | 0.0214467 | 1.76782 | F12 | 0.0433054 | 1.70921 |
| CCDC107 | 0.00458112 | 1.76646 | PKDCC | 0.00412631 | 1.70851 |
| CTHRC1 | 0.00325109 | 1.76522 | CCDC69 | 0.00833739 | 1.70729 |
| IRS2 | 4.84E-05 | 1.76474 | ANXA2P3 | 0.00434377 | 1.70558 |
| TMEM2 | 0.016153 | 1.76296 | TAS2R9 | 0.0452797 | 1.70384 |
| MFAP5 | 0.0356614 | 1.76125 | NID1 | 0.0219034 | 1.70331 |
| DA567289 | 0.0184802 | 1.7595 | ARSJ | 0.0327042 | 1.7027 |
| PDLIM1 | 0.0171536 | 1.75844 | AKAP7 | 0.00878199 | 1.70249 |
| SHBG | 0.0016997 | 1.7583 | CDK2AP2 | 0.0234804 | 1.70193 |
| TACC1 | 0.00582566 | 1.75689 | C1orf152 | 0.00119424 | 1.70175 |
| TPM2 | 0.00212428 | 1.75592 | SCAMP2 | 0.0146757 | 1.7017 |
| COQ2 | 0.0452233 | 1.75548 | ELN | 0.0214547 | 1.70075 |
| CHST2 | 0.0152059 | 1.755 | RSPO1 | 0.0343332 | 1.70073 |
| PPARG | 0.0262484 | 1.75222 | IL1F10 | 0.0181831 | 1.70026 |
| HIP1 | 0.0231741 | 1.74858 | MCM6 | 0.0049255 | 1.69953 |
| SYT2 | 0.0267923 | 1.74762 | LDLR | 0.0377244 | 1.69872 |
| IL18RAP | 0.00556959 | 1.74752 | ACER1 | 0.0150161 | 1.69818 |
| ARHGAP29 | 0.00878362 | 1.7473 | C7orf40 | 0.0137765 | 1.69793 |
| HIPK2 | 0.00966278 | 1.7471 | PRRG1 | 0.00591503 | 1.69748 |
| CNN2 | 0.00647049 | 1.747 | MYL12A | 0.0156914 | 1.69725 |
| ECE2 | 0.0209534 | 1.69643 | TPM1 | 0.00213395 | 1.6531 |
| MYL6 | 0.0117597 | 1.6956 | TLE1 | 0.00573661 | 1.65247 |
| PEBP4 | 0.0364478 | 1.69473 | OR6K2 | 0.0421191 | 1.64882 |
| TCEAL2 | 0.00862442 | 1.69264 | STON1 | 0.0195369 | 1.64803 |
| PARVB | 0.0271266 | 1.6916 | NAA10 | 0.00421202 | 1.64523 |
| UAP1 | 0.00442433 | 1.69151 | SPOCD1 | 0.00964886 | 1.64475 |
| PRPF3 | 0.0238776 | 1.69001 | SNAPC2 | 0.028432 | 1.6437 |
| PLSCR4 | 0.000556663 | 1.68971 | ENPP4 | 0.000663693 | 1.6429 |
| LOC643371 | 0.0132777 | 1.68945 | DUSP23 | 0.045555 | 1.6415 |
| LOC389333 | 0.0162535 | 1.68786 | FARSB | 0.00319796 | 1.64021 |
| TCIRG1 | 0.0136844 | 1.68759 | MUC1 | 0.00419683 | 1.6393 |
| FABP5 | 0.00419207 | 1.68682 | LOC100128714 | 0.000836864 | 1.639 |
| IGF2 | 0.00430152 | 1.68655 | FGF1 | 0.0434016 | 1.6388 |
| KLHDC3 | 0.0243576 | 1.68567 | HR | 0.0105812 | 1.63844 |
| ANO9 | 0.021372 | 1.68523 | CLTB | 0.00141911 | 1.63841 |
| FKBP1B | 0.00211799 | 1.68503 | MTSS1L | 0.00292838 | 1.63676 |
| AX746533 | 0.0395465 | 1.68485 | ADAM19 | 0.00985981 | 1.63592 |
| UBE2N | 0.00999596 | 1.68317 | AK131288 | 0.00651412 | 1.63472 |
| POTEE | 0.031172 | 1.68208 | OR2M7 | 0.029241 | 1.63449 |
| CENPVL1 | 0.0146573 | 1.67962 | SLC25A32 | 0.00479982 | 1.63413 |
| SRPX | 0.00036694 | 1.67958 | LRRC59 | 0.00615603 | 1.63396 |
| SHMT1 | 0.0280122 | 1.6767 | SLC16A3 | 0.0236032 | 1.63375 |
| POTEKP | 0.0352168 | 1.6765 | PALLD | 0.0120431 | 1.63368 |
| FAM114A2 | 0.0295041 | 1.67537 | HMHB1 | 0.0419993 | 1.63363 |
| DNAJC17 | 0.00170186 | 1.67395 | PHOSPHO2 | 0.0247257 | 1.63363 |
| CD3EAP | 0.00513222 | 1.67367 | NANOS1 | 0.0428206 | 1.63205 |
| OR10P1 | 0.0134752 | 1.67212 | MSRB3 | 0.00357306 | 1.63186 |
| BC018676 | 0.00151845 | 1.6721 | DCT | 0.00721748 | 1.63157 |
| C9orf29 | 0.0294436 | 1.67129 | DSTN | 0.00352243 | 1.6293 |
| KIF17 | 0.00089881 | 1.67089 | GRAMD3 | 0.0327261 | 1.62699 |
| DHCR24 | 0.0161305 | 1.66965 | FAM198B | 0.00097895 | 1.62566 |
| GPR173 | 0.0371352 | 1.66959 | CLDN7 | 0.0322532 | 1.62537 |
| SHOX | 0.00212326 | 1.66851 | RUNX2 | 0.0268564 | 1.62489 |
| PCGF5 | 0.00245105 | 1.66764 | LPIN3 | 0.0131145 | 1.62462 |
| ACTN1 | 0.031856 | 1.66748 | GCHFR | 0.0276693 | 1.6245 |
| CD14 | 0.00424562 | 1.66682 | CHST15 | 0.0367848 | 1.62196 |
| LSM12 | 0.00186415 | 1.66515 | KRTAP19-8 | 0.0208002 | 1.62194 |
| KBTBD11 | 0.00753394 | 1.664 | DKFZp667E0512 | 0.0341119 | 1.62155 |
| ENST00000374441 | 0.00490774 | 1.66385 | PIP4K2C | 0.0176893 | 1.62 |
| TSEN2 | 0.00079817 | 1.66345 | CSRP1 | 0.029417 | 1.61974 |
| RAI14 | 0.00725905 | 1.662 | LOC400950 | 0.0216949 | 1.61924 |
| KIAA0114 | 0.00321781 | 1.66087 | OIP5 | 0.0490564 | 1.61912 |
| CRHR1 | 0.0348277 | 1.66061 | TUBA1B | 0.0303507 | 1.61872 |
| SRPX2 | 0.00241227 | 1.6606 | TIMM16 | 0.0086793 | 1.61744 |
| ZRANB1 | 0.0239837 | 1.65782 | FLNA | 0.02978 | 1.61702 |
| C10orf96 | 0.0121685 | 1.65765 | PAPSS2 | 0.0375943 | 1.61627 |
| NPAS1 | 0.00104748 | 1.65599 | CGREF1 | 0.016036 | 1.61571 |
| COMTD1 | 0.029183 | 1.65516 | ANXA2P1 | 0.0195384 | 1.61496 |
| NRAS | 0.00106167 | 1.655 | SERTAD3 | 0.0146449 | 1.61403 |
| PNO1 | 0.0465145 | 1.61377 | SEMA7A | 0.00883937 | 1.57502 |
| SLC30A6 | 0.000340055 | 1.61364 | BARHL1 | 0.0389394 | 1.57497 |
| CYTH3 | 0.0353411 | 1.61233 | TMEM150A | 0.0183261 | 1.57445 |
| AK123701 | 0.0279208 | 1.61074 | C6orf150 | 0.00443606 | 1.57318 |
| PPP1R13L | 0.00364898 | 1.61034 | ATF6 | 0.0114363 | 1.57296 |
| AHCTF1 | 0.018066 | 1.61025 | CYP7B1 | 0.0120147 | 1.5722 |
| SCHIP1 | 0.00872129 | 1.60964 | CPNE7 | 0.0276006 | 1.57007 |
| REEP4 | 0.000543764 | 1.606 | TGIF1 | 0.0178419 | 1.57001 |
| ANXA6 | 0.0339389 | 1.60521 | ZHX3 | 4.46E-05 | 1.56993 |
| PRR21 | 0.0248492 | 1.6049 | AMOTL2 | 0.0244798 | 1.56989 |
| TLR4 | 0.0493724 | 1.60452 | EID3 | 0.0193207 | 1.56988 |
| COL4A2 | 0.00176503 | 1.60439 | GSTT2 | 0.00137581 | 1.5697 |
| SSB | 0.00617313 | 1.60385 | C5orf25 | 0.000714449 | 1.569 |
| COQ3 | 0.00687164 | 1.60221 | ACSL1 | 0.00946129 | 1.56861 |
| PPIAL4A | 0.0081133 | 1.6016 | TIPARP | 0.000641681 | 1.56846 |
| FKBP11 | 0.0163706 | 1.60132 | CBX8 | 0.0430133 | 1.56843 |
| ARL4A | 0.0302989 | 1.60103 | ARNT2 | 0.015852 | 1.56725 |
| ATP13A2 | 0.0139831 | 1.59907 | TNFRSF12A | 0.0182899 | 1.56607 |
| PVT1 | 0.00470682 | 1.5987 | LOC646821 | 0.0265603 | 1.56384 |
| KLK4 | 0.0402675 | 1.59748 | STAG3L1 | 0.00289827 | 1.56379 |
| USP4 | 0.0184042 | 1.59571 | AQP8 | 0.0249671 | 1.56347 |
| PI3 | 0.00274427 | 1.59518 | CD200 | 0.0323791 | 1.56275 |
| SH3PXD2B | 0.0141415 | 1.59259 | H2AFJ | 0.0333066 | 1.56207 |
| UCHL3 | 0.0110704 | 1.59221 | RBM14 | 0.000551019 | 1.55969 |
| MYBBP1A | 0.0301502 | 1.59204 | NOL6 | 0.0114674 | 1.55958 |
| RAD1 | 0.0110491 | 1.59007 | RASL10B | 0.0107211 | 1.55778 |
| LHX1 | 0.0471444 | 1.58945 | C1QTNF5 | 0.0315733 | 1.55643 |
| IMPAD1 | 0.00244333 | 1.58851 | UGP2 | 0.00902641 | 1.55609 |
| COL9A3 | 0.00837941 | 1.58652 | PDSS1 | 0.0316673 | 1.55577 |
| NOC3L | 0.00583487 | 1.58609 | EEF1E1 | 0.00844804 | 1.55479 |
| LOC401557 | 0.0314518 | 1.58577 | HLA-DPB2 | 0.0326049 | 1.55424 |
| KRTAP9L1 | 0.0296681 | 1.58468 | EYA2 | 0.0272598 | 1.55416 |
| REEP1 | 0.00535273 | 1.58398 | CYP1B1 | 0.00408749 | 1.55401 |
| CAV1 | 0.00317772 | 1.58333 | C8orf85 | 0.0140055 | 1.55387 |
| EMP1 | 0.0344204 | 1.5828 | PTPDC1 | 0.00293183 | 1.55347 |
| CLOCK | 0.000948691 | 1.5826 | SGK269 | 0.0157111 | 1.55303 |
| KANK1 | 0.0308752 | 1.58206 | RRAS | 0.00576408 | 1.55272 |
| TMOD2 | 0.0217739 | 1.58025 | UBR3 | 0.0175791 | 1.55249 |
| INHBA | 0.0185378 | 1.57967 | HHEX | 0.0117605 | 1.5524 |
| SLC35D1 | 0.00461209 | 1.57963 | VKORC1L1 | 0.00456739 | 1.55211 |
| BCL7A | 0.0212591 | 1.57923 | PTPLA | 0.00786451 | 1.54888 |
| EIF5A | 0.0238916 | 1.57799 | MPP3 | 0.0390802 | 1.54885 |
| DHX35 | 0.00544215 | 1.5779 | RAB5B | 0.0120912 | 1.54728 |
| MSI1 | 0.00250336 | 1.57778 | CR615613 | 0.0251841 | 1.54671 |
| ILK | 0.0227481 | 1.5769 | SGTB | 0.0169976 | 1.54639 |
| SLC19A2 | 0.0336608 | 1.57651 | NCRNA00181 | 0.011972 | 1.54357 |
| AGPAT9 | 0.0457566 | 1.57627 | DIO2 | 0.0036974 | 1.54344 |
| GCM1 | 0.0245097 | 1.57604 | NOP2 | 0.0178898 | 1.54297 |
| TEAD3 | 0.00843151 | 1.57519 | ENST00000395453 | 0.027586 | 1.54276 |
| STC2 | 0.00428328 | 1.54241 | ZNF485 | 0.0316016 | 1.52082 |
| DPM2 | 0.00915178 | 1.54166 | RUNX1 | 0.0461004 | 1.52043 |
| OR8B8 | 0.0196988 | 1.54159 | FAM127B | 0.0339392 | 1.51995 |
| ENST00000342688 | 0.00519207 | 1.54061 | TWIST2 | 0.00200376 | 1.51932 |
| ZBTB7A | 0.00788696 | 1.53949 | NAP1L1 | 0.0121582 | 1.51875 |
| ZDHHC12 | 0.00684217 | 1.53934 | TCTE1 | 0.00719065 | 1.51819 |
| MPI | 0.0238554 | 1.53787 | SH3GL1P3 | 0.0234892 | 1.5177 |
| ZWINT | 0.0326763 | 1.53679 | RAD51 | 0.038863 | 1.51761 |
| SP140L | 0.00290213 | 1.53648 | SFPQ | 0.000477149 | 1.51678 |
| D13069 | 0.00136304 | 1.53621 | ANG | 0.00971459 | 1.51637 |
| LOC401561 | 0.00702947 | 1.53594 | IGF2BP2 | 1.69E-05 | 1.51616 |
| CABIN1 | 0.00113746 | 1.53584 | C7orf51 | 0.0468031 | 1.51615 |
| ACTR3 | 0.018905 | 1.53539 | IRS1 | 0.0147083 | 1.51522 |
| ADAMTS9 | 0.0182146 | 1.53538 | LOC340515 | 0.0175562 | 1.51522 |
| MED8 | 0.00138042 | 1.535 | REG3A | 0.0306929 | 1.51503 |
| NAA11 | 0.00473761 | 1.53474 | BLM | 0.0474108 | 1.5138 |
| URB2 | 0.001694 | 1.53426 | CHCHD7 | 0.00481288 | 1.51368 |
| LOC344382 | 0.0182254 | 1.53405 | FAM166A | 0.0205222 | 1.51338 |
| INHBC | 0.00960291 | 1.53377 | REEP3 | 0.0446637 | 1.5132 |
| LCE1A | 0.00423117 | 1.53363 | TNFSF13 | 0.0360986 | 1.51284 |
| TGFB1I1 | 0.00153039 | 1.53354 | FN3K | 0.00428976 | 1.51276 |
| GCAT | 0.0438546 | 1.53285 | DDX21 | 0.0144562 | 1.51194 |
| ENST00000377039 | 0.0358711 | 1.53251 | SFRS7 | 0.0389748 | 1.51147 |
| LOC100009676 | 0.021047 | 1.53169 | PTS | 0.0261883 | 1.51129 |
| UFSP1 | 0.00677257 | 1.53131 | ENST00000399730 | 0.00474636 | 1.50957 |
| CCND3 | 0.00263342 | 1.53086 | GRRP1 | 0.0384513 | 1.50939 |
| C7orf11 | 0.00371683 | 1.53067 | SAR1B | 0.0164026 | 1.50891 |
| HPDL | 0.0359797 | 1.53031 | ENST00000366413 | 0.0260504 | 1.50808 |
| CFLAR | 0.0157431 | 1.53002 | NDUFS3 | 0.0251057 | 1.50776 |
| OR5L2 | 0.00382358 | 1.52988 | LOC646576 | 0.0473773 | 1.50687 |
| PEA15 | 0.0158775 | 1.52966 | SAMD4A | 0.00270771 | 1.50563 |
| SGMS2 | 0.0273588 | 1.52934 | SBDS | 0.000455425 | 1.50489 |
| ENST00000376775 | 0.0351426 | 1.52884 | LOC128322 | 0.00954339 | 1.50443 |
| CDA | 2.40E-06 | 1.52856 | TJP2 | 0.00470322 | 1.50322 |
| SELS | 0.00793408 | 1.52767 | AMD1 | 0.0163402 | 1.5032 |
| SPTBN2 | 0.00634065 | 1.52673 | ERGIC1 | 0.0131549 | 1.50037 |
| RABL3 | 0.0292641 | 1.52611 | DUS2L | 0.0306426 | 1.50003 |
| PTPN18 | 0.00392213 | 1.52604 | SVIL | 3.91E-05 | -1.50118 |
| TAF7 | 0.0244297 | 1.52481 | ENST00000371488 | 0.018129 | -1.50177 |
| PEMT | 0.0286748 | 1.52465 | LOC401286 | 0.0406118 | -1.50186 |
| RNGTT | 0.00267116 | 1.52432 | PGPEP1 | 0.00642076 | -1.50262 |
| MRPL15 | 0.00598968 | 1.52378 | LOC84989 | 0.0255584 | -1.50326 |
| PRDM13 | 0.00945784 | 1.52289 | DSTYK | 9.62E-06 | -1.50372 |
| TXNIP | 0.0110062 | 1.52264 | CDYL | 0.0144349 | -1.50388 |
| LOC400236 | 0.0432812 | 1.52218 | CLU | 0.0117427 | -1.50485 |
| TTC13 | 0.021895 | 1.52208 | DZIP1 | 0.0104316 | -1.50724 |
| FAM199X | 0.0212841 | 1.52172 | KLRG1 | 0.00584876 | -1.50774 |
| RHOB | 0.0354701 | 1.52138 | ARHGEF2 | 0.00433198 | -1.50825 |
| TCEAL5 | 0.0154601 | 1.52098 | JMJD7-PLA2G4B | 0.0081886 | -1.51007 |
| TRIM22 | 0.00519648 | -1.51009 | PAN3 | 0.0467904 | -1.53088 |
| SPATA4 | 0.0411625 | -1.51087 | PARP14 | 0.00361116 | -1.53115 |
| FRAT1 | 0.0170407 | -1.51164 | CBR3 | 0.0313695 | -1.53126 |
| PSIP1 | 0.0214491 | -1.51176 | SLC35E2 | 0.00766206 | -1.53148 |
| UNK | 0.00355972 | -1.51186 | EGFL8 | 0.0162427 | -1.53151 |
| PBX2 | 0.0399724 | -1.51247 | C9orf140 | 0.00410175 | -1.53226 |
| NFKBIE | 0.0149794 | -1.51322 | PNPLA7 | 0.0215441 | -1.53357 |
| CCDC102A | 0.0326922 | -1.51323 | PAFAH1B3 | 0.0206772 | -1.53383 |
| ENST00000432854 | 0.00592678 | -1.51355 | FAM107B | 0.0301075 | -1.53401 |
| C20orf177 | 0.0110692 | -1.51368 | TMEM121 | 0.00322258 | -1.53422 |
| SNX30 | 0.0259454 | -1.51489 | LNP1 | 0.0368891 | -1.53479 |
| NNAT | 0.04094 | -1.51674 | OR4A15 | 0.0372018 | -1.53544 |
| ZMAT3 | 0.0295509 | -1.51674 | TP53BP1 | 0.00357083 | -1.53581 |
| ARID5A | 0.0188059 | -1.51712 | DST | 0.0182532 | -1.53628 |
| LOC100132774 | 0.0129553 | -1.51754 | DPP4 | 0.00596302 | -1.53686 |
| LOC100133131 | 0.00438744 | -1.51788 | ZNF780B | 0.0053232 | -1.53694 |
| SLC16A4 | 0.0245246 | -1.518 | GPT2 | 0.0324714 | -1.5375 |
| TCF4 | 0.00422215 | -1.51811 | RPS6KA3 | 0.00285817 | -1.53772 |
| PSD3 | 0.0212434 | -1.51812 | CTSLL2 | 0.0220518 | -1.53788 |
| PRKACB | 0.00126809 | -1.5183 | C1orf96 | 0.00799625 | -1.53825 |
| LOC100130009 | 0.00414006 | -1.51882 | DHFRL1 | 0.00437084 | -1.53929 |
| SCARA3 | 0.00126832 | -1.51937 | C6orf225 | 0.043217 | -1.53997 |
| ZNF74 | 0.0418136 | -1.5196 | LIMA1 | 0.00164674 | -1.54037 |
| SLC45A3 | 0.0259792 | -1.51975 | GRAMD4 | 0.00123944 | -1.54154 |
| CTNNBIP1 | 0.000725854 | -1.52066 | FANCE | 0.00215588 | -1.5421 |
| UTS2R | 0.0194121 | -1.52094 | LRRC2 | 0.00240516 | -1.5426 |
| ENST00000439203 | 0.000861267 | -1.52145 | PELI1 | 0.0270355 | -1.54307 |
| INSIG1 | 0.0327888 | -1.52223 | HCFC1R1 | 0.00402001 | -1.54366 |
| TTLL8 | 0.0119498 | -1.52252 | LOC100128416 | 0.0384879 | -1.54451 |
| BAGE4 | 0.00793398 | -1.52366 | TIA1 | 0.0232977 | -1.54501 |
| GAMT | 0.0020288 | -1.52378 | USP27X | 0.038469 | -1.54513 |
| C9orf7 | 0.00158852 | -1.52455 | PIK3IP1 | 0.00770499 | -1.54543 |
| MXI1 | 0.00832321 | -1.525 | AUTS2 | 0.010285 | -1.54547 |
| ARHGEF6 | 0.0310599 | -1.52589 | SLC9A9 | 0.0108561 | -1.54589 |
| KLF11 | 0.00235205 | -1.52623 | ZMIZ1 | 0.00322675 | -1.54876 |
| TGFBR3 | 0.0243335 | -1.5267 | PLK2 | 0.0195957 | -1.5492 |
| CRBN | 0.0141834 | -1.52675 | ZNRF1 | 0.00954571 | -1.55056 |
| WWC2 | 0.0451787 | -1.52695 | STAP2 | 0.00308432 | -1.55117 |
| ACAD11 | 0.0476931 | -1.52701 | FES | 0.0247493 | -1.55169 |
| EPHB2 | 0.0168057 | -1.52702 | CARD17 | 0.0322264 | -1.5533 |
| SESN2 | 0.00128195 | -1.52719 | RFX7 | 0.0334087 | -1.55383 |
| NISCH | 0.0268881 | -1.52724 | HMGB3L1 | 0.0115716 | -1.55489 |
| KIAA0495 | 0.0382779 | -1.52807 | DDAH2 | 7.24E-05 | -1.55491 |
| OCIAD2 | 0.0145437 | -1.52815 | PGM5 | 0.0217452 | -1.55499 |
| ADNP | 0.00143483 | -1.52851 | FAM125B | 0.00439971 | -1.55544 |
| BICC1 | 0.00733564 | -1.52852 | LOC283861 | 0.0216603 | -1.55704 |
| OSBPL7 | 0.00569432 | -1.52998 | MOXD1 | 0.026759 | -1.55709 |
| FHOD3 | 0.026902 | -1.53069 | LIPT1 | 0.00546301 | -1.55854 |
| FYCO1 | 0.0129197 | -1.53088 | C6orf134 | 0.00811826 | -1.55858 |
| FAM171A1 | 0.000119603 | -1.55906 | CIC | 0.00282024 | -1.58387 |
| LOC441795 | 0.0121469 | -1.55918 | SACS | 0.00191987 | -1.58394 |
| IGF2R | 0.00105074 | -1.55968 | PRNP | 0.00141919 | -1.58435 |
| SSX2IP | 0.0464588 | -1.55989 | HSPA12A | 0.0109366 | -1.58531 |
| TSHZ3 | 0.00713993 | -1.5604 | FZD1 | 0.0252886 | -1.5856 |
| CAND2 | 0.0274221 | -1.56079 | AX747640 | 0.00455084 | -1.58625 |
| LOC285629 | 0.0107335 | -1.56239 | TBC1D2B | 0.00222875 | -1.58926 |
| HGF | 0.00067535 | -1.56261 | RN5-8S1 | 0.000105192 | -1.59011 |
| NEK10 | 0.0101658 | -1.56262 | ARHGAP22 | 0.0183132 | -1.59129 |
| C6orf154 | 0.0446415 | -1.56294 | CDKN1A | 0.0118619 | -1.59219 |
| PTPN13 | 0.00818166 | -1.5656 | SPEG | 0.0322934 | -1.59256 |
| Sep-05 | 0.00748971 | -1.56627 | EXT1 | 0.0124141 | -1.59257 |
| OAF | 0.0179696 | -1.56655 | NCRNA00219 | 0.00648519 | -1.5932 |
| EPAS1 | 0.00121363 | -1.56688 | KIAA0895L | 0.0279553 | -1.59322 |
| PGAP1 | 0.00732748 | -1.56794 | EYA4 | 0.0172744 | -1.59475 |
| VPS8 | 0.0466266 | -1.56849 | PTPRU | 0.000925544 | -1.59492 |
| PABPC4L | 0.0200126 | -1.56891 | ZNF561 | 0.0120664 | -1.59584 |
| JMY | 0.00957186 | -1.56926 | CFB | 0.0492871 | -1.59601 |
| MARCKS | 0.0220441 | -1.5695 | HIST1H3J | 0.0285744 | -1.59676 |
| PCSK4 | 0.031863 | -1.56987 | C2orf60 | 0.0015856 | -1.59832 |
| CPZ | 0.0060581 | -1.57003 | TMEFF2 | 0.0168481 | -1.59894 |
| BACH2 | 0.0386525 | -1.57006 | LRRC49 | 0.00267242 | -1.60017 |
| NR2F1 | 0.0252862 | -1.57035 | PTEN | 0.013739 | -1.60028 |
| C16orf74 | 0.0033656 | -1.57048 | FGF7 | 0.000138676 | -1.60135 |
| LOC554202 | 0.00469693 | -1.57097 | CCDC142 | 0.0156004 | -1.60154 |
| EYA1 | 0.030475 | -1.57168 | SLC4A4 | 0.0385978 | -1.60178 |
| RFX2 | 0.00724667 | -1.57231 | SLC25A29 | 0.000582752 | -1.60207 |
| ZBTB2 | 0.0272753 | -1.57372 | IRF2BP2 | 0.0281924 | -1.60393 |
| SLC27A1 | 0.000601986 | -1.57382 | C20orf108 | 0.000778739 | -1.604 |
| NTNG2 | 0.0457537 | -1.57412 | ANKDD1A | 0.00119112 | -1.6064 |
| PLEKHN1 | 0.0381408 | -1.57421 | BCL9 | 0.0212728 | -1.60678 |
| TIGD1 | 0.00327985 | -1.57507 | XPC | 0.0384741 | -1.60856 |
| C5orf41 | 0.00619559 | -1.57538 | MASTL | 0.0423368 | -1.60881 |
| HIST1H3A | 0.00412538 | -1.57599 | LRRN4CL | 0.00487135 | -1.60944 |
| LOC375010 | 0.0383873 | -1.57721 | BCL2 | 0.0159714 | -1.61054 |
| THAP11 | 0.010318 | -1.57746 | KCNE4 | 0.0201001 | -1.61136 |
| HIST1H4B | 0.0118353 | -1.57778 | GLIS2 | 0.0114409 | -1.61181 |
| LOC286161 | 0.0284388 | -1.57806 | LONP1 | 0.0433688 | -1.61228 |
| GATAD2B | 0.0226438 | -1.57909 | ABCA7 | 0.00567939 | -1.61241 |
| CCR10 | 0.00229571 | -1.57911 | TCF3 | 0.036804 | -1.61336 |
| ERV3 | 0.0368821 | -1.57935 | HIST1H3E | 0.0233539 | -1.61351 |
| IQCD | 0.039425 | -1.57941 | LOC100287428 | 0.0151964 | -1.61445 |
| C14orf37 | 0.0492402 | -1.57951 | AK058117 | 0.0352799 | -1.61696 |
| TMEM66 | 0.01153 | -1.57965 | HECTD1 | 0.00289452 | -1.61715 |
| LOC100170939 | 0.0219398 | -1.57981 | ENST00000367596 | 0.01976 | -1.61751 |
| PCSK5 | 0.0103333 | -1.58082 | TUBA4A | 0.00111482 | -1.61849 |
| SASH1 | 0.000198955 | -1.58196 | TOB1 | 0.000268462 | -1.62097 |
| LOC388242 | 0.0115597 | -1.58197 | AKD1 | 0.00166826 | -1.62342 |
| SLC37A2 | 0.0338481 | -1.582 | PMEPA1 | 0.0225467 | -1.62404 |
| BC043411 | 0.00463014 | -1.62515 | MAST4 | 0.000717315 | -1.65826 |
| SOD2 | 0.0109965 | -1.6255 | KRTAP4-11 | 0.0335412 | -1.65855 |
| C7orf61 | 0.00337805 | -1.62561 | HSPB8 | 0.00368304 | -1.65872 |
| SH3BGR | 0.0245866 | -1.62585 | C8orf47 | 0.0133066 | -1.66033 |
| CD44 | 0.00616166 | -1.62596 | DAB2IP | 0.0318262 | -1.66035 |
| HIST1H4K | 0.0158534 | -1.62666 | HIST2H4B | 0.0129093 | -1.66159 |
| PPL | 0.0396414 | -1.62749 | PER3 | 0.0290996 | -1.66177 |
| RAPGEF6 | 0.0499704 | -1.62765 | C8orf31 | 0.0211327 | -1.662 |
| IFI16 | 0.00541607 | -1.62783 | RHOJ | 0.0338931 | -1.66223 |
| SNHG7 | 0.0188353 | -1.62897 | C4orf46 | 0.0307614 | -1.66389 |
| CCNG1 | 0.013129 | -1.63104 | TSHZ1 | 0.0283001 | -1.66502 |
| PTPRK | 0.00463236 | -1.63175 | NBEA | 0.0017485 | -1.66531 |
| CR620599 | 0.000494353 | -1.63331 | FRMD4A | 0.0354197 | -1.66574 |
| SOCS1 | 0.00656085 | -1.63468 | PGF | 0.0148908 | -1.66606 |
| CA11 | 0.0232012 | -1.63497 | CASK | 0.0059835 | -1.66618 |
| BTG2 | 0.00872136 | -1.63624 | COLEC12 | 0.00566187 | -1.66626 |
| NAALADL2 | 0.00430369 | -1.6368 | GALNT12 | 0.0414699 | -1.66682 |
| RPA4 | 0.0185263 | -1.63774 | PBXIP1 | 0.0114431 | -1.66771 |
| AGAP1 | 0.0029188 | -1.64008 | CFP | 0.000356378 | -1.66785 |
| AX747706 | 0.0042293 | -1.64027 | PCDHB9 | 0.0282593 | -1.66842 |
| C21orf49 | 0.00910901 | -1.64131 | CARD10 | 0.0262575 | -1.66969 |
| SPATA13 | 0.00279529 | -1.64136 | PTPRS | 0.00361604 | -1.66977 |
| LOXL1 | 0.0224028 | -1.64156 | XAF1 | 0.0117507 | -1.67157 |
| ACCN2 | 0.0059297 | -1.64164 | KIAA1549 | 0.0163473 | -1.67198 |
| KRBA2 | 0.026965 | -1.64187 | KCNAB3 | 0.0381156 | -1.67238 |
| C21orf66 | 0.00185886 | -1.64204 | METRNL | 0.000238882 | -1.67329 |
| ZNF667 | 0.00527452 | -1.64351 | CEP57 | 0.00714972 | -1.67499 |
| PGBD2 | 0.0168251 | -1.64447 | ANGPTL6 | 0.043869 | -1.67641 |
| LOC729175 | 0.00211095 | -1.64581 | DTWD1 | 0.00758613 | -1.67643 |
| AGT | 0.027293 | -1.64589 | GRASP | 0.00193064 | -1.67698 |
| TBX3 | 0.0172112 | -1.64615 | DYNLRB2 | 0.0249205 | -1.67826 |
| SCARNA9 | 0.00113747 | -1.64706 | MAP1A | 0.0123902 | -1.67888 |
| IFT88 | 0.00195007 | -1.64851 | FAM46A | 0.0405706 | -1.67908 |
| IFIT3 | 0.00695599 | -1.64925 | CENPL | 0.0161494 | -1.67969 |
| IFI30 | 0.00140305 | -1.64944 | AHCYL2 | 0.0175128 | -1.68008 |
| P4HA2 | 8.84E-05 | -1.65031 | ADAL | 0.0172775 | -1.68042 |
| LOC100132891 | 0.0117061 | -1.6522 | NDRG3 | 0.0213009 | -1.68045 |
| UCN | 0.0135158 | -1.65232 | TNIK | 0.0298829 | -1.68131 |
| LOC644925 | 0.0017931 | -1.65258 | NFE2L3 | 0.0145034 | -1.68365 |
| ADSSL1 | 0.042551 | -1.65277 | NEK11 | 0.0299512 | -1.68536 |
| SYNJ2 | 0.00265125 | -1.65338 | SLC43A3 | 0.0208433 | -1.68536 |
| MFAP4 | 0.00772648 | -1.65375 | KLF3 | 0.0179288 | -1.68711 |
| PHACTR1 | 0.0114834 | -1.65397 | VAMP4 | 0.0047297 | -1.68747 |
| NAF1 | 0.0186522 | -1.65402 | GPRASP2 | 0.00117835 | -1.6894 |
| PLCB1 | 0.00486583 | -1.65479 | ZCCHC14 | 0.00195396 | -1.69103 |
| BTN3A1 | 0.0133041 | -1.65555 | SP4 | 0.0210173 | -1.69166 |
| TBX4 | 0.0105486 | -1.65566 | CIDEA | 0.00620517 | -1.69308 |
| CLSTN3 | 0.00490672 | -1.65616 | TNFRSF10B | 0.00269714 | -1.69333 |
| FAM20A | 0.0120662 | -1.65618 | CYS1 | 0.00652154 | -1.69385 |
| ZMYM3 | 0.006019 | -1.69545 | LOC100291791 | 0.0212388 | -1.75355 |
| ANKRD36 | 0.0172586 | -1.69602 | PRR3 | 0.0274573 | -1.75369 |
| LHFPL2 | 0.00110919 | -1.69722 | ZNF497 | 0.00480268 | -1.75486 |
| FAM155A | 0.00346109 | -1.69912 | MKL2 | 0.0175421 | -1.75511 |
| ARHGEF9 | 0.0041324 | -1.69967 | SCN2A | 0.0101107 | -1.75649 |
| RNF146 | 0.00551771 | -1.69985 | NAB1 | 0.00264615 | -1.7566 |
| LOC729595 | 0.0330492 | -1.70081 | PRR19 | 0.0113106 | -1.75719 |
| BATF2 | 0.0353445 | -1.70138 | VPS37D | 0.00121596 | -1.75867 |
| MYST2 | 0.0356337 | -1.70279 | SORL1 | 0.0224045 | -1.7594 |
| SOX9 | 0.0398713 | -1.70363 | MTHFR | 0.00074491 | -1.76002 |
| THC2620530 | 0.0178753 | -1.70371 | SOCS2 | 0.0293658 | -1.76019 |
| LOC100049716 | 0.0151159 | -1.70412 | TMEFF1 | 0.00745907 | -1.76084 |
| SLC5A12 | 0.0490626 | -1.70456 | RGS10 | 0.0165217 | -1.76131 |
| TEF | 0.024159 | -1.70601 | ZNF653 | 0.0242651 | -1.76346 |
| PFN4 | 0.00382213 | -1.70605 | FAM161A | 0.00469633 | -1.76665 |
| SEC31B | 0.000431119 | -1.70807 | PRTFDC1 | 0.00643595 | -1.7693 |
| NINL | 0.00421214 | -1.70862 | PURG | 0.0397532 | -1.77074 |
| ING4 | 0.00133649 | -1.71 | SELENBP1 | 0.016457 | -1.7712 |
| EDA2R | 0.00952513 | -1.71049 | STARD10 | 0.00278286 | -1.77196 |
| C3orf65 | 0.0024727 | -1.71099 | TIMP3 | 0.00502799 | -1.77224 |
| MSX2P1 | 0.00712371 | -1.71314 | LOC286367 | 0.0132135 | -1.77311 |
| CARD16 | 0.0202562 | -1.7135 | KRTAP10-5 | 0.017222 | -1.7738 |
| THC2655610 | 0.0036342 | -1.71357 | RASGEF1A | 0.0370069 | -1.77429 |
| C20orf132 | 0.0321411 | -1.71594 | ANKIB1 | 0.0140934 | -1.77583 |
| BAI2 | 0.00268282 | -1.7167 | TMEM88 | 0.0434898 | -1.77783 |
| RASSF5 | 0.0283061 | -1.71883 | FCGR2A | 0.0441334 | -1.77799 |
| KRTAP12-1 | 0.0136459 | -1.71975 | SHC2 | 0.00740202 | -1.77841 |
| PPAP2C | 0.0107034 | -1.72071 | SOCS3 | 0.0226194 | -1.77916 |
| LOC100130819 | 0.00473786 | -1.72083 | PDE4D | 0.0275595 | -1.77955 |
| RALGDS | 3.43E-05 | -1.72118 | IFIT2 | 0.00668926 | -1.78029 |
| CASP1 | 0.0249651 | -1.72716 | LIF | 0.0221744 | -1.78151 |
| CAMK4 | 0.0449033 | -1.72745 | SERPINE2 | 0.0027427 | -1.78308 |
| LUZP1 | 0.0281258 | -1.72824 | MCM7 | 0.00708165 | -1.78447 |
| SSC5D | 0.00873221 | -1.72928 | MYLIP | 0.0372086 | -1.78476 |
| POU6F1 | 0.0331716 | -1.72947 | DLG3 | 0.00784381 | -1.78483 |
| L3MBTL | 0.0425477 | -1.73008 | FAM129A | 0.0186803 | -1.78573 |
| ENST00000380683 | 0.0305764 | -1.73058 | PTPN4 | 0.013027 | -1.78616 |
| IFI44 | 0.00135842 | -1.734 | C20orf96 | 0.0283764 | -1.78686 |
| PRKCE | 0.0149573 | -1.7389 | RIBC1 | 0.0379209 | -1.78741 |
| POU2F1 | 0.0011445 | -1.73912 | JMJD7 | 0.0107785 | -1.78951 |
| TRIM5 | 0.000905955 | -1.7399 | PHKG2 | 0.000605278 | -1.79094 |
| IFFO1 | 0.000895382 | -1.74309 | HEXIM2 | 0.00687816 | -1.7913 |
| LOC153684 | 0.00318901 | -1.74384 | SNX21 | 0.000333609 | -1.79147 |
| MANBA | 0.00149642 | -1.74444 | STRN3 | 0.00806945 | -1.79592 |
| GYPC | 0.00371899 | -1.74593 | HIST1H2AE | 0.0189585 | -1.79718 |
| TNFRSF14 | 0.00281423 | -1.74941 | FOXP2 | 0.00878485 | -1.79894 |
| TCAP | 0.0444795 | -1.74962 | RBP1 | 0.0220172 | -1.79933 |
| COL6A6 | 0.0130339 | -1.74985 | SLC8A1 | 0.00987762 | -1.80136 |
| PIP5KL1 | 0.0193506 | -1.75202 | GPR162 | 0.000634474 | -1.80235 |
| ATF5 | 0.00187252 | -1.80327 | C4orf38 | 0.0491376 | -1.85925 |
| FLJ10357 | 0.00515077 | -1.80358 | TMEM200A | 0.000813525 | -1.86001 |
| EFCAB7 | 0.00849823 | -1.80586 | PPAP2A | 0.0157463 | -1.86252 |
| LOC100129365 | 0.0253237 | -1.80771 | NUMA1 | 0.0480917 | -1.86358 |
| HBP1 | 6.84E-05 | -1.80827 | ASPRV1 | 0.0114617 | -1.86435 |
| PARP11 | 0.0258225 | -1.81082 | SULF2 | 0.00131374 | -1.86519 |
| GALM | 0.003476 | -1.81111 | MAMSTR | 0.0316064 | -1.86552 |
| JMJD1C | 0.00409768 | -1.81133 | C14orf79 | 0.0204964 | -1.86717 |
| TCEAL7 | 0.000978636 | -1.81202 | POPDC2 | 0.0164453 | -1.86739 |
| VLDLR | 0.0107936 | -1.81259 | CSDC2 | 0.0323883 | -1.86758 |
| LRIG1 | 0.00421353 | -1.81332 | TRIM2 | 0.00120328 | -1.87009 |
| SERTAD2 | 8.93E-06 | -1.81362 | TMEM132A | 0.00860836 | -1.87226 |
| LYZL1 | 0.0140937 | -1.81371 | LOC282997 | 0.000162601 | -1.87541 |
| FNDC1 | 0.0124359 | -1.81688 | ARL4C | 0.00639056 | -1.87693 |
| OSBPL3 | 0.00277281 | -1.81795 | CPEB1 | 0.00159968 | -1.8786 |
| ULK1 | 0.00718744 | -1.81916 | ZBED3 | 0.00796132 | -1.88414 |
| PYROXD2 | 0.00762256 | -1.82146 | ZNF710 | 0.0360452 | -1.88414 |
| GPC2 | 0.0261739 | -1.8215 | PITX1 | 0.0195004 | -1.88446 |
| CD248 | 0.00174394 | -1.82167 | CD86 | 0.00242742 | -1.88519 |
| DDIT3 | 0.000237384 | -1.82169 | GALNTL1 | 0.0096522 | -1.88647 |
| ENST00000395936 | 0.0260157 | -1.82247 | CCDC74B | 0.00119343 | -1.88889 |
| FLJ90757 | 0.000294096 | -1.8225 | ABI3BP | 0.0220532 | -1.88936 |
| ANK2 | 0.0111876 | -1.82602 | PDCD4 | 0.0170675 | -1.89019 |
| FLYWCH1 | 0.00494036 | -1.82749 | GPR125 | 0.0360252 | -1.89059 |
| ROBO1 | 0.00760105 | -1.83005 | RP1-21O18.1 | 0.00975741 | -1.89328 |
| HIVEP2 | 6.07E-05 | -1.83162 | COL15A1 | 0.0295365 | -1.89335 |
| ZNF154 | 0.0363896 | -1.83166 | AK074144 | 0.0223447 | -1.89441 |
| SPATA18 | 0.00511123 | -1.83168 | C13orf33 | 0.0305718 | -1.89452 |
| AX747437 | 0.0289977 | -1.8326 | AHNAK2 | 0.0049417 | -1.89616 |
| CPAMD8 | 0.0227381 | -1.83279 | PFKFB3 | 0.00230741 | -1.89647 |
| GXYLT2 | 0.0120212 | -1.83413 | PELI2 | 0.000242001 | -1.89671 |
| LIG1 | 0.00731637 | -1.8349 | RAVER2 | 0.0260669 | -1.89701 |
| TMEM37 | 0.0189832 | -1.83492 | LOC100128163 | 0.040367 | -1.89801 |
| RN18S1 | 0.00160088 | -1.83672 | SLURP1 | 0.0199161 | -1.89843 |
| BEND6 | 0.00650894 | -1.83724 | FAM161B | 0.0294612 | -1.89988 |
| ASGR1 | 0.0128841 | -1.83878 | CAPS2 | 0.0472048 | -1.90106 |
| NR3C1 | 0.000498005 | -1.84159 | TCF7 | 0.00366817 | -1.90161 |
| EFR3B | 0.0157715 | -1.84229 | ST6GAL1 | 0.0137812 | -1.90173 |
| MIR155HG | 0.0056669 | -1.84519 | SPI1 | 0.0370844 | -1.90427 |
| C11orf20 | 0.020531 | -1.84819 | CHADL | 0.00374819 | -1.90464 |
| CCL15 | 0.0332264 | -1.84911 | PLCL2 | 0.00842468 | -1.90509 |
| MGC2848 | 0.0303144 | -1.84982 | CCDC85C | 0.000536567 | -1.90607 |
| HIST1H4I | 0.0108644 | -1.85003 | CLIC2 | 0.00280036 | -1.90808 |
| SEMA5A | 0.000115808 | -1.85342 | VEGFA | 7.80E-05 | -1.90829 |
| PKIA | 0.00511557 | -1.85379 | MALL | 0.0265984 | -1.90837 |
| PRKG1 | 0.00150954 | -1.85582 | RASA4 | 0.0154752 | -1.91052 |
| MCPH1 | 0.0178924 | -1.85766 | BCL2L11 | 0.0182161 | -1.91192 |
| ALDH3A2 | 0.0325662 | -1.85835 | LARP6 | 0.00148867 | -1.91759 |
| MX2 | 0.043301 | -1.85847 | ANKRD10 | 0.0108932 | -1.9192 |
| FAM65B | 0.0455055 | -1.91935 | CLN8 | 0.00177106 | -2.00272 |
| COL21A1 | 0.0404951 | -1.91941 | SOBP | 0.00246175 | -2.00551 |
| C3orf58 | 0.0405864 | -1.92104 | KIAA1683 | 0.0015688 | -2.00702 |
| SDCBP2 | 0.0279108 | -1.92118 | TCP11L2 | 0.0100366 | -2.00873 |
| PPP1R1B | 0.0368319 | -1.922 | NT5E | 0.00138952 | -2.01248 |
| PHLDB3 | 0.00452662 | -1.92721 | C2orf84 | 0.017976 | -2.01651 |
| C10orf41 | 0.00603495 | -1.93223 | PLXNC1 | 0.00731964 | -2.01663 |
| C15orf51 | 0.000213284 | -1.93515 | LOC730058 | 0.0331972 | -2.02103 |
| JUN | 0.00118891 | -1.93669 | ADAMTS19 | 0.0155415 | -2.02252 |
| HIST1H4A | 0.0129631 | -1.93723 | DDB2 | 0.0142488 | -2.02277 |
| TRERF1 | 0.00299111 | -1.93972 | SLC7A4 | 0.0360427 | -2.02478 |
| ELTD1 | 0.00306059 | -1.94304 | C15orf48 | 0.00237774 | -2.02618 |
| TNFRSF11B | 0.047084 | -1.94468 | FAM117B | 0.02049 | -2.02684 |
| ST3GAL5 | 0.00717268 | -1.94564 | GPR179 | 0.0471732 | -2.03225 |
| TNFRSF10C | 0.000208728 | -1.94574 | CDCP1 | 0.0207959 | -2.03703 |
| C12orf70 | 0.0318504 | -1.94742 | TP53INP1 | 0.000192707 | -2.03705 |
| GJC2 | 0.0089249 | -1.94782 | MAPK10 | 0.00878403 | -2.03901 |
| NDRG4 | 0.00359485 | -1.94904 | OR52K2 | 0.0151664 | -2.04112 |
| HIST1H4H | 0.00813219 | -1.95217 | FKBPL | 0.00414387 | -2.0438 |
| MTMR9L | 0.000226335 | -1.95239 | NINJ1 | 0.000152413 | -2.04386 |
| PTGFR | 0.0404748 | -1.95551 | GPRC5C | 0.00673569 | -2.04442 |
| CLDN11 | 0.0101785 | -1.95669 | RTTN | 0.0411547 | -2.04525 |
| SC5DL | 0.00981905 | -1.9592 | DQ895628 | 0.0284148 | -2.04585 |
| PDZRN3 | 0.000667329 | -1.96065 | DCUN1D2 | 0.00446433 | -2.04922 |
| GNAZ | 0.00344932 | -1.96067 | C3orf71 | 0.0375797 | -2.05171 |
| SGIP1 | 0.00290057 | -1.96324 | HTRA3 | 0.0381453 | -2.0537 |
| GPR153 | 0.0493253 | -1.96692 | LOC440082 | 0.0219406 | -2.05736 |
| TNXB | 0.00405074 | -1.96984 | CRTC1 | 0.00123513 | -2.06474 |
| HMOX1 | 0.000217658 | -1.9699 | TXLNB | 0.0137409 | -2.06565 |
| CACNA1G | 0.0163947 | -1.9703 | VWF | 0.00134621 | -2.06973 |
| PHEX | 0.0135048 | -1.97352 | ZNF821 | 0.0257483 | -2.07192 |
| TMEM140 | 0.00370241 | -1.97784 | RASL12 | 0.0151539 | -2.07496 |
| AKR1C4 | 0.0431062 | -1.98033 | DBNDD1 | 0.0280648 | -2.07513 |
| LOC401357 | 0.0190159 | -1.98697 | PLD1 | 0.0392563 | -2.0833 |
| POSTN | 0.0243548 | -1.98888 | CHRDL1 | 0.0128169 | -2.08377 |
| DYTN | 0.0050043 | -1.98893 | HIST1H4E | 0.00600529 | -2.08387 |
| MRPS6 | 0.00154995 | -1.98902 | TNFAIP2 | 0.00863218 | -2.09067 |
| ENC1 | 0.0245506 | -1.98922 | ZBTB46 | 0.00472291 | -2.09757 |
| PIK3C2B | 0.0242041 | -1.98927 | CCDC30 | 0.0259982 | -2.09826 |
| C5orf4 | 0.019397 | -1.98959 | LAMB3 | 0.000276104 | -2.10045 |
| FLJ41603 | 0.0211472 | -1.99124 | IER3 | 0.00122035 | -2.10279 |
| AK123797 | 0.0200231 | -1.99217 | FLJ44253 | 0.0364392 | -2.1055 |
| LOC100128055 | 0.0245863 | -1.99254 | PER2 | 0.00463593 | -2.1093 |
| MBP | 0.00896874 | -1.99458 | TAC3 | 0.0150556 | -2.11262 |
| AF072164 | 0.040833 | -1.99467 | AIF1L | 0.0367536 | -2.11437 |
| LOC100130996 | 0.00565763 | -1.99496 | GGTLC1 | 0.00294074 | -2.11674 |
| UACA | 0.00209645 | -1.99606 | ITGA7 | 0.000358097 | -2.11706 |
| MSR1 | 0.0123097 | -1.99945 | USP6NL | 0.0209651 | -2.1199 |
| TRO | 0.000815526 | -2.00129 | VASH2 | 0.0274618 | -2.12432 |
| SH2B2 | 0.00188046 | -2.12618 | SLC5A3 | 0.00609932 | -2.26351 |
| PAQR5 | 0.00279041 | -2.12679 | DCHS1 | 0.00523407 | -2.26518 |
| IRAK2 | 0.00288731 | -2.13016 | CSGALNACT1 | 0.0215879 | -2.26544 |
| DRAM1 | 6.05E-05 | -2.13031 | SMAD1 | 0.00177219 | -2.26762 |
| MGC42105 | 0.0481199 | -2.13361 | UNC5B | 0.00149188 | -2.26793 |
| KRT19 | 0.0252756 | -2.13385 | NFATC4 | 0.00588824 | -2.27027 |
| ZNF606 | 0.0201636 | -2.13848 | LOC285141 | 0.0307332 | -2.27033 |
| SPTLC3 | 0.00133782 | -2.13921 | TNC | 0.000485016 | -2.27137 |
| DENND5B | 0.00502217 | -2.14032 | CRABP2 | 0.000284095 | -2.27441 |
| ENST00000390301 | 0.00922738 | -2.1436 | ITGA11 | 0.0127115 | -2.27469 |
| PRRX1 | 0.00407039 | -2.14525 | TNFRSF25 | 0.00860519 | -2.2751 |
| MATN2 | 0.0160175 | -2.14538 | AMIGO2 | 0.0188621 | -2.27692 |
| GADD45G | 0.00577251 | -2.14843 | PLA2G4A | 0.0178399 | -2.28179 |
| HOXA3 | 0.0293659 | -2.14896 | RELB | 0.000732311 | -2.28955 |
| HTR2B | 0.0059829 | -2.15029 | PCNXL2 | 0.011275 | -2.29363 |
| QPRT | 0.000322493 | -2.15416 | ITPKB | 0.00376458 | -2.29408 |
| LOC728903 | 0.0325493 | -2.15499 | LOC728392 | 0.0123275 | -2.29463 |
| CCRL1 | 0.0458366 | -2.15526 | FAIM2 | 0.00390309 | -2.29614 |
| ABCB4 | 0.025316 | -2.16359 | OLFML1 | 0.0146794 | -2.2974 |
| SECTM1 | 0.0056696 | -2.164 | C14orf132 | 0.00485107 | -2.29851 |
| BC030764 | 0.00261796 | -2.16711 | PLEKHG1 | 0.0105358 | -2.30307 |
| TNNC2 | 0.00433159 | -2.17335 | PLEKHG4 | 0.00113659 | -2.30361 |
| KILLIN | 0.0206867 | -2.18127 | THC2564554 | 0.043092 | -2.30396 |
| ADAM8 | 0.00359133 | -2.19147 | TYMS | 0.018108 | -2.30487 |
| ENPP2 | 0.0484056 | -2.19226 | MAF | 0.00262797 | -2.30716 |
| CCDC48 | 0.00484607 | -2.19337 | FOLR3 | 0.00112763 | -2.30988 |
| RNF122 | 0.00145982 | -2.20561 | TCEA3 | 0.00213543 | -2.31001 |
| LOC390251 | 0.024576 | -2.20659 | COL4A5 | 0.0229976 | -2.31249 |
| MBD5 | 0.00217481 | -2.20768 | ARHGAP28 | 0.0363884 | -2.31275 |
| MAFB | 0.0283171 | -2.20814 | ZNF323 | 0.0437756 | -2.31434 |
| RGS5 | 0.0197477 | -2.21163 | HOXA10 | 0.0222964 | -2.32598 |
| LOC157562 | 0.0223075 | -2.22041 | PPARGC1A | 0.0211435 | -2.327 |
| SEMA3B | 0.000894984 | -2.22444 | ETV1 | 0.00189265 | -2.32789 |
| C17orf44 | 0.0333928 | -2.22605 | TMEM130 | 0.00144731 | -2.33212 |
| ZFP14 | 0.0192304 | -2.22655 | ZNF483 | 0.017416 | -2.335 |
| LOC645431 | 0.0303829 | -2.22744 | CIT | 0.00783073 | -2.33737 |
| ZNF521 | 0.0048713 | -2.22913 | ABCA11P | 0.0152178 | -2.33772 |
| MALAT1 | 0.0489195 | -2.23252 | COL14A1 | 0.00182548 | -2.33831 |
| THC2539584 | 0.049034 | -2.23648 | LPPR3 | 0.036024 | -2.33837 |
| CD36 | 0.0109877 | -2.23897 | HBD | 0.0243098 | -2.3389 |
| ABCA1 | 0.0207415 | -2.24225 | VAT1L | 0.0354067 | -2.34081 |
| SYTL2 | 0.00331061 | -2.24309 | PHGDH | 0.0107903 | -2.34366 |
| FLJ13197 | 0.0435998 | -2.24525 | SPTBN4 | 0.00138058 | -2.34695 |
| GBP2 | 0.00375895 | -2.24538 | FAM134B | 0.0222865 | -2.34911 |
| PLXDC1 | 0.00825863 | -2.25051 | C18orf56 | 0.00985652 | -2.35272 |
| ZNF165 | 0.0347887 | -2.25079 | CRYM | 0.00051315 | -2.35813 |
| ZNF221 | 0.0353176 | -2.2514 | THC2728054 | 0.0168281 | -2.36191 |
| LRDD | 0.00229135 | -2.25431 | LOC283454 | 0.00151061 | -2.36518 |
| GPR62 | 0.00734075 | -2.26238 | IL13RA2 | 0.0386779 | -2.3665 |
| C1orf228 | 0.00199302 | -2.37091 | Sep-12 | 0.017346 | -2.61068 |
| LOC203510 | 0.0105021 | -2.37188 | GDF10 | 0.0106163 | -2.61162 |
| RGS2 | 0.0216326 | -2.3742 | PHLDA1 | 0.00163238 | -2.62249 |
| CA14 | 0.0109646 | -2.37797 | FLJ41484 | 0.0225578 | -2.6502 |
| RAP2B | 0.0012435 | -2.37995 | KRTAP13-2 | 0.0343849 | -2.66001 |
| NHS | 0.00816653 | -2.38049 | LRRC25 | 0.0404329 | -2.68019 |
| HSPA4L | 0.00502894 | -2.38086 | FLJ22536 | 0.0335532 | -2.68094 |
| LUM | 0.00459266 | -2.38331 | FAM171B | 0.00253607 | -2.68106 |
| DACT3 | 0.00361146 | -2.38466 | KLF4 | 0.0263349 | -2.686 |
| NEDD4L | 0.00340027 | -2.39217 | OR10G9 | 0.0347738 | -2.70534 |
| RCOR2 | 0.00273849 | -2.39429 | CDKN2B | 0.00010202 | -2.70931 |
| TMEM158 | 0.0182022 | -2.40322 | SLC7A14 | 0.000666958 | -2.72656 |
| ADAMTS14 | 0.0347588 | -2.40669 | SALL2 | 0.00557715 | -2.72715 |
| RSPO3 | 0.0135325 | -2.4155 | TARSL2 | 0.000528049 | -2.72922 |
| FDXR | 0.000493899 | -2.41657 | ENST00000402420 | 0.0388179 | -2.73066 |
| SNCAIP | 0.0003216 | -2.41885 | TMCC2 | 0.0362454 | -2.73384 |
| GRAMD1A | 0.00304616 | -2.42083 | TNFSF13B | 0.0457193 | -2.75552 |
| FAM102A | 6.19E-05 | -2.42137 | BAALC | 0.00914276 | -2.76266 |
| FLRT2 | 0.000293604 | -2.42699 | GDF15 | 0.000129115 | -2.77627 |
| PRRT2 | 0.0171098 | -2.42928 | VWCE | 0.0156536 | -2.79722 |
| BBC3 | 0.00116986 | -2.43318 | C6orf138 | 0.00222058 | -2.79844 |
| PLA2G4C | 0.000799513 | -2.43367 | RAB7B | 0.000687243 | -2.80818 |
| OXER1 | 0.00136629 | -2.44069 | RDH5 | 0.00231506 | -2.81584 |
| KCNMA1 | 0.0031953 | -2.44479 | IGDCC4 | 0.00148491 | -2.81815 |
| SH3BP5 | 0.0181129 | -2.45401 | EPHB3 | 0.000703597 | -2.82279 |
| CHRDL2 | 0.0260872 | -2.4558 | RCAN2 | 0.0011036 | -2.82403 |
| CGNL1 | 0.0134285 | -2.46573 | CLGN | 0.000254954 | -2.8298 |
| FST | 0.00322235 | -2.46728 | CNTN3 | 0.026407 | -2.83565 |
| PLAU | 0.00127162 | -2.47345 | OR2Y1 | 0.022669 | -2.83964 |
| C20orf195 | 0.00312693 | -2.47866 | DIP2A | 0.0212757 | -2.85123 |
| GFRA1 | 0.00105592 | -2.48121 | LOC100130111 | 0.00387314 | -2.88354 |
| CA12 | 0.00571127 | -2.48146 | IGSF10 | 0.00879671 | -2.88593 |
| ZNF846 | 0.023978 | -2.48209 | FIGF | 0.0321458 | -2.89233 |
| SAMD12 | 0.00558809 | -2.48494 | P2RX7 | 0.000988212 | -2.90941 |
| SMO | 0.00162981 | -2.48883 | HSD17B2 | 0.0113764 | -2.90988 |
| ACP5 | 0.000682876 | -2.48975 | PKP3 | 0.00291831 | -2.94756 |
| DDIT4L | 0.0392359 | -2.50747 | KCTD12 | 0.0190956 | -2.96429 |
| RASSF2 | 0.0125668 | -2.51323 | LOC646936 | 0.00213994 | -2.9659 |
| ARVCF | 0.000616711 | -2.52069 | LDB2 | 0.000510225 | -3.00525 |
| GDF1 | 0.013016 | -2.5208 | AQP3 | 0.0357826 | -3.00815 |
| ADCY1 | 0.0106139 | -2.54299 | RFTN2 | 0.00133565 | -3.02176 |
| IL17RD | 0.01906 | -2.54413 | MIAT | 0.00476707 | -3.02631 |
| AF359419 | 0.0315234 | -2.55552 | LRRC15 | 0.0244118 | -3.04724 |
| LOC100132815 | 8.29E-05 | -2.55603 | MX1 | 0.00333702 | -3.05139 |
| LOC729603 | 0.0119687 | -2.55654 | KIAA2026 | 0.0307381 | -3.05586 |
| RASGRF2 | 0.0228091 | -2.56552 | LASS1 | 0.00484164 | -3.05796 |
| FLJ37644 | 0.0139146 | -2.5689 | CTTNBP2 | 0.0321254 | -3.06626 |
| ADAMTSL1 | 0.0397345 | -2.57095 | ARSI | 0.0070051 | -3.07722 |
| GYG2 | 0.0377113 | -2.5869 | RAB33A | 0.000675221 | -3.0787 |
| PCDHB5 | 0.00579755 | -3.08415 | FGF13 | 0.00643471 | -3.76609 |
| ENST00000423322 | 0.0136764 | -3.08419 | E2F7 | 0.000767994 | -3.78745 |
| PRKCG | 0.00417623 | -3.09221 | SOX4 | 0.0153381 | -3.82857 |
| C17orf76 | 0.000836986 | -3.11032 | MEX3A | 0.00161493 | -3.8398 |
| TMEM35 | 0.00311096 | -3.13942 | TNFAIP6 | 0.00422564 | -3.87415 |
| ANGPTL2 | 5.47E-05 | -3.13951 | TRIM45 | 0.000116789 | -3.92432 |
| DCLK1 | 0.0241395 | -3.18545 | SYT7 | 0.000705314 | -3.94339 |
| MEX3B | 0.0441479 | -3.19048 | GLI1 | 0.00714094 | -3.95142 |
| TMEM217 | 0.000680899 | -3.20839 | CXCL12 | 0.00359088 | -3.96001 |
| FSTL5 | 0.0432873 | -3.2283 | MXRA5 | 5.14E-05 | -3.96914 |
| GPER | 0.00214358 | -3.23689 | GPR68 | 0.00266368 | -4.32065 |
| MYBPH | 0.0398398 | -3.23747 | PDE4B | 0.0199266 | -4.39107 |
| EVI2A | 0.00133385 | -3.25217 | CCL11 | 0.00543929 | -4.42498 |
| DACH1 | 0.0308535 | -3.25745 | RRAD | 0.00650411 | -4.68968 |
| AMOT | 0.00728667 | -3.26956 | LRRN3 | 0.0207673 | -4.70784 |
| IFIT1 | 0.000709866 | -3.31245 | RAB26 | 0.00377299 | -4.96857 |
| LRRC17 | 0.0307473 | -3.32752 | FAM46C | 0.00140449 | -5.03319 |
| LOC375196 | 0.0328343 | -3.35661 | CD274 | 0.0269718 | -5.10222 |
| OR1K1 | 0.00541602 | -3.35979 | BDKRB1 | 0.000679002 | -5.22339 |
| ENST00000331733 | 0.0103568 | -3.36124 | GPR56 | 0.00348262 | -5.43723 |
| LOC283070 | 0.0138072 | -3.36408 | LOC649941 | 0.0373539 | -5.4505 |
| SLC6A9 | 0.0001186 | -3.4027 | CNIH3 | 0.000112112 | -5.56423 |
| BDKRB2 | 0.000469808 | -3.43774 | NR0B1 | 0.0043313 | -5.78435 |
| LOC100132167 | 0.0226973 | -3.44223 | MMP10 | 0.0199715 | -5.85228 |
| ISG20 | 0.00891136 | -3.47533 | NOV | 2.59E-05 | -6.38307 |
| SEMA3A | 0.00853538 | -3.48542 | SIPA1L2 | 0.00144401 | -6.38375 |
| NFE2 | 0.000617891 | -3.5063 | IL4I1 | 0.00200558 | -6.40553 |
| CCL7 | 0.0349931 | -3.53607 | CHST8 | 0.0353442 | -6.63884 |
| HSPA12B | 0.0131438 | -3.59906 | OR6N1 | 0.0141912 | -7.02028 |
| CH25H | 0.00893032 | -3.64039 | G0S2 | 0.00103256 | -7.60972 |
| COL13A1 | 0.00136208 | -3.72113 | MMP1 | 0.00057377 | -9.69951 |
| ALDH1A3 | 0.0145858 | -3.76022 | HLA-DRB3 | 0.0408609 | -12.2717 |

**Supplemental Table 9: Common mRNA expression changes in ASM cells from patients with non-severe and severe asthma following treatment with dexamethasone (10^-7^ M), before stimulation with FCS (2.5 %)**

|  |  | Non-Severe Asthma | Severe Asthma |
| --- | --- | --- | --- |
| Gene Symbol | **Gene Name** | **Microarray (FC)** | **Microarray (FC)** |
| ASNSD1 | Asparagine synthetase domain containing 1 | -2.1 (< .01) | 11.0 (< .01) |
| BEND6 | BEN domain containing 6 | -1.7 (< .01) | -1.8 (< .01) |
| C16orf74 | Chromosome 16 open reading frame 74 | -1.8 (< .01) | -1.6 (< .01) |
| C6orf108 | 2'-Deoxynucleoside 5'-Phosphate N-Hydrolase 1 | -1.8 (< .01) | 17.9 (< .01) |
| C9orf140 | Suppressor APC Domain Containing 2 | -1.8 (< .01) | -1.5 (< .01) |
| CAPNS2 | Calpain, small subunit 2 | -1.7 (< .01) | 6.7 (< .01) |
| CD36 | CD36 Molecule (Thrombospondin Receptor) | -2.3 (< .01) | -2.2 (< .01) |
| DCTN3 | Dynactin 3 (p22) | -2.0 (< .01) | 18.0 (< .01) |
| EFR3B | EFR3 homolog B | 1.8 (< .01) | -1.8 (< .01) |
| GYG2 | Glycogenin 2 | -2.1 (< .01) | -2.6 (< .01) |
| OLFML1 | Olfactomedin-like 1 | 2.0 (< .01) | -2.3 (< .01) |
| SDSL | Serine dehydratase-like | -1.5 (< .01) | 8.7 (< .01) |
| SHC2 | Src homology-2 domain containing transf protein 2 | 5.1 (< .01) | -1.8 (< .01) |
| SNRNP27 | Small nuclear ribonucleoprotein 27kDa | -3.5 (< .01) | 11.3 (< .01) |
| TRIP12 | Thyroid hormone receptor interactor 12 | -1.7 (< .01) | 22.9 (< .01) |
| VLDLR | Very low density lipoprotein receptor | -1.9 (< .01) | -1.8 (< .01) |
| ZBTB16 | Zinc finger and BTB domain containing 16 | -1.7 (< .01) | 16.2 (< .01) |

**Supplemental Table 10: Baseline lncRNA expression changes in ASM cells from patients with non-severe asthma compared to healthy patients**

|  |  | |  | | Flanking Loci | | |
| --- | --- | --- | --- | --- | --- | --- | --- |
| Class of lncRNA | **Ensemble gene ID** | **Transcript** | | **FC** | | **5 Prime** | **3 Prime** |
| lincRNA | ENST00000514823 | RP11-93L9.1 | | 2.1 (< .05) | | SPATA5 | SPRY1 |
| lincRNA | ENST00000434601 | LINC00422 | | 1.9 (< .05) | | FGF9 | BASP1P1 |
| lincRNA | ENST00000450063 | AC006159.3 | | 1.9 (< .05) | | MET | CAPZA2 |
| lincRNA | ENST00000438897 | AC068491.2 | | 1.8 (< .05) | | BCC2L11 | LOC541471 |
| lincRNA | ENST00000454968 | LINC00963 | | 1.8 (< .05) | | NCS1 | ASS1 |
| lincRNA | ENST00000415714 | RP1-60O19.1 | | 1.8 (< .05) | | ORSL1 | NR_033557 |
| lincRNA | ENST00000437696 | RP11-359G22.2 | | 1.8 (< .05) | | LINC00261 | SSTR4 |
| lincRNA | ENST00000560760 | RP11-38G5.2 | | 1.7 (< .05) | | MEX3B | EFTUD1 |
| lincRNA | ENST00000413945 | LINC00472 | | 1.6 (< .05) | | B3GAT2 | RIM51 |
| lincRNA | ENST00000443965 | GS1-600G8.5 | | 1.6 (< .05) | | NR_045260 | EGFL6 |
| lincRNA | ENST00000456532 | RP5-1158E12.3 | | 1.6 (< .05) | | CXORF36 | ZNF673 |
| lincRNA | ENST00000412685 | HCG18 | | 1.6 (< .05) | | TRIM15 | HLA-L |
| Processed Transcript | ENST00000447323 | MSN | | 1.5 (< .05) | | MSN | MSN |
| lincRNA | ENST00000503532 | RP11-341G5.1 | | 1.5 (< .05) | | LOC152742 | LOC441009 |
| lincRNA | ENST00000418358 | AC011747.3 | | 1.5 (< .05) | | LINC00299 | IDZ |
| lincRNA | ENST00000524165 | *PVT1* | | -1.5 (< .05) | | MYC | LOC728724 |
| lincRNA | ENST00000446423 | FKBP1A-SDCBP2 | | -1.6 (< .05) | | SDCBP2 | NSFLIC |
| Antisence | ENST00000501164 | SDCBP2-AS1 | | -1.6 (< .05) | | TP531NP1 | C8ORF38 |
| lincRNA | ENST00000443576 | RP11-141M1 | | -1.8 (< .05) | | RFC3 | NBEA |
| lincRNA | ENST00000513480 | CTD-2127H9.1 | | -1.8 (< .05) | | LIFR | OSMR |
| lincRNA | ENST00000414790 | H19 | | -3.0 (< .05) | | DBX1 | HTATIP2 |

**Supplemental Table 11: Baseline lncRNA expression changes in ASM cells from patients with severe asthma compared to healthy patients**

|  |  | |  | | Flanking Loci | | |
| --- | --- | --- | --- | --- | --- | --- | --- |
| Class of lncRNA | **Ensemble gene ID** | **Name** | | **FC** | | **5 Prime** | **3 Prime** |
| lincRNA | ENSG00000230590 | FTX | | 1.8 (< .05) | | ZCCHC13 | SLC16A2 |
| Antisence | ENST00000507244 | STX18-AS1 | | 1.8 (< .05) | | D4S234E | NR_037888 |
| lincRNA | ENST00000418006 | LINC00940 | | 1.7 (< .05) | | CACNA1C | LOC28344O |
| lincRNA | ENSG00000233237 | LINC00472 | | 1.7 (< .05) | | B3GAT2 | RIM51 |
| Antisence | ENST00000433079 | MKLN1-AS1 | | 1.6 (< .05) | | MKLN1 | PODXL |
| lincRNA | ENSG00000237879 | LINC00398 | | 1.6 (< .05) | | EEFIDP3 | FRY |
| lincRNA | ENST00000521586 | RP11-382A18.2 | | 1.6 (< .05) | | PCAT1 | POU5F1B |
| lincRNA | ENST00000440496 | LINC00630 | | 1.5 (< .05) | | NR_038988 | BEX1 |
| lincRNA | ENST00000456532 | RP5-1158E12.3 | | 1.5 (< .05) | | CXORF36 | ZNF673 |
| lincRNA | ENST00000520431 | RP11-527N22.2 | | 1.5 (< .05) | | KCNU1 | ZNF703 |
| lincRNA | ENSG00000249859 | *PVT1* | | 1.5 (< .05) | | MYC | LOC728724 |
| lincRNA | ENST00000448786 | AC007879.2 | | 1.5 (< .05) | | KLF7 | CRABI |
| lincRNA | ENST00000433747 | RP11-120D5.1 | | 1.5 (< .05) | | MID1 | ARHGAP6 |
| lincRNA | ENSG00000088832 | FKBP1A-SDCBP2 | | -1.5 (< .05) | | SDCBP2 | NSFLIC |
| lincRNA | ENST00000420774 | AC004540.5 | | -1.5 (< .05) | | SNX10 | LOC441204 |
| lincRNA | ENST00000424283 | RP1-261G23.5 | | -1.6 (< .05) | | GTPBP2 | MAD2L1BP |

**Supplemental Table 12: lncRNA expression changes in ASM cells from patients with non-severe asthma after exposure with FCS (2.5 %)**

|  |  | |  | | Flanking Loci | |
| --- | --- | --- | --- | --- | --- | --- |
| Class of lncRNA | **Ensemble gene ID** | **Name** | **FC** | **5 Prime** | | **3 Prime** |
| lincRNA | ENST00000552334 | RP11-701H24.2 | 5.6 (< .05) | GABRA5 | | OCA2 |
| Antisence | ENST00000439601 | AC131097.3 | 4.1 (< .05) | CXXC11 | | AC093642 |
| lincRNA | ENST00000415611 | AC005682.5 | 3.5 (< .05) | *IL6* | | KLHL7 |
| lincRNA | ENST00000529893 | RP1-80B9 | 3.2 (< .05) | FOXC1 | | C6orf195 |
| Sense overlapping | ENST00000304425 | MIR31 host gene | 3.1 (< .05) | IFNA8 | | C9orf53 |
| lincRNA | ENST00000443576 | RP11-141M1 | 2.7 (< .05) | STARD13 | | RFC3 |
| lincRNA | ENST00000508406 | RP11-8L2.1 | 2.5 (< .05) | AGPAT9 | | NKX6-1 |
| Processed transcript | ENST00000447430 | AC016831.7 | 2.2 (< .05) | KLF14 | | MKLN1 |
| lincRNA | ENST00000553465 | MEG8-001 | 2.0 (< .05) | MIR136 | | MIR1197 |
| lincRNA | ENST00000435643 | AC007879.1 | 1.8 (< .05) | MIR2355 | | MIR1302 |
| lincRNA | ENST00000433843 | SNHG5 | 1.8 (< .05) | SYNCRIP | | RN7SL643P |
| lincRNA | ENST00000561123 | RP11-307C19.2 | 1.6 (< .05) | HMG20A | | LINGO1 |
| lincRNA | ENST00000435702 | AP001046 | 1.6 (< .05) | CRYAA | | SIK1 |
| lincRNA | ENST00000505254 | MIR143 host gene | 1.6 (< .05) | GRPEL2 | | CSNK1A1 |
| Sense overlapping | ENST00000314957 | CTD-2201E18.3 | 1.5 (< .05) | CCDC152 | | NIM1 |
| lincRNA | ENST00000417947 | AC096574.5 | -1.5 (< .05) | MLPH | | RBM44 |
| lincRNA | ENSG00000249859 | *PVT1* | -1.6 (< .05) | MYC | | LOC728724 |
| lincRNA | ENST00000414120 | LINC00887 | -1.7 (< .05) | ATP13A4 | | CPN2 |
| Antisence | ENST00000511571 | RP11-453E17 | -1.7 (< .05) | STAP1 | | TMPRSS11BNL |
| lincRNA | ENST00000423943 | RP11-48O20.4 | -1.8 (< .05) | DUSP23 | | PIGM |
| lincRNA | ENST00000418539 | BCYRN1 | -1.8 (< .05) | CALM2 | | AC138655 |
| Antisence | ENST00000422059 | RP5-1120P11.1 | -1.8 (< .05) | VEGFA | | MRPL14 |
| lincRNA | ENST00000464767 | LINC00341 | -1.9 (< .05) | DICER1 | | TCL1A |
| lincRNA | ENST00000373171 | LINC00951 | -1.9 (< .05) | MOCS1 | | UNC5CL |
| lincRNA | ENST00000518765 | RP11-527N22.1 | -2.0 (< .05) | KCNU1 | | ERLIN2 |
| lincRNA | ENST00000473636 | LINC00882 | -2.0 (< .05) | CBLB | | BBX |
| lincRNA | ENST00000457340 | RP11-503C24.1 | -2.2 (< .05) | AL009178.1 | | DACT2 |
| lincRNA | ENST00000514844 | RP11-46C20.1 | -2.3 (< .05) | CDH9 | | CDH6 |
| lincRNA | ENST00000451230 | AC108463.1 | -2.5 (< .05) | BCL2L11 | | MIR4435 |
| lincRNA | ENST00000455957 | HCG17 | -2.8 (< .05) | TRIM15 | | TRIM39 |
| lincRNA | ENST00000506086 | RP11-229C3.2 | -3.2 (< .05) | GCNT4 | | ANKRD31 |

**Supplemental Table 13: lncRNAs in non-severe ASM changed in expression following treatment with dexamethasone (10^-7^ M), before stimulation with FCS (2.5 %)**

|  |  | |  | | Flanking Loci | |
| --- | --- | --- | --- | --- | --- | --- |
| Class of lncRNA | **Ensemble gene ID** | **Name** | **FC** | **5 Prime** | | **3 Prime** |
| lincRNA | ENSG00000236605 | ACO23115.4 | 3.9 (< .05) | MEIS1 | | ETAA1 |
| Antisence | ENSG00000238273 | ACO12360.6 | 3.6 (< .05) | MRPS9 | | NCK2 |
| lincRNA | ENSG00000258123 | RP11-314D7.2 | 3.5 (< .05) | TRHDE | | ATXN7L3B |
| Antisence | ENSG00000270953 | RP11-2E11.9 | 3.4 (< .05) | MEST | | MIR29 |
| lincRNA | ENSG00000259331 | RP11-57P19.1 | 3.2 (< .05) | MCTP2 | | NR2F2 |
| lincRNA | ENSG00000273615 | RP11-1136G4.2 | 3.1 (< .05) | IRX3 | | IRX5 |
| lincRNA | ENSG00000279440 | CTA-992D9.11 | 3.0 (< .05) | CRYBA4 | | MN1 |
| lincRNA | ENSG00000272798 | CTA-390C10.9 | 3.0 (< .05) | LRP5L | | ADREK2 |
| lincRNA | ENSG00000254275 | lincRNA00824 | 3.0 (< .05) | TMEM75 | | GSDMC |
| lincRNA | ENSG00000231082 | RP11-514F8.2 | 3.0 (< .05) | SH2D4B | | NRG3 |
| lincRNA | ENSG00000256748 | RP11-234B24.2 | 2.9 (< .05) | RAD51AP1 | | GALNT8 |
| lincRNA | ENSG00000204603 | lincRNA01257 | 2.8 (< .05) | GPR133 | | SFSWAP |
| lincRNA | ENSG00000229323 | DLEU1-AS1 | 2.7 (< .05) | DLEU1 | | DLEU7 |
| lincRNA | ENSG00000237756 | RP11-77M5.1 | 2.7 (< .05) | NUF2 | | PBX1 |
| lincRNA | ENSG00000260604 | RP1-140K8.5 | 2.6 (< .05) | FAM50B | | PRPF4B |
| lincRNA | ENSG00000266869 | RP6-114E22.1 | 2.6 (< .05) | SIPA1L1 | | RGS6 |
| lincRNA | ENSG00000271860 | RP11-436D23.1 | 2.5 (< .05) | MMS22L | | POU3F2 |
| lincRNA | ENSG00000245149 | RNF139-AS1 | 2.5 (< .05) | TRMT12 | | RNF139 |
| lincRNA | ENSG00000244342 | lincRNA00698 | 2.4 (< .05) | CADPS | | SYNPR |
| lincRNA | ENSG00000267055 | RP11-486P11.1 | 2.4 (< .05) | TMEM196 | | MACC1 |
| lincRNA | ENSG00000230156 | lincRNA00443 | 2.4 (< .05) | ARGLU1 | | FAM155A |
| lincRNA | ENSG00000231671 | lincRNA01307 | 2.4 (< .05) | SIPR1 | | OLFM3 |
| lincRNA | ENSG00000236983 | lincRNA00614 | 2.4 (< .05) | ABI1 | | YME1L1 |
| lincRNA | ENSG00000233365 | RP4-655C5.4 | 2.3 (< .05) | T | | PRR18 |
| lincRNA | ENSG00000226673 | lincRNA01108 | 2.2 (< .05) | CD83 | | JARID2 |
| lincRNA | ENSG00000278630 | RP11-78L16.1 | 2.2 (< .05) | OLFM4 | | PRR20A |
| Antisence | ENSG00000247381 | PDX1-AS1 | 2.2 (< .05) | GSX1 | | PDX1 |
| lincRNA | ENSG00000235152 | RP5-865N13.2 | 2.2 (< .05) | DISC1 | | SIPA1L2 |
| lincRNA | ENSG00000241475 | RP4-781K5.5 | 2.1 (< .05) | IRF2BP2 | | TOMM20 |
| lincRNA | ENSG00000270604 | HCG17 | 2.1 (< .05) | TRIM26 | | HLA-L |
| Sense intronic | ENSG00000276672 | RP11-142E9.1 | 2.0 (< .05) | RFC3 | | NBEA |
| lincRNA | ENSG00000272168 | CASC15 | 2.0 (< .05) | SOX4 | | PRL |
| lincRNA | ENSG00000230587 | AC093609.1 | 2.0 (< .05) | HAAO | | ZFP36L2 |
| lincRNA | ENSG00000233723 | lincRNA01122 | 1.9 (< .05) | FANCL | | BCL11A |
| lincRNA | ENSG00000228739 | RP11-21817.2 | 1.9 (< .05) | MLANA | | IL33 |
| lincRNA | ENSG00000228798 | AP000473.5 | 1.9 (< .05) | MIR125B2 | | CXADR |
| lincRNA | ENSG00000219445 | RP11-3B12.3 | 1.9 (< .05) | POT1 | | GRM8 |
| lincRNA | ENSG00000260986 | RP11-854K16.3 | 1.9 (< .05) | POTEB2 | | POTEB3 |
| lincRNA | ENSG00000246084 | CTD-2506J14.1 | 1.9 (< .05) | VRK1 | | C14ORF177 |
| lincRNA | ENSG00000223863 | AC008074.4 | 1.8 (< .05) | LGALSL | | AFTPH |
| lincRNA | ENSG00000226375 | RP3-395P12.2 | 1.8 (< .05) | TNFSF18 | | TNFSF4 |
| lincRNA | ENSG00000224717 | RP11-576D8.4 | 1.8 (< .05) | LEMD1 | | CDK18 |
| lincRNA | ENSG00000214870 | AC004540.5 | 1.7 (< .05) | SNX10 | | KIAA0087 |
| lincRNA | ENSG00000232413 | RP11-343J18.2 | 1.7 (< .05) | PBX3 | | MVB12B |
| lincRNA | ENSG00000267586 | lincRNA00970 | 1.7 (< .05) | PIK3C3 | | RIT2 |
| lincRNA | ENSG00000272144 | CTD-2035E11.5 | 1.7 (< .05) | ANXA2R | | ZNF131 |
| lincRNA | ENSG00000248300 | RP11-74M11.2 | 1.7 (< .05) | HS3ST1 | | RAB28 |
| lincRNA | ENSG00000257086 | RP11-783K16.13 | 1.7 (< .05) | FERMT3 | | BAD |
| lincRNA | ENSG00000239482 | RP11-90K6.1 | 1.6 (< .05) | SLC9C1 | | BTLA |
| lincRNA | ENSG00000259198 | RP11-133K1.6 | 1.6 (< .05) | C15ORF52 | | DISP2 |
| lincRNA | ENSG00000230812 | lincRNA01358 | 1.5 (< .05) | JUN | | FGGY |
| Antisence | ENSG00000258982 | RP11-63812.4 | 1.5 (< .05) | DEGS2 | | YY1 |
| lincRNA | ENSG00000248371 | CTC-347C20.2 | 1.5 (< .05) | ZNF366 | | TNPO1 |
| lincRNA | ENSG00000224228 | RP1-15D23.2 | 1.5 (< .05) | FASLG | | TNFSF18 |
| lincRNA | ENSG00000263622 | RP11-389J22.3 | 1.5 (< .05) | CDH7 | | CDH19 |
| lincRNA | ENSG00000271788 | CTD-2201E18.5 | 1.4 (< .05) | CCDC152 | | ANXA2R |
| Antisence | ENSG00000224743 | TEX26-AS1 | 1.4 (< .05) | ALOX5AP | | MEDAG |
| lincRNA | ENSG00000255839 | RP11-338K17.8 | 1.3 (< .05) | TCTN2 | | ATP6VDA2 |
| lincRNA | ENSG00000228636 | RP5-1051H14.2 | 1.3 (< .05) | GATA3 | | CELF2 |
| lincRNA | ENSG00000259725 | CTD-3032H12.1 | 1.2 (< .05) | IRX3 | | IRX5 |
| lincRNA | ENSG00000248605 | CTD-2306M5.1 | -1.1 (< .05) | CDH10 | | CDH9 |
| lincRNA | ENSG00000268184 | RP11-420K14.8 | -1.3 (< .05) | ZNF100 | | ZNF43 |
| lincRNA | ENSG00000273100 | RP11-302L19.3 | -1.6 (< .05) | ERMARD | | DLL1 |
| lincRNA | ENSG00000249631 | RP11-281P23.2 | -1.6 (< .05) | HS3ST1 | | RAB28 |
| lincRNA | ENSG00000259504 | RP11-352D13.5 | -1.7 (< .05) | PAQR5 | | KIF23 |
| lincRNA | ENSG00000225269 | lincRNA00705 | -1.8 (< .05) | KLF6 | | AKR1E2 |
| lincRNA | ENSG00000235154 | CTA-280A3_B.2 | -1.8 (< .05) | FP325331.1 | | FAM19A5 |
| lincRNA | ENSG00000249021 | CTC-505O3.3 | -1.9 (< .05) | TICAM2 | | CDO1 |
| lincRNA | ENSG00000248515 | RP11-608O21.1 | -1.9 (< .05) | LCORL | | SLIT2 |
| lincRNA | ENSG00000236204 | lincRNAO1376 | -1.9 (< .05) | NT5C1B | | OSR1 |
| lincRNA | ENSG00000248588 | CTC-458G6.4 | -1.9 (< .05) | ARRDC3 | | NR2F1 |
| lincRNA | ENSG00000254275 | lincRNA00824 | -2.1 (< .05) | TMEM75 | | GSDMC |
| lincRNA | ENSG00000257859 | CASC18 | -2.4 (< .05) | C12ORF75 | | NUAK1 |
| lincRNA | ENSG00000251580 | RP11-539L10.3 | -2.4 (< .05) | MAN2B2 | | MRFAP1 |
| lincRNA | ENSG00000249740 | OSMR-AS1 | -2.4 (< .05) | LIFR | | OSMR |
| Antisence | ENSG00000233340 | RP11-25C19.3 | -2.7 (< .05) | VTI1A | | TCFL2 |
| lincRNA | ENSG00000239628 | RP11-543D10.2 | -2.7 (< .05) | SKIL | | CLDN11 |
| Antisence | ENSG00000224691 | GS1-174L6.4 | -3.4 (< .05) | HMCN1 | | PRG4 |
| lincRNA | ENSG00000270020 | RP11-463O9.9 | -3.8 (< .05) | IRF8 | | FOXF1 |

**Supplemental Table 14: lncRNA expression changes in ASM cells from patients with severe asthma after exposure with FCS (2.5 %)**

|  |  | |  | | Flanking Loci | |
| --- | --- | --- | --- | --- | --- | --- |
| Class of lncRNA | **Ensemble gene ID** | **Name** | **FC** | **5 Prime** | | **3 Prime** |
| lincRNA | ENST00000444958 | DANCR | 3.4 (< .05) | USP46 | | MIR4449 |
| Processed transcript | ENST00000443587 | DLEU2 | 3.2 (< .05) | MIR3613 | | DLEU7 |
| Processed transcript | ENST00000451141 | MIAT | 2.9 (< .05) | CRYBB1 | | MN1 |
| Antisence | ENST00000439601 | AC131097.3 | 2.5 (< .05) | CXXC11 | | CICP10 |
| lincRNA | ENST00000432823 | RP1-80N2.2 | 2.4 (< .05) | LY86 | | RREB1 |
| lincRNA | ENST00000438897 | AC068491.2 | 2.4 (< .05) | BCL2L11 | | MIR4435 |
| lincRNA | ENST00000478824 | CTD-2377D24.6 | 2.1 (< .05) | HOXB4 | | MIR3185 |
| lincRNA | ENST00000507058 | CTC-276P9.2 | 1.9 (< .05) | PITX1 | | H2AFY |
| lincRNA | ENST00000512519 | CTD-2127H9.1 | 1.9 (< .05) | LIFR | | OSMR |
| Processed transcript | ENST00000447430 | AC016831.7 | 1.8 (< .05) | KLF14 | | MKLN1 |
| lincRNA | ENST00000366424 | AC144450.2 | 1.7 (< .05) | TPO | | PXDN |
| Antisence | ENST00000435800 | RP11-31F15.1 | 1.7 (< .05) | SLC16A1 | | LRIG2 |
| Antisence | ENST00000460833 | ADAMTS9-AS2 | 1.7 (< .05) | ADAMTS9 | | MAGI1 |
| lincRNA | ENST00000446884 | RP1-30G7.2 | 1.7 (< .05) | MIR222 | | KRBOX4 |
| lincRNA | ENST00000560760 | RP11-38G5.2 | 1.5 (< .05) | KIAA1024 | | MTHFS |
| lincRNA | ENST00000416401 | RP11-77M5.1 | 1.5 (< .05) | RGS5 | | NUF2 |
| lincRNA | ENST00000433079 | AC058791.2 | -1.5 (< .05) | MIR29 | | MKLN1 |
| lincRNA | ENST00000552334 | RP11-701H24.2 | -1.5 (< .05) | SNURF | | UBE3A |
| Antisence | ENST00000519104 | RP3-399L15.3 | -1.5 (< .05) | HDAC2 | | FRK |
| lincRNA | ENST00000444265 | LINC00340 | -1.5 (< .05) | SOX4 | | PRL |
| lincRNA | ENST00000421322 | XIST | -1.6 (< .05) | CHIC1 | | ZCCHC13 |
| lincRNA | ENST00000464767 | LINC00341 | -1.6 (< .05) | CLMN | | SYNE3 |
| Antisence | ENST00000416630 | DCTN1-AS1 | -1.6 (< .05) | MTHFD2 | | MOGS |
| Sense intronic | ENST00000433544 | RP11-488P3.1 | -1.7 (< .05) | BCAR3 | | DNTTIP2 |
| Processed transcript | ENST00000419628 | AP001626.1 | -1.8 (< .05) | SLC37A1 | | PDE9A |
| lincRNA | ENST00000437261 | AC108066.1 | -1.9 (< .05) | MIR548 | | IKZF2 |
| Antisence | ENST00000434399 | AC005154.6 | -1.9 (< .05) | GGCT | | GARS |
| lincRNA | ENST00000514791 | RP11-434D9.2 | -2.0 (< .05) | CD180 | | PIK3R1 |
| lincRNA | ENST00000435643 | AC007879.1 | -2.2 (< .05) | KLF7 | | CREB1 |
| lincRNA | ENST00000433747 | RP11-120D5.1 | -2.2 (< .05) | MID1 | | ARHGAP6 |
| lincRNA | ENST00000418006 | LINC00940 | -2.3 (< .05) | LRTM2 | | DCP1B |
| lincRNA | ENST00000434601 | RP11-101P17.9 | -2.7 (< .05) | MRP63 | | ZDHHC20 |

**Supplemental Table 15: lncRNAs in severe ASM changed in expression following treatment with dexamethasone (10^-7^ M), before stimulation with FCS (2.5 %)**

|  |  |  |  | **Flanking Loci** | |
| --- | --- | --- | --- | --- | --- |
| **Class of lncRNA** | **Ensemble gene ID** | **Name** | **FC** | **5 Prime** | **3 Prime** |
| lincRNA | ENST00000444958 | DANCR | 6.3 (< .05) | USP46 | ERVMER34-1 |
| lincRNA | ENST00000473636 | LINC00882 | 3.4 (< .05) | CBLB | CCDC54 |
| Processed transcript | ENST00000443587 | DLEU2 | 3.1 (< .05) | TRIM13 | DLEU7 |
| lincRNA | ENST00000478824 | CTD-2377D24.6 | 2.6 (< .05) | TTLL6 | CALCOCO2 |
| lincRNA | ENST00000412526 | LINC00161 | 2.5 (< .05) | ADAMTS5 | N6AMT1 |
| lincRNA | ENST00000420594 | AC073130.1 | 2.4 (< .05) | TES | CAV2 |
| Processed transcript | ENST00000453698 | SNHG11 | 2.4 (< .05) | LBP | RALGAPB |
| Antisense | ENST00000420563 | AC053503.4 | 2.4 (< .05) | DNPEP | DES |
| lincRNA | ENST00000511497 | RP11-420A23.1 | 2.3 (< .05) | PGRMC2 | PHF17 |
| Processed transcript | ENST00000413755 | SNHG17 | 2.3 (< .05) | LBP | RALGAPB |
| lincRNA | ENST00000512322 | RP11-792D21.2 | 2.2 (< .05) | ANXA3 | BMP2K |
| Antisence | ENST00000519104 | RP3-399L15.3 | 2.1 (< .05) | HDAC2 | HS3ST5 |
| lincRNA | ENST00000416534 | AC007463.2 | 2.1 (< .05) | RNF144A | ID2 |
| lincRNA | ENST00000427903 | RP1-60O19.1 | 2.1 (< .05) | QRSL1 | C6orf203 |
| lincRNA | ENST00000442796 | LINC00312 | 2.0 (< .05) | LMCD1 | SSUH2 |
| lincRNA | ENST00000437523 | RP11-344P13.4 | 2.0 (< .05) | FCGR1B | PP1AL4G |
| lincRNA | ENST00000560760 | RP11-38G5.2 | 2.0 (< .05) | KIAA1024 | MTHFS |
| lincRNA | ENST00000454596 | RP11-69I8.2 | 1.9 (< .05) | ENPP1 | CTGF |
| lincRNA | ENST00000366424 | AC144450.2 | 1.8 (< .05) | TPO | PXDN |
| lincRNA | ENST00000412606 | AC096559.1 | 1.8 (< .05) | LPIN1 | TRIB2 |
| Antisence | ENST00000439075 | RP11-510H23.1 | 1.8 (< .05) | NKAIN2 | RNF217 |
| lincRNA | ENST00000440888 | RP11-315I14.2 | 1.8 (< .05) | DMRTA1 | ELAVL2 |
| lincRNA | ENST00000523242 | CTB-43E15.1 | 1.7 (< .05) | BOD1 | CPEB4 |
| lincRNA | ENST00000366185 | RP11-258C19.5 | 1.7 (< .05) | FAM156A | KDM5C |
| Processed transcript | ENST00000426282 | CTA-217C2.1 | 1.7 (< .05) | PHF21B | KIAA0930 |
| lincRNA | ENST00000436112 | RP3-523C21.1 | 1.7 (< .05) | CTGF | MOXD1 |
| lincRNA | ENST00000435967 | RP4-594A5.1 | 1.6 (< .05) | ICA1 | NXPH1 |
| lincRNA | ENST00000488287 | CT64 | 1.6 (< .05) | NMD3 | SPTSSB |
| lincRNA | ENST00000446884 | RP1-30G7.2 | 1.6 (< .05) | CXorf36 | KRBOX4 |
| lincRNA | ENST00000417112 | RP11-554I8.2 | 1.5 (< .05) | PRKCQ | SFMBT2 |
| lincRNA | ENST00000416401 | RP11-77M5.1 | 1.5 (< .05) | RGS5 | NUF2 |
| lincRNA | ENST00000511840 | CTD-2066L21.2 | 1.5 (< .05) | NPR3 | TARS |
| Retained intron | ENST00000436578 | AC005532.5 | 1.5 (< .05) | C1GALT1 | COL28A1 |
| lincRNA | ENST00000430244 | RP11-166O4.5 | 1.5 (< .05) | TYW1 | AUTS2 |
| lincRNA | ENST00000471357 | LINC00880 | 1.5 (< .05) | LEKR1 | CCNL1 |
| lincRNA | ENST00000508147 | RP11-622A1.2 | 1.5 (< .05) | AFM | RASSF6 |
| Sense overlapping | ENST00000530072 | RP11-166D19.1 | -1.5 (< .05) | SORL1 | BLID |
| lincRNA | ENST00000455957 | HCG17 | -1.5 (< .05) | TRIM15 | TRIM39 |
| Retained intron | ENST00000521127 | SNHG6 | -1.5 (< .05) | MCMDC2 | PPP1R42 |
| lincRNA | ENST00000451230 | AC108463.1 | -1.6 (< .05) | BCL2L11 | ZC3H8 |
| lincRNA | ENST00000449259 | AC007386.2 | -1.6 (< .05) | SERTAD2 | SLC1A4 |
| lincRNA | ENST00000453722 | LINC00511 | -1.6 (< .05) | SOX9 | SLC39A11 |
| Processed transcript | ENST00000431616 | LINC00630 | -1.6 (< .05) | BHLHB9 | RAB40AL |
| lincRNA | ENST00000443576 | RP11-141M1.1 | -1.7 (< .05) | STARD13 | RFC3 |
| Processed transcript | ENST00000430756 | RP11-761E20.1 | -1.7 (< .05) | PLS3 | AGTR2 |
| Processed transcript | ENST00000416650 | RSBN1L-AS1 | -1.7 (< .05) | PTPN12 | RSBN1L |
| Sense intronic | ENST00000433544 | RP11-488P3.1 | -1.7 (< .05) | BCAR3 | DNTTIP2 |
| lincRNA | ENST00000415611 | AC005682.5 | -1.7 (< .05) | TOMM7 | FAM126A |
| lincRNA | ENST00000505448 | RP11-774O3.3 | -1.7 (< .05) | HTRA3 | ACOX3 |
| Antisence | ENST00000496733 | TMEM161B-AS1 | -1.8 (< .05) | TMEM161B | MEF2C |
| lincRNA | ENST00000529893 | RP1-80B9.2 | -1.8 (< .05) | GMDS | C6orf195 |
| lincRNA | ENST00000552334 | RP11-701H24.2 | -1.8 (< .05) | SNURF | UBE3A |
| lincRNA | ENST00000515871 | CTC-325J23.3 | -1.8 (< .05) | ANKRD34B | DHFR |
| lincRNA | ENST00000433747 | RP11-120D5.1 | -1.8 (< .05) | MID1 | AMELX |
| lincRNA | ENST00000438762 | AP000473.5 | -1.8 (< .05) | USP25 | CXADR |
| Antisence | ENST00000511571 | RP11-453E17.1 | -1.9 (< .05) | TMPRSS11A | TMPRSS11B |
| Antisence | ENST00000456602 | RBM26-AS1 | -1.9 (< .05) | RBM26 | NDFIP2 |
| lincRNA | ENST00000437261 | AC108066.1 | -1.9 (< .05) | ERBB4 | IKZF2 |
| lincRNA | ENST00000454635 | LINC00963 | -1.9 (< .05) | IER5L | NTMT1 |
| lincRNA | ENST00000433079 | AC058791.2 | -1.9 (< .05) | KLF14 | MKLN1 |
| lincRNA | ENST00000444488 | TPRG1-AS1 | -1.9 (< .05) | LPP | TPRG1 |
| lincRNA | ENST00000505155 | RP11-584P21.2 | -1.9 (< .05) | EPHA5 | CENPC |
| lincRNA | ENST00000444265 | LINC00340 | -2.0 (< .05) | SOX4 | PRL |
| lincRNA | ENST00000537192 | RP11-1038A11.3 | -2.0 (< .05) | KCNA5 | NTF3 |
| lincRNA | ENST00000514791 | RP11-434D9.2 | -2.0 (< .05) | CD180 | PIK3R1 |
| lincRNA | ENST00000508619 | RP3-513G18.2 | -2.0 (< .05) | LRPAP1 | ADRA2C |
| Antisence | ENST00000437157 | RP3-510D11.1 | -2.0 (< .05) | GPR157 | H6PD |
| lincRNA | ENST00000412722 | LINC00427 | -2.1 (< .05) | KATNAL1 | HMGB1 |
| lincRNA | ENST00000398518 | MEG3 | -2.1 (< .05) | DLK1 | RTL1 |
| lincRNA | ENST00000418006 | LINC00940 | -2.1 (< .05) | LRTM2 | DCP1B |
| lincRNA | ENST00000534336 | MALAT1 | -2.2 (< .05) | FRMD8 | SCYL1 |
| Antisence | ENST00000434399 | AC005154.6 | -2.5 (< .05) | GGCT | GARS |
| lincRNA | ENST00000455395 | FTX | -3.7 (< .05) | NAP1L2 | ZCCHC13 |
| Processed transcript | ENST00000451141 | MIAT | -4.3 (< .05) | CRYBB1 | MN1 |

**Figure E1: Effect of dexamethasone and FCS upon asthmatic ASM cell proliferation and IL-6 release.** ASM cells were incubated with dexamethasone (10^-7^ M) for 1 h before being stimulated with FCS (2.5 %) for 24 h. DNA synthesis (*A*), cell viability (*B*), and IL-6 (*C*) release were measured by BrdU ELISA, MTT assay, or DuoSet ELISA respectively. Bars represent mean ± SEM from 9 ASM cell donors. ** p < 0.01; ***/### p < 0.001.

**Figure E2: Transfection efficiency in ASMCs from healthy subjects and severe asthmatics at 8 days.**

IL-6 release (*A*&*B*), or cellular viability (*C*-*F*) were measured by DuoSet ELISA Assay (R&D Systems), or MTT assay (Sigma) respectively at 8 days. Bars represent the means ± SEMs of 9 ASM cell donors. ** P < 0.01; ***P < 0.001.

References

(1) Chung KF, Wenzel SE, Brozek JL, Bush A, Castro M, Sterk PJ et al. International ERS/ATS guidelines on definition, evaluation and treatment of severe asthma. Eur Respir J 2014; 43(2):343-73.

(2) Perry MM, Durham AL, Austin PJ, Adcock IM, Chung KF. BET bromodomains regulate TGF-beta-induced proliferation and cytokine release in asthmatic airway smooth muscle. J Biol Chem 2015.

(3) Perry M, Tsitsiou E, Austin P, Lindsay M, Gibeon D, Adcock I et al. Role of non-coding RNAs in maintaining primary airway smooth muscle cells. Respiratory Research 2014; 15(1):58.

(4) Perry MM, Hui CK, Whiteman M, Wood ME, Adcock I, Kirkham P et al. Hydrogen Sulfide Inhibits Proliferation and Release of IL-8 from Human Airway Smooth Muscle Cells. Am J Respir Cell Mol Biol 2011; 45(4):746-52.

(5) Perry MM, Baker JE, Gibeon DS, Adcock IM, Chung KF. Airway Smooth Muscle Hyperproliferation Is Regulated by MicroRNA-221 in Severe Asthma. Am J Respir Cell Mol Biol 2013; 50(1):7-17.

(6) O'Leary L, Sevinc K, Papazoglou IM, Tildy B, Detillieux K, Halayko AJ et al. Airway Smooth Muscle Inflammation Is Regulated by MicroRNA-145 in COPD. FEBS Lett 2016.

(7) Mosmann T. Rapid colorimetric assay for cellular growth and survival: application to proliferation and cytotoxicity assays. J Immunol Methods 1983; 65(1-2):55-63.

(8) Perry MM, Moschos SA, Williams AE, Shepherd NJ, Larner-Svensson HM, Lindsay MA. Rapid Changes in MicroRNA-146a Expression Negatively Regulate the IL-1beta-Induced Inflammatory Response in Human Lung Alveolar Epithelial Cells. The Journal of Immunology 2008; 180(8):5689-98.

(9) Perry MM, Baker JE, Gibeon DS, Adcock IM, Chung KF. Airway Smooth Muscle Hyperproliferation Is Regulated by MicroRNA-221 in Severe Asthma. Am J Respir Cell Mol Biol 2013; 50(1):7-17.

(10) Larner-Svensson HM, Williams AE, Tsitsiou E, Perry MM, Jiang X, Chung KF et al. Pharmacological studies of the mechanism and function of interleukin-1beta-induced miRNA-146a expression in primary human airway smooth muscle. Respir Res 2010; 11:68.
